# Supplementary material for: Predictors of medical staff’s knowledge, attitudes and behavior of dysphagia assessment: A cross-sectional study
Source: PLoS One. 2024 Apr 5;19(4):e0301770. doi: 10.1371/journal.pone.0301770 (PMC10997058; doi:10.1371/journal.pone.0301770)
Supplement: S1 Table — (DOC) [file pone.0301770.s001.doc]

S1 Table. The scoring methods and rules of the questionnaire. (DOC)

Table 1 The scoring methods and rules of the questionnaire

| Domains | Items | n | scoring methods and rules |
| --- | --- | --- | --- |
| Knowledge | True or False questions | 17 | 1 point is scored for correct answer. 0 point is scored for incorrect answer. |
|  | Single-choice questions | 4 |  |
|  | Multiple-choice questions | 4 | Count 0.2/ option when selecting the correct option. Count 0 when the wrong option was selected or the correct and wrong options were selected simultaneously  For example, a multiple-choice question had three correct choices that score 0.6 when participant selected three correct choices. |
| Attitudes | Likert-five-point-scale | 8 | Likert 5-level scoring method. Ranging from “strongly agreed” to “strongly disagreed”. 5 point for “strongly agreed”, 1 point for “strongly disagreed” |
| Behavior | Likert-five-point-scale | 13 | Likert 5-level scoring method. Ranging from “the most frequently” to “the least frequently. 5point for “the most frequently”, 1 point for “the least frequently |
