# Supplementary material for: Predictors of medical staff’s knowledge, attitudes and behavior of dysphagia assessment: A cross-sectional study
Source: PLoS One. 2024 Apr 5;19(4):e0301770. doi: 10.1371/journal.pone.0301770 (PMC10997058; doi:10.1371/journal.pone.0301770)
Supplement: S2 Table — (DOC) [file pone.0301770.s002.doc]

**S2 Table. Differences of medical staff ’s Attitudes of dysphagia assessment in Sociodemographic, training and working experience characteristics (n=353)**

| **Characteristic** |  | **n(%)** | **A**  **Mean ± SD** | **95%CI** | **Univariate analysis**  **(t/F, p)** |
| --- | --- | --- | --- | --- | --- |
| **Hospital level** | Level 1 | 14 (4.0) | 34.0±4.6 | (31.3,36.7) | F=1.115, p=0.329 |
|  | Level 2 | 53 (15.0) | 35.9±4.7 | (34.6,37.2) |  |
|  | Level 3 | 286 (81.0) | 36.0±4.9 | (35.4,36.6) |  |
| **Hospital type** | The general hospital | 336 (95.2) | 36.0±4.7 | (35.5,36.5) | t=2.586, p=0.010 |
|  | Other hospital | 17 (4.8) | 32.9±6.8 | (29.4,36.4) |  |
| **Department** | Department (Neurology, Rehabilitation, Geriatrics) | 177 (50.1) | 36.9±4.0 | (36.3,37.5) | t=4.144, p<0.001 |
|  | Other department | 176 (49.9) | 34.8±5.4 | (34.0,35.6) |  |
| **Position** | Clinical nurse | 234 (66.3) | 36.3±4.4 | (35.8,36.9) | F=2.701, p=0.031 |
|  | Clinical doctor | 68 (19.3) | 34.2±6.2 | (32.7,35.7) |  |
|  | Management personnel | 34 (9.6) | 36.1±4.8 | (34.4,37.7) |  |
|  | Community nurses | 12 (3.4) | 36.4±4.9 | (33.3,39.6) |  |
|  | Others | 5 (1.4) | 35.4±3.4 | (31.1,39.7) |  |
| **Title** | Primary title | 165 (46.7) | 36.2±4.4 | (35.6,36.9) | F=0.832, p=0.436 |
|  | Medium-grade professional title | 148 (41.9) | 35.6±5.4 | (34.7,36.5) |  |
|  | Senior title of professional | 40 (11.3) | 35.6±4.6 | (34.1,37.0) |  |
| **Working years in the field of dysphagia related diseases** | None | 113 (32.0) | 34.2±5.5 | (33.2,35.3) | F=6.803, p<0.001 |
|  | <3 years | 63 (17.9) | 36.4±4.6 | (35.3,37.6) |  |
|  | 3-5 years | 54 (15.3) | 36.9±4.1 | (35.7,38.0) |  |
|  | ≥5 years | 123 (34.8) | 36.7±4.3 | (35.9,37.5) |  |
| **Education** | Junior college and below | 62 (17.5) | 36.8±4.2 | (35.7,37.9) | F=4.861, p=0.008 |
|  | Bachelor | 253 (71.7) | 36.0±4.7 | (35.4,36.6) |  |
|  | Master degree or above | 38 (10.8) | 33.8±6.1 | (31.8,35.8) |  |
| **Experience in nursing patients with dysphagia** | Yes | 234 (66.3) | 36.7±4.2 | (36.1,37.2) | t=3.965, p<0.001 |
|  | No | 119 (33.7) | 34.3±5.7 | (33.3,35.4) |  |
| **Related training for dysphagia** | Yes | 175 (49.6) | 37.1±4.0 | (36.5,37.7) | t=4.637, p<0.001 |
|  | No | 178 (50.4) | 34.7±5.4 | (33.9,35.5) |  |
| **Specialized training (geriatric, swallowing and rehabilitation)** | Yes | 55 (15.6) | 38.1±3.1 | (37.3,39.0) | t=3.780, p<0.001 |
|  | No | 298 (84.4) | 35.5±5.0 | (34.9,36.1) |  |

Note.Abbreviation: CI=confidence interval, A=Attitudes.
