# Supplementary material for: Predictors of medical staff’s knowledge, attitudes and behavior of dysphagia assessment: A cross-sectional study
Source: PLoS One. 2024 Apr 5;19(4):e0301770. doi: 10.1371/journal.pone.0301770 (PMC10997058; doi:10.1371/journal.pone.0301770)
Supplement: S3 Table — (DOC) [file pone.0301770.s003.doc]

**S3 Table. Differences of medical staff ’s Behavior of dysphagia assessment in Sociodemographic, training and working experience characteristics (n=353)**

| **Characteristic** |  | **n(%)** | **B**  **Mean ± SD** | **95%CI** | **Univariate analysis**  **(t/F, p)** |
| --- | --- | --- | --- | --- | --- |
| **Hospital level** | Level 1 | 14 (4.0) | 33.6±13.2 | (25.9,41.2) | F=4.054, p=0.018 |
|  | Level 2 | 53 (15.0) | 38.3±16.4 | (33.7,42.8) |  |
|  | Level 3 | 286 (81.0) | 42.4±13.9 | (40.8,44.0) |  |
| **Hospital type** | The general hospital | 336 (95.2) | 42.0±14.3 | (40.5,43.5) | t=3.305, p=0.001 |
|  | Other hospital | 17 (4.8) | 30.3±13.5 | (23.4,37.2) |  |
| **Department** | Department (Neurology, Rehabilitation, Geriatrics) | 177 (50.1) | 47.8±11.1 | (46.1,49.4) | t=9.255, p<0.001 |
|  | Other department | 176 (49.9) | 35.0±14.6 | (32.8,37.2) |  |
| **Position** | Clinical nurse | 234 (66.3) | 42.1±14.3 | (40.3,43.9) | F=1.286, p=0.275 |
|  | Clinical doctor | 68 (19.3) | 41.3±14.4 | (37.8,44.8) |  |
|  | Management personnel | 34 (9.6) | 40.3±13.0 | (35.8,44.9) |  |
|  | Community nurses | 12 (3.4) | 36.2±19.1 | (24.0,48.3) |  |
|  | Others | 5 (1.4) | 30.6±14.0 | (13.2,48.0) |  |
| **Title** | Primary title | 165 (46.7) | 41.0±15.2 | (38.7,43.4) | F=0.170, p=0.844 |
|  | Medium-grade professional title | 148 (41.9) | 41.6±13.8 | (39.3,43.8) |  |
|  | Senior title of professional | 40 (11.3) | 42.5±13.7 | (38.1,46.8) |  |
| **Working years in the field of dysphagia related diseases** | None | 113 (32.0) | 33.6±14.0 | (31.0,36.3) | F=20.964, p<0.001 |
|  | <3 years | 63 (17.9) | 41.5±15.5 | (37.6,45.4) |  |
|  | 3-5 years | 54 (15.3) | 46.1±13.2 | (42.4,49.7) |  |
|  | ≥5 years | 123 (34.8) | 46.5±11.4 | (44.5,48.5) |  |
| **Education** | Junior college and below | 62 (17.5) | 38.4±17.3 | (34.1,42.8) | F=1.660, p=0.192 |
|  | Bachelor | 253 (71.7) | 42.0±13.7 | (40.3,43.7) |  |
|  | Master degree or above | 38 (10.8) | 42.7±13.6 | (38.2,47.2) |  |
| **Experience in nursing patients with dysphagia** | Yes | 234 (66.3) | 45.4±12.9 | (43.8,47.1) | t=7.939, p<0.001 |
|  | No | 119 (33.7) | 33.5±14.1 | (31.0,36.1) |  |
| **Related training for dysphagia** | Yes | 175 (49.6) | 47.0±12.4 | (45.1,48.8) | t=7.785, p<0.001 |
|  | No | 178 (50.4) | 35.9±14.2 | (33.8,38.0) |  |
| **Specialized training (geriatric, swallowing and rehabilitation)** | Yes | 55 (15.6) | 51.7±10.1 | (49.0,54.5) | t=7.669, p<0.001 |
|  | No | 298 (84.4) | 39.5±14.3 | (37.9,41.1) |  |

Note.Abbreviation: CI=confidence interval, B=Behavior.
