# Supplementary material for: Predictors of medical staff’s knowledge, attitudes and behavior of dysphagia assessment: A cross-sectional study
Source: PLoS One. 2024 Apr 5;19(4):e0301770. doi: 10.1371/journal.pone.0301770 (PMC10997058; doi:10.1371/journal.pone.0301770)
Supplement: S4 Table — (DOC) [file pone.0301770.s004.doc]

**S4 Table. Dummy variable settings**

| the original variable |  | D1 | D2 | D3 | D4 | state |
| --- | --- | --- | --- | --- | --- | --- |
| hospital level | level 1 | 1 | 0 |  |  |  |
|  | level 2 | 0 | 0 |  |  | reference category |
|  | level 3 | 0 | 1 |  |  |  |
| hospital type | the general hospital | 1 |  |  |  |  |
|  | other hospital | 0 |  |  |  | reference category |
| department | department (Neurology, Rehabilitation, Geriatrics) | 1 |  |  |  |  |
|  | other department | 0 |  |  |  | reference category |
| position | clinical nurse | 1 | 0 | 0 | 0 |  |
|  | clinical doctor | 0 | 0 | 0 | 0 | reference category |
|  | Management personnel | 0 | 0 | 1 | 0 |  |
|  | Community nurses | 0 | 0 | 0 | 1 |  |
|  | others | 0 | 1 | 0 | 0 |  |
| title | primary title | 1 | 0 |  |  |  |
|  | medium-grade professional title | 0 | 0 |  |  | reference category |
|  | senior title of professional | 0 | 1 |  |  |  |
| working years in the field of dysphagia related diseases | none | 1 | 0 | 0 |  |  |
|  | <3 years | 0 | 0 | 0 |  | reference category |
|  | 3-5 years | 0 | 1 | 0 |  |  |
|  | ≥5 years | 0 | 0 | 1 |  |  |
| education | Junior college and below | 1 | 0 |  |  |  |
|  | Bachelor | 0 | 0 |  |  | reference category |
|  | Master degree or above | 0 | 1 |  |  |  |
| experience in nursing patients with dysphagia | yes | 1 |  |  |  |  |
|  | no | 0 |  |  |  | reference category |
| related training for dysphagia | yes | 1 |  |  |  |  |
|  | no | 0 |  |  |  | reference category |
| specialized training in geriatric, swallowing and rehabilitation | yes | 1 |  |  |  |  |
|  | no | 0 |  |  |  | reference category |

Note.D: dummy variable
