# Supplementary material for: Predictors of medical staff’s knowledge, attitudes and behavior of dysphagia assessment: A cross-sectional study
Source: PLoS One. 2024 Apr 5;19(4):e0301770. doi: 10.1371/journal.pone.0301770 (PMC10997058; doi:10.1371/journal.pone.0301770)
Supplement: S5 Table — (DOC) [file pone.0301770.s005.doc]

**S5 Table. Factors related to Knowledge of medical staff by stepwise regression**

| **Variables** | **Std. β** | **t** | **p value** |
| --- | --- | --- | --- |
| **Working years in the field of dysphagia related diseases (ref: <3 years): none** | -0.066 | -1.025 | 0.306 |
| **Working years in the field of dysphagia related diseases (ref: <3 years): 3-5 years** | 0.016 | 0.283 | 0.777 |
| **Working years in the field of dysphagia related diseases (ref: <3 years): ≥5 years** | 0.184 | 3.379 | 0.001 |
| **Department (Neurology, Rehabilitation, Geriatrics) (ref: No): Yes** | 0.138 | 2.558 | 0.011 |
| **Title (ref: Medium-grade professional title): Primary title** | -0.087 | -1.530 | 0.127 |
| **Title (ref: Medium-grade professional title): Senior title of professional** | 0.132 | 2.495 | 0.013 |

note. Std. β, standardized βcoefficient

Adjusted R^2^=0.078, F=10.964, p<0.001
