# Supplementary material for: Predictors of medical staff’s knowledge, attitudes and behavior of dysphagia assessment: A cross-sectional study
Source: PLoS One. 2024 Apr 5;19(4):e0301770. doi: 10.1371/journal.pone.0301770 (PMC10997058; doi:10.1371/journal.pone.0301770)
Supplement: S6 Table — (DOC) [file pone.0301770.s006.doc]

**S6 Table. Factors related to Attitudes of of medical staff by stepwise regression**

| **Variables** | **Std. β** | **t** | **p value** |
| --- | --- | --- | --- |
| **Education (ref: Bachelor): Junior college and below** | 0.144 | 2.734 | 0.007 |
| **Education (ref: Bachelor): Master degree or above** | -0.127 | -2.498 | 0.013 |
| **Experience in nursing patients with dysphagia(ref: No): Yes** | 0.113 | 1.976 | 0.049 |
| **Department (Neurology, Rehabilitation, Geriatrics) (ref: No): Yes** | 0.123 | 1.995 | 0.047 |
| **Related training for dysphagia(ref: No): Yes** | 0.148 | 2.723 | 0.007 |

note. Std. β, standardized βcoefficient

Adjusted R^2^=0.129, F=9.724, p<0.001
