# Supplementary material for: Predictors of medical staff’s knowledge, attitudes and behavior of dysphagia assessment: A cross-sectional study
Source: PLoS One. 2024 Apr 5;19(4):e0301770. doi: 10.1371/journal.pone.0301770 (PMC10997058; doi:10.1371/journal.pone.0301770)
Supplement: Attachment — Submitted filename: PONE-D-23-41544_R2.pdf [file pone.0301770.s011.pdf]

PLOS ONE

Predictors of medical staff's Knowledge, Attitudes and Behavior of dysphagia assessment: A Cross-Sectional study

--Manuscript Draft--

**Manuscript Number:**	PONE-D-23-41544R1
**Article Type:**	Research Article
**Full Title:**	Predictors of medical staff's Knowledge, Attitudes and Behavior of dysphagia assessment: A Cross-Sectional study
**Short Title:**	Predictors of medical staff's KAP of dysphagia assessment
**Corresponding Author:**	Juanhui Chen<br>Longgang Central Hospital of Shenzhen<br>Shenzhen, Guangdong province CHINA
**Keywords:**	assessment, Attitude, Behavior, Cross-Sectional, dysphagia, Knowledge, Predictors, stroke
**Abstract:**	Purpose: This study aimed to explore the predictors of medical staff's Knowledge, Attitudes and Behavior of dysphagia assessment, to provide reference suggestions for constructing the training program and improving the rate of dysphagia assessment.<br>Methods: This study was a cross-sectional study. A total of 353 nurses and doctors from four provinces (Guangdong, Hunan, Guangxi, and Shaanxi) who were selected by convenience sampling and invited to complete the questionnaire through WeChat, DingTalk, and Tencent instant messenger from May 23 to 31, 2022. A self-reported questionnaire was used to assess participants' Knowledge, Attitude and Behavior regarding dysphagia assessment. Participants' sociodemographic, training, and nursing experience were measured using the general information sheet and analyzed as potential predictors of medical staff's Knowledge, Attitudes and Behavior of dysphagia assessment. A multiple linear regression model was used to identify the predictors.<br>Results: The mean score for Knowledge, Attitudes and Behavior of dysphagia assessments was (92.7±17.5). Multiple linear regression results indicated that experience in nursing patients with dysphagia, related training for dysphagia, working years in the field of dysphagia related diseases, specialized training (geriatric, swallowing and rehabilitation) and department (Neurology, Rehabilitation, Geriatrics) were significant predictors, accounting for 35.1% of the variance.<br>Conclusions: The study findings implied that nursing experience, training, and work for patients with swallowing disorders could have positive effects on the Knowledge, Attitudes and Behavior of medical staff regarding dysphagia assessment. Hospital administrators should provide relevant resources, such as videos of dysphagia assessment, training centers for the assessment of dysphagia, and swallowing specialist nurses. It is important that health policies fully recognize the role of training and support systems in caring for people with dysphagia.
**Order of Authors:**	Juanhui Chen
	Wenqiu Ye
	Xingyun Zheng
	Wenna Wu
	Yuebao Chen
	Yinjuan Chen
**Response to Reviewers:**	Thank you for your letter and for the reviewer's comments concerning our manuscript entitled "Predictors of medical staff's Knowledge, Attitudes and Behavior of dysphagia assessment: A Cross-Sectional study"(ID:PONE-D-23-41544). Those comments are all valuable and very helpful for revising and improving our paper, as well as the important guiding significance to our researches. We have studied comments carefully and have made correction which we hope meet with approval. Please find a file named response to reviewers in attach file.

Powered by Editorial Manager® and ProduXion Manager® from Aries Systems Corporation

Additional Information:	
**Question**	**Response**
**Financial Disclosure**<br><br>Enter a financial disclosure statement that describes the sources of funding for the work included in this submission. Review the submission guidelines for detailed requirements. View published research articles from *PLOS ONE* for specific examples.<br><br>This statement is required for submission and **will appear in the published article** if the submission is accepted. Please make sure it is accurate.<br><br>**Funded studies**<br>Enter a statement with the following details:<br>• Initials of the authors who received each award<br>• Grant numbers awarded to each author<br>• The full name of each funder<br>• URL of each funder website<br>• Did the sponsors or funders play any role in the study design, data collection and analysis, decision to publish, or preparation of the manuscript?<br><br>Did you receive funding for this work?	Yes
Please add funding details.<br>as follow-up to "**Financial Disclosure**<br><br>Enter a financial disclosure statement that describes the sources of funding for the work included in this submission. Review the submission guidelines for detailed requirements. View published research articles from *PLOS ONE* for specific examples.<br><br>This statement is required for submission and **will appear in the published article** if the submission is accepted. Please make sure it is accurate.	Medical and Health Technology Plan of Longgang District, Shenzhen. Funding was rewarded to Juanhui Chen. The Grant award number was LGKCYLWS2020128. URL: http://www.lg.gov.cn/bmzz/kjj/xxgk/qt/tzgg/content/post_7970194.html The funders play no role in the study design, data collection and analysis, decision to publish, or preparation of the manuscript.

Powered by Editorial Manager® and ProduXion Manager® from Aries Systems Corporation

**Funded studies**<br>Enter a statement with the following details:<br>• Initials of the authors who received each award<br>• Grant numbers awarded to each author<br>• The full name of each funder<br>• URL of each funder website<br>• Did the sponsors or funders play any role in the study design, data collection and analysis, decision to publish, or preparation of the manuscript?<br><br>Did you receive funding for this work?"	
Please select the country of your main research funder (please select carefully as in some cases this is used in fee calculation).<br>as follow-up to "**Financial Disclosure**<br><br>Enter a financial disclosure statement that describes the sources of funding for the work included in this submission. Review the submission guidelines for detailed requirements. View published research articles from *PLOS ONE* for specific examples.<br><br>This statement is required for submission and **will appear in the published article** if the submission is accepted. Please make sure it is accurate.<br><br>**Funded studies**<br>Enter a statement with the following details:<br>• Initials of the authors who received each award<br>• Grant numbers awarded to each author<br>• The full name of each funder<br>• URL of each funder website<br>• Did the sponsors or funders play any role in the study design, data collection and analysis, decision to publish, or preparation of the manuscript?<br><br>Did you receive funding for this work?"	
**Competing Interests**	The authors have declared that no competing interests exist.

Use the instructions below to enter a competing interest statement for this submission. On behalf of all authors, disclose any competing interests that could be perceived to bias this work—acknowledging all financial support and any other relevant financial or non-financial competing interests.<br><br>This statement is required for submission and **will appear in the published article** if the submission is accepted. Please make sure it is accurate and that any funding sources listed in your Funding Information later in the submission form are also declared in your Financial Disclosure statement.<br><br>View published research articles from *PLOS ONE* for specific examples.<br><br>**NO authors have competing interests**<br><br>Enter: *The authors have declared that no competing interests exist.*<br><br>**Authors with competing interests**<br><br>Enter competing interest details beginning with this statement:<br><br>*I have read the journal's policy and the authors of this manuscript have the following competing interests: [insert competing interests here]*<br><br>* typeset	
**Ethics Statement**<br><br>Enter an ethics statement for this submission. This statement is required if the study involved:<br><br>• Human participants<br>• Human specimens or tissue<br>• Vertebrate animals or cephalopods<br>• Vertebrate embryos or tissues	This study was approved by the Ethics Committee of Longgang Central Hospital of Shenzhen(No. 2022ECPJ101)

Powered by Editorial Manager® and ProduXion Manager® from Aries Systems Corporation

• Field research<br><br>Write "N/A" if the submission does not require an ethics statement.<br><br>General guidance is provided below. Consult the submission guidelines for detailed instructions. **Make sure that all information entered here is included in the Methods section of the manuscript.**	

**Format for specific study types**<br><br>**Human Subject Research (involving human participants and/or tissue)**<br>• Give the name of the institutional review board or ethics committee that approved the study<br>• Include the approval number and/or a statement indicating approval of this research<br>• Indicate the form of consent obtained (written/oral) or the reason that consent was not obtained (e.g. the data were analyzed anonymously)<br><br>**Animal Research (involving vertebrate animals, embryos or tissues)**<br>• Provide the name of the Institutional Animal Care and Use Committee (IACUC) or other relevant ethics board that reviewed the study protocol, and indicate whether they approved this research or granted a formal waiver of ethical approval<br>• Include an approval number if one was obtained<br>• If the study involved *non-human primates*, add *additional details* about animal welfare and steps taken to ameliorate suffering<br>• If anesthesia, euthanasia, or any kind of animal sacrifice is part of the study, include briefly which substances and/or methods were applied<br><br>**Field Research**<br><br>Include the following details if this study involves the collection of plant, animal, or other materials from a natural setting:<br>• Field permit number<br>• Name of the institution or relevant body that granted permission	
**Data Availability**<br><br>Authors are required to make all data underlying the findings described fully available, without restriction, and from the time of publication. PLOS allows rare exceptions to address legal and ethical concerns. See the PLOS Data Policy and FAQ for detailed information.	Yes - all data are fully available without restriction

A Data Availability Statement describing where the data can be found is required at submission. Your answers to this question constitute the Data Availability Statement and **will be published in the article**, if accepted.<br><br>**Important:** Stating 'data available on request from the author' is not sufficient. If your data are only available upon request, select 'No' for the first question and explain your exceptional situation in the text box.<br><br>Do the authors confirm that all data underlying the findings described in their manuscript are fully available without restriction?	
**Describe where the data may be found in full sentences. If you are copying our sample text, replace any instances of XXX with the appropriate details.**<br><br>• If the data are **held or will be held in a public repository**, include URLs, accession numbers or DOIs. If this information will only be available after acceptance, indicate this by ticking the box below. For example: *All XXX files are available from the XXX database (accession number(s) XXX, XXX.).*<br>• If the data are all contained **within the manuscript and/or Supporting Information files**, enter the following: *All relevant data are within the manuscript and its Supporting Information files.*<br>• If neither of these applies but you are able to provide **details of access elsewhere**, with or without limitations, please do so. For example:<br><br>*Data cannot be shared publicly because of [XXX]. Data are available from the XXX Institutional Data Access / Ethics Committee (contact via XXX) for researchers who meet the criteria for access to confidential data.*<br><br>*The data underlying the results presented in the study are available from (include the name of the third party*	Data Availability Statement: Data is deposited at Dryad (DOI: https://doi.org/10.5061/dryad.djh9w0w70).

and contact information or URL).<br>• This text is appropriate if the data are owned by a third party and authors do not have permission to share the data.<br><br>* typeset	
Additional data availability information:	Tick here if the URLs/accession numbers/DOIs will be available only after acceptance of the manuscript for publication so that we can ensure their inclusion before publication.

Powered by Editorial Manager® and ProduXion Manager® from Aries Systems Corporation

Manuscript Click here to access/download;Manuscript;Revised Manuscript.doc

Predictors of medical staff's Knowledge, Attitudes and Behavior of dysphagia assessment: A Cross-Sectional study

Juanhui Chen[1*], Wenqiu Ye[2], Xingyun Zheng[3], Wenna Wu[4], Yuebao Chen[5], Yinjuan Chen[6]

1 Department of Nursing, Longgang Central Hospital of Shenzhen, Shenzhen, Guangdong province, China. 2 Department of Neurology, Longgang Central Hospital of Shenzhen, Shenzhen, Guangdong province, China. 3 Department of Neurosurgery, Longgang Central Hospital of Shenzhen, Shenzhen, Guangdong province, China. 4 Department of Rehabilitation, Longgang Central Hospital of Shenzhen, Shenzhen, Guangdong province, China. 5 Department of Neurology, Guangxi International Zhuang Medicine hospital, Nanning, Guangxi province, China. 6 Department of Nursing, Shenzhen FuYong People's Hospital, Shenzhen, Guangdong province, China.

Running head: Predictors of medical staff's KAP of dysphagia assessment

***Correspondence**

E-mail:pugongyingchenjuan@163.com (JC)

.

Abstract

Purpose: This study aimed to explore the predictors of medical staff's Knowledge, Attitudes and Behavior of dysphagia assessment, to provide reference suggestions for constructing the training program and improving the rate of dysphagia assessment..

Methods: This study was a cross-sectional study. A total of 353 nurses and doctors from four provinces (Guangdong, Hunan, Guangxi, and Shaanxi) who were selected by convenience sampling and invited to complete the questionnaire through WeChat, DingTalk, and Tencent instant messenger from May 23 to 31, 2022. A self-reported questionnaire was used to assess participants' Knowledge, Attitude and Behavior regarding dysphagia assessment. Participants' sociodemographic, training, and nursing experience were measured using the general information sheet and analyzed as potential predictors of medical staff's Knowledge, Attitudes and Behavior of dysphagia assessment. A multiple linear regression model was used to identify the predictors.

Results: The mean score for Knowledge, Attitudes and Behavior of dysphagia assessments was (92.7±17.5). Multiple linear regression results indicated that experience in nursing patients with dysphagia, related training for dysphagia, working years in the field of dysphagia related diseases, specialized training (geriatric, swallowing and rehabilitation) and department (Neurology, Rehabilitation, Geriatrics) were significant predictors, accounting for 35.1% of the variance.

Conclusions: The study findings implied that nursing experience, training, and work for patients with swallowing disorders could have positive effects on the

Knowledge, Attitudes and Behavior of medical staff regarding dysphagia assessment. Hospital administrators should provide relevant resources, such as videos of dysphagia assessment, training centers for the assessment of dysphagia, and swallowing specialist nurses. It is important that health policies fully recognize the role of training and support systems in caring for people with dysphagia.

Abbreviations: KAB=Knowledge, Attitudes and Behavior, MS-KAB-DA=Questionnaire of Medical Staff's Knowledge, Attitudes and Behavior of dysphagia assessment.

Key words: assessment, Attitude, Behavior, Cross-Sectional, dysphagia, Knowledge, Predictors, stroke

Introduction

Worldwide, there are approximately 80.1 million stroke survivors, with an estimated 13.7 million new cases of stroke every year. Stroke remained the second-leading cause of death and the third-leading cause of death and disability combined[1]. Dysphagia occurs in 30.0–78.0% of patients after stroke[2-4]. Dysphagia may be defined as the partial or complete inability to prepare and move a bolus of food, fluids, or saliva efficiently and safely from the mouth to the esophagus and stomach[5]. Oropharyngeal Dysphagia(OD) is prevalent in the elderly and people with complex medical conditions; for example, aspiration pneumonia is caused by silent aspiration in stroke patients, resulting in considerable medical psychosocial consequences, reduced quality of life, and affected prognosis of patients. Prevalence estimates for OD determined by a meta-analysis were 36.5% in hospital settings, 42.5% in rehabilitation settings, and 50.2% in nursing homes[6].

The type and combination of screening and assessment methods used to determine the prevalence of OD varies. More than half of the studies reported OD prevalence data using screening and clinical non-instrumental assessment methods or tools that were either designed by the authors for the purpose of the study or modified versions of published tools. Thus lacking information on diagnostic performance and psychometric properties[7-9]. However, instrumental assessments require specialized training and equipment because of feasibility (e.g., availability, ease of administration). Screening and clinical non-instrumental assessments are the natural first choice for estimating the prevalence of OD[10]. Speech-language pathologists and other specialists, in collaboration with family physicians, can provide structured assessments and make appropriate recommendations for safe swallowing[11]. One study's results suggested that continuing education hours were significantly associated with dysphagia screening protocol choice[12]. One study evaluated nurses' barriers to compliance with dysphagia measures. And it demonstrated that a knowledge deficit was an important barrier in dysphagia care for nurses, and this can be improved with short training[13].

Studies on the factors contributing to barriers to the assessment of dysphagia are lacking. Some Chinese researchers investigated the practice and management of

medical staff's dysphagia assessment, it showed that More than 96.0% of the nurses did not know anything about the swallowing function training, and only 36.7% of the nurses used the scale to screen the patients for swallowing disorders[14]. And these studies did not explore the influencing factors. We aimed to explore factors contributing to barriers to the assessment of dysphagia, to provide reference suggestions for constructing the training program and improving the rate of dysphagia assessment by conducting this survey.

Methods

Design and Participants

This was a cross-sectional study. A total of 430 nurses and doctors were selected by convenience sampling from four provinces (Guangdong, Hunan, Guangxi, and Shaanxi) .The inclusion criteria were as follows: (a) Chinese nurse practitioners, doctors, or therapists with a medical background, (b) working at the hospital for more than 3 months, and (c) not internship.

According to the sample content estimation method proposed by Kendall, the sample content of questionnaire survey should be 5-10 times the number of items[15]. There were 46 items in the questionnaire. Thus, 230-460 participants were calculated according to the ratio. Adding a 15.0% attrition rate, the total participants sample should between 265 and 529. A total of 353 nurses and doctors completed the survey in this study.

An anonymous survey was conducted by Longgang central hospital of Shenzhen on May 23-31, 2022. Participants were informed about the aims and content of the study and the importance of enrollment. A total of 430 nurses and doctors completed the questionnaire, corresponding to a response rate of 100.0%. The valid questionnaire was 82.1%, and invalid questionnaires were eliminated. The criteria for eliminating invalid questionnaires were (a) incomplete questionnaires, (b) a questionnaire with incorrect content, (c) logically contradictory questionnaires, and (d) time to fill in the questionnaire is shorter than 300 seconds and longer than 3600 seconds.

Ethical statements

This study was approved by Ethics Committee of Longgang Central Hospital of Shenzhen (No. 2022ECPJ101). The first part of the questionnaire mainly included informed consent, participants will read the informed consent at first, if they agree to participate in the study they will chose “I agree” to complete the following survey. The questionnaire survey was anonymous and personal information was not disclosed.

Research instruments

The following instrument covered two parts: .

a) The general information sheet. Demographic characteristics were measured using the general information sheet, which includes 11 items: province, hospital level and type, department, position, title, working years in the field of dysphagia-related

diseases, education, experience of nursing patients with dysphagia, related training for dysphagia, specialized training (geriatric, swallowing and rehabilitation).

b) Questionnaire on medical staff's Knowledge, Attitudes and Behavior of dysphagia assessment. This questionnaire was revised based on the existing questionnaires designed by Dr. Xiaofang Dong[16] and Master Keke Ma[17] from The First Affiliated Hospital of Zhengzhou University. Before the study, we obtained the consent of the author of the original questionnaire to add the scoring method and paraphrase the items of knowledge dimension on the basis of the original questionnaire. The KAB was shortened for Knowledge, Attitudes and Behavior. The MS-KAB-DA was shortened for the Questionnaire of Medical staff's Knowledge, Attitudes, and Behavior of dysphagia assessment. The questionnaire was composed of three specific domains, namely Knowledge (25 items), Attitude (8 items), and Behavior (13 items). The knowledge domain consisted of 17 True or False questions (KR-20=0.760), four single-choice questions, and four multiple-choice questions. The scoring methods and rules of the questionnaire were shown in S1 Table. The total full score of Knowledge, Attitude and Behavior were 24.4, 40 and 65, respectively. Higher scores indicated higher cognitive, willingness and practice levels. Internal consistency estimates were shown to have acceptable reliability for the questionnaire before the survey (Cronbach's alpha for domain K, A, and B are 0.77, 0.97 and 0.91, respectively).

The instrument was administered in Chinese language and designed as a google form, the website was https://www.wjx.cn/vm/mc5kOo1.aspx.

Data collection and Analysis

All participants were invited to complete the google form through WeChat, DingTalk, and Tencent instant messenger. A professional questionnaire survey platform which provides functions equivalent to Amazon Mechanical Turk called "Wenjuan Xing" was used to investigate. The researcher sent the google form to colleagues and classmates to fill in and asked them to forward to their colleagues. Data were analyzed using SPSS for Windows, version 23. Statistical significance was set at $p < 0.05$. The findings were summarized using descriptive statistics, univariate analysis, and dummy multiple regression analysis. The data were normally distributed by skewness and kurtosis tests. Descriptive statistics (e.g., mean±SD , frequency & %) were used to summarize the study variables. Univariate analysis (independent-samples t-test and one-way analysis of variance for categorical independent variables) was performed to explore the potential predictors of medical staff KAB for dysphagia assessment. A comparison analysis was conducted to analyze the differences in medical staff's KAB for dysphagia assessment among four Provinces.

Results

Characteristics of the Participants

A total of 430 nurses and doctors were enrolled, of which 353 nurses and doctors were valid. Clinical nurses, clinicians, and managers accounted for 66.3%, 19.3%, and 9.6%, respectively. Of the included hospitals, 81.0% were tertiary hospitals. 12 community nurses were all from Guangdong Province. 10 community nurses were from level 3 hospital, only 2 from level 1 hospital. 7 community nurses (58.3%) had no working experience in the field of dysphagia related diseases and also had no related training for dysphagia. Other characteristics, such as department, medical staff work, and learning experience are summarized in Table 1.

Table 1. Demographic characteristics of the study participants (n=353)

**Participants'characteristics**		**Guangdong n (%)**	**Hunan n (%)**	**Shaanxi n (%)**	**Guangxi n (%)**
**Hospital level**	Level 1	13 (6.2)	1 (1.3)	0 (0.0)	0 (0.00)
	Level 2	49 (23.2)	3 (3.4)	0 (0.0)	1 (4.0)
	Level 3	149 (70.6)	73 (95.3)	40 (100.0)	24 (96.0)
**Hospital type**	The general hospital	194 (91.9)	77 (100.0)	40 (100.0)	25(100.0)
	Other hospital	17 (8.1)	0 (0.0)	0 (0.0)	0 (0.0)
**Department**	Department (Neurology, Rehabilitation, Geriatrics)	80 (37.9)	39 (50.6)	38 (95.0)	20 (80.0)
	Other department	131 (62.1)	38 (49.4)	2 (5.0)	5 (20.0)
**Position**	Clinical nurse	131 (62.1)	51 (66.2)	38 (95.0)	14 (56.0)
	Clinical doctor	35 (16.6)	23 (29.9)	0 (0.0)	10 (40.0)
	Management personnel	30 (14.2)	2 (2.6)	1 (2.5)	1 (4.0)
	Community nurses	12 (5.7)	0 (0.0)	0 (0.0)	0 (0.0)
	Others	3 (1.4)	1 (1.3)	1 (2.5)	0 (0.0)
**Title**	Primary title	94 (44.6)	36 (46.8)	23 (57.5)	12 (48.0)
	Medium-grade professional title	91 (43.1)	30 (38.9)	17 (42.5)	10 (40.0)
	Senior title of professional	26 (12.3)	11 (14.3)	0 (0.0)	3 (12.0)
**Working years in the field of dysphagia related diseases**	None	87 (41.2)	13 (16.9)	7 (17.5)	6 (24.0)
	<3 years	32 (15.2)	24 (31.1)	5 (12.5)	2 (8.0)
	3-5 years	28 (13.3)	12 (15.6)	8 (20.0)	6 (24.0)
	≥5 years	64 (30.3)	28 (36.4)	20 (50.0)	11 (44.0)
**Education**	Junior college and below	43 (20.4)	18 (23.4)	0 (0.0)	1 (4.0)
	Bachelor	145 (68.7)	53 (68.8)	39 (97.5)	16 (64.0)
	Master degree or above	23 (10.9)	6 (7.8)	1 (2.5)	8 (32.0)
**Experience in**	Yes	137 (64.9)	44 (57.1)	35 (87.5)	18 (72.0)

**nursing patients with dysphagia**	No	74 (35.1)	33 (42.9)	5 (12.5)	7 (28.0)
**Related training for dysphagia**	Yes	109 (51.7)	28 (36.4)	29 (72.5)	9 (36.0)
	No	102 (48.3)	49 (63.6)	11 (27.5)	16 (64.0)
**Specialized training (geriatric, swallowing and rehabilitation)**	Yes	31 (14.7)	6 (7.8)	14 (35.0)	4 (16.0)
	No	180 (85.3)	71 (92.2)	26 (65.0)	21 (84.0)

KAB scores

KAB scores and their standardized scores by percentage were listed in Table 2. The knowledge and behavior scores were medium, but the attitude scores were high.

Table 2. Participants' scores of Knowledge, Attitudes and Behavior in dysphagia assessment

Instruments and score range	Mean±SD	Percentige rating score	Minimum	Maximum	Range
**Medical staff's Knowledge of dysphagia assessment (0-24.4)**	15.3±2.7	62.9	3.6	22.2	18.6
**Medical staff's Attitude of dysphagia assessment(8-40)**	35.9±4.9	89.7	8.0	40.0	32.0
**Medical staff's Behavior of dysphagia assessment(13-65)**	41.4±14.4	63.7	13.0	65.0	52.0
**Medical staff's KAB of dysphagia assessment(21-129.4)**	92.7±17.5	71.6	37.0	125.0	88.0

Note. KAB=Knowledge, Attitudes and Behavior

KAB Differences in 4 province

As seen in Table 3, medical staff in Shaanxi province were more willing to assess dysphagia and assessed more frequently than other provinces.

Table 3. Differences of medical staff 's Knowledge, Attitudes and Behavior of dysphagia assessment in different province(one-way analysis of variance)

Instruments and subdomains	Guangdong	Hunan	Guangxi	Shaanxi	F	p values
	Mean±SD					
**Medical staff's Knowledge of dysphagia assessment(0-24.4)**	15.4±2.5	14.9±2.4	16.6±2.5	15.2±3.7	2.519	0.058
**Medical staff's Attitude of dysphagia assessment(8-40)**	35.7±5.1	35.2±4.7	37.1±4.1	37.7±4.0	3.023	0.030*
**Medical staff's Behavior of dysphagia assessment(13-65)**	40.8±15.5	38.5±12.0	43.5±13.1	49.1±11.1	5.273	0.001**
**Medical staff's KAB of**	91.8±18.7	88.6±14.2	97.2±14.6	102.0±14.7	6.080	<0.001***

dysphagia assessment(21-129.4)

Note. Results of post hoc multiple comparisons between provinces

*Medical staff in Shaanxi province were more willing to assess dysphagia than Guangdong(p=0.037) and Hunan(p=0.023)

**Medical staff in Shaanxi province were more frequently evaluated for dysphagia than Guangdong(p=0.001) and Hunan(p<0.001)

***The scores of the questionnaire in Shaanxi province was higher than Guangdong(p=0.002) and Hunan(p<0.001)

KAB=Knowledge, Attitudes and Behavior

Univariate analysis

KAB scores were compared among the different demographic subgroups. Detailed findings were presented in Table 4. The KAB of medical staff was not significantly different between positions, titles, and education.

Table 4. Differences of medical staff ’s Knowledge, Attitudes and Behavior of dysphagia assessment in Sociodemographic, training and working experience characteristics (n=353)

Characteristic		n(%)	KAB Mean±SD	95%CI	Univariate analysis (t/F, p)
**Hospital level**	Level 1	14 (4.0)	81.3±16.8	(71.6,91.0)	F=4.680, p=0.010
	Level 2	53 (15.0)	89.3±19.1	(84.0,94.5)	
	Level 3	286 (81.0)	93.8±17.0	(91.9,95.8)	
**Hospital type**	The general hospital	336 (95.2)	93.4±17.2	(91.6,95.2)	t=3.585, p<0.001
	Other hospital	17 (4.8)	78.0±18.2	(68.7,87.4)	
**Department**	Department (Neurology, Rehabilitation, Geriatrics)	177 (50.1)	100.6±13.6	(98.6,102.6)	t=9.566, p<0.001
	Other department	176 (49.9)	84.7±17.5	(82.1,87.3)	
**Position**	Clinical nurse	234 (66.3)	93.6±17.0	(91.5,95.8)	F=1.126, p=0.344
	Clinical doctor	68 (19.3)	91.1±18.7	(86.6,95.7)	
	Management personnel	34 (9.6)	92.5±16.9	(86.6,98.4)	
	Community nurses	12 (3.4)	87.3±22.7	(72.9,101.7)	
	Others	5 (1.4)	81.3±13.3	(64.8,97.7)	
**Title**	Primary title	165 (46.7)	92.2±17.6	(89.5,94.9)	F=0.342, p=0.710
	Medium-grade professional title	148 (41.9)	92.7±17.6	(89.8,95.5)	
	Senior title of professional	40 (11.3)	94.7±17.2	(89.2,100.2)	
**Working years in the field of**	None	113 (32.0)	82.5±17.0	(79.3,85.6)	F=24.700, p<0.001
	<3 years	63 (17.9)	93.2±17.6	(88.7,97.6)	

**dysphagia related diseases**	3-5 years	54 (15.3)	98.1±15.8	(93.8,102.4)	
	≥5 years	123 (34.8)	99.4±14.2	(96.9,101.9)	
**Education**	Junior college and below	62 (17.5)	89.7±19.4	(84.8,94.6)	F=1.148, p=0.319
	Bachelor	253 (71.7)	93.4±17.0	(91.3,95.5)	
	Master degree or above	38 (10.8)	92.2±17.8	(86.4,98.1)	
**Experience in nursing patients with dysphagia**	Yes	234 (66.3)	97.8±15.1	(95.8,99.7)	t=8.019, p<0.001
	No	119 (33.7)	82.6±17.7	(79.4,85.8)	
**Related training for dysphagia**	Yes	175 (49.6)	99.9±14.6	(97.7,102.0)	t=8.397, p<0.001
	No	178 (50.4)	85.6±17.3	(83.0,88.1)	
**Specialized training (geriatric, swallowing and rehabilitation)**	Yes	55 (15.6)	106.2±12.3	(102.9,109.5)	t=8.285, p<0.001
	No	298 (84.4)	90.2±17.2	(88.2,92.1)	

Note.Abbreviation: CI=confidence interval, KAB=Knowledge, Attitudes and Behavior.

Predictors of medical staff's KAB of dysphagia assessment

In the final regression analysis, all the categorical variables were transformed into dummy variables showed in S2 Table. Dummy multiple regression analysis was conducted to identify relevant predictors for medical staff's KAB in dysphagia assessment. As seen in Table 5, working years in the field of dysphagia related diseases, specialized training (geriatric, swallowing and rehabilitation); experience in nursing patients with dysphagia; Department (Neurology, Rehabilitation, Geriatrics) and related training for dysphagia were significant predictors of medical staff's KAB of dysphagia assessment, accounting for 35.1% of variance.

Table 5. Factors related to Knowledge, Attitudes and Behavior by stepwise regression

Variables	Std. β	t	p value
**Hospital level (ref: level 2): level 1**	-0.026	-0.579	0.563
**Hospital level (ref: level 2): level 3**	0.045	1.025	0.306
**Hospital type (ref: other hospital): the general hospital**	0.065	1.470	0.142
**Working years in the field of dysphagia related diseases (ref: <3 years): none**	-0.130	-2.530	0.012
**Working years in the field of dysphagia related diseases (ref: <3 years): 3-5 years**	0.020	0.449	0.654
**Working years in the field of dysphagia related diseases (ref: <3 years): ≥5 years**	0.023	0.454	0.650
**Specialized training (geriatric, swallowing and rehabilitation) (ref:**	0.148	3.171	0.002

**No): Yes**			
**Experience in nursing patients with dysphagia(ref: No): Yes**	0.172	3.491	0.001
**Department (Neurology, Rehabilitation, Geriatrics) (ref: No): Yes**	0.239	4.631	<0.001
**Related training for dysphagia(ref: No): Yes**	0.196	4.026	<0.001

note. Std. β, standardized β coefficient

Adjusted R^2=0.351, F=39.130, p<0.001

Discussion

Status of KAB of dysphagia assessment

The knowledge of medical staff on dysphagia assessment directly affects the quality of care and the prognosis of patients. However, most nurses lack the knowledge of dysphagia assessment, which may be related to lack of relevant training and attention paid by medical institutions to the knowledge education of nurses[18]. To build an appropriate and effective training program, it is necessary to combine the influencing factors of nurses' knowledge, attitude and behavior in the assessment of swallowing disorders. There is no research on the influencing factors of knowledge, attitude and behavior of dysphagia assessment in China. This study was conducted in multiple regions (4 provinces), multiple populations (doctors, clinical and community nurses, technicians, etc.) and multiple departments (neurosurgery and general departments) aiming to find as different data and features as possible to serving the training program.

In the study, the KAB score were 92.7±17.5, and the percentile rating score was 71.6. The percentile rank was 54.0, which means that 54.0% of participants' KAB scores were above the average score. According to the percentile rating score in Table 2, Knowledge and Behavior scores were medium, and attitude scores were high. This showed that the attitude score of medical staff is higher than the behavior score because the standardized score of attitude is 89.7, which is much higher than the standardized score of behavior 63.7.

The results of this study are different from study of Sun Qian et al[19]. The percentige rating score of knowledge and attitude in this study is similar to study of Sun Qian, which is at the medium and high level respectively. However, the percentige rating score of behavior in this study is slightly lower than study of Sun Qian (63.7 VS 71.0). The reasons may be as follows: Firstly, different participants and locations were surveyed in two studies. Sun Qian surveyed nurses in 17 Grade III hospitals in Beijing, while this study surveyed medical staff in hospitals of different levels in four provinces. Secondly, different departments were surveyed in two studies. Sun Qian only investigated the department of neurosurgery, which is known to pay more attention to the evaluation practice of swallowing disorders than other departments..

The lack of knowledge in this study was reflected in the following aspects: First, 80.4% and 76.3% participants did not know how to perform the volume-viscosity swallowing test (VVST) and water swallow test, respectively. This is related to the fact that 84.4% of the participants did not have Specialized training (geriatric, swallowing and rehabilitation) . Second, nearly half of the participants did not know the concept of mouthful size, food requirements for patients with dysphagia, or how to feed patients with hemiplegia. The deficiencies also included using the wrong utensils, incorrect positioning of the patient during feeding, or giving patients fluids or food of the wrong consistency. There's a study demonstrated that Speech-language pathologists varied in their views on the extent of their role in managing mealtime difficulties. Additionally, their self-rated knowledge of mealtime difficulties was lower than their dysphagia knowledge[20]. These indicated that the clinical attention to managing mealtime difficulties with dysphagia is insufficient. A study demonstrated that knowledge deficit was an important barrier in dysphagia care for nurses, and this can be improved with a short training[21]. Further research to develop efficient and effective training for care staff supporting mealtime difficulties and dysphagia is essential .

The lack of behavior in the study was mainly reflected in the following aspects. First, in 79.1% of the participants' departments, when stroke patients were screened for dysphagia, no further instrumental examinations were performed. Second, in 13.9% of the participants' departments, swallowing function was not screened in stroke patients after admission. Nurse stress with patient characteristics and workload may affect whether a swallow screen is undertaken[22]. The time of screening or assessment of OD prevalence was recorded. OD prevalence estimates from the hospital setting were reported as the time post-stroke or time from admission. Time post-stroke ranged from the hyperacute phase[23], ≤24h post-stroke, to the acute phase[24]; 1-7 days, to the early subacute phase[25];7 days-3 months, to the recovery phase[26]. Therefore, we should screen for or assess dysphagia at the right time based on the patient's condition and identify reliable and standardized assessment methods for screening post-stroke dysphagia(PSD) in acute stroke patients to ensure reliable estimates [27].

One study reported that 35.8% of professionals did not know the definition of dysphagia as a swallowing disorder[28]. Lack of knowledge among healthcare professionals can lead to inappropriate practices and increase the complications of dysphagia, such as aspiration pneumonia and malnutrition. This is an important barrier in the management of patients with dysphagia. One study investigated the Knowledge, Attitude and Practice of healthcare providers in Iran, and the results showed that very few participants were familiar with a standard test for screening and assessment of dysphagia (11.9%). A total of 74.7% were willing to participate in a workshop on dysphagia; the main pitfalls in their country lie in practice [29]. Another study reported that there seems to be limited awareness among ICU practitioners that patients are at risk of dysphagia, particularly as ventilation persists, protocols, routine assessment, and instrumental assessments are generally not used[30]. Therefore, it is important to improve the practice of dysphagia assessment.

Comparison of medical staff's KAB in different Provinces

In the study, the KAB of participants in Shaanxi Province was higher than that of participants in other provinces. The reasons for this are as follows. First, 95.0% of the participants in Shaanxi Province were from neurological, rehabilitation, and elderly related departments that mainly treat patients with stroke. There are many situations in the workplace that require the assessment of swallowing disorders. Second, 87.5% of participants in Shaanxi Province had experience in nursing patients with dysphagia.

Developing training resources by predictors of KAB of dysphagia assessment

There is still plenty of room to improve medical staff's Knowledge, Attitudes and Behavior regarding dysphagia assessment. Multiple linear regression results indicated that experience in nursing patients with dysphagia, related training for dysphagia, working years in the field of dysphagia related diseases, specialized training (geriatric, swallowing and rehabilitation) and department (Neurology, Rehabilitation, Geriatrics) were significant predictors. Consequently, the following countermeasures were proposed. The following countermeasures were based on the theory of Knowledge-Attitude-Practice and Self-efficacy.

Strengthening relevant training to improve Knowledge of dysphagia assessment

Knowledge-attitude-practice (KAP) theory is widely used in behavioral psychology[31]. This theory emphasizes that change in an individual's behavior consists of three continuous processes: acquiring knowledge, establishing beliefs and producing behavior, and paying attention to their causality and progressive relationship. The diagnosis and management of dysphagia requires comprehensive knowledge of diverse etiologies, with a systematic approach for the assessment of symptoms, selection of investigations, and appropriate treatment to relieve symptoms[32]. Meanwhile, staff who provide mealtime assistance to people with dysphagia require adequate training to ensure that mealtimes are safe and enjoyable[33]. In this study, knowledge consisted of an assessment approach, dysphagia symptoms, mealtime assistance and so on. The mean score of medical staff was 15.3±2.7, which was at the middle level, and 49.6% participants had received related training for dysphagia(35.8% in other departments VS 63.3% in Neurology, Rehabilitation or Geriatrics department). Only 15.6% of the participants had received Specialized training (geriatric, swallowing and rehabilitation) (8.5% in other departments VS 22.6% in Neurology, Rehabilitation or Geriatrics department). Medical staff in Neurology, Rehabilitation or Geriatrics department seldom receive related training for dysphagia, which leads to less knowledge of assessment or screening for dysphagia. Therefore, it is more difficult for medical staff to establish positive beliefs regarding dysphagia assessment and screening. To standardize the management of dysphagia,

hospitals should take the rate of dysphagia assessment as a quality control index and promote training in dysphagia assessment. Examples include the Volume-Viscosity Swallow Test(V-VST), water swallow test, eating assessment Tool-10(EAT-10), Mann Assessment of Swallowing Ability (MASA) and so on.

Establishing positive belief in assessment of dysphagia

According to KAP theory, beliefs and attitudes are the motive forces for individuals to produce related behaviors[31]. As the results of study, the attitude score of medical staff was 35.9±4.9, which was at the high level. This finding suggested that many people are willing to screen and evaluate patients with dysphagia. This was a good trend. Nurses gained a sense of value by assessing and caring for patients with dysphagia. Dysphagia screening is often the focus of hospitalized stroke patients; however, dysphagia can also occur in other hospitalized patients and outpatients. Dysphagia can be overlooked by nurses and clinicians, and it is important to educate nurses on the importance of dysphagia screening [34].

Accumulate dysphagia assessment experience through work and study

Self-efficacy refers to an individual's conviction of their capacity to perform a specific activity. Individuals gain self-efficacy over time as they acquire a range of talents, such as social, cognitive, physical, and linguistic abilities, via life experiences[35]. Efficacy expectations are dynamic and are both appraised and enhanced by four mechanisms[36-37]: (1) enactive mastery experience or successful performance of the activity of interest; (2) verbal persuasion or encouragement, given by a credible source that the individual is capable of performing the activity of interest; (3) vicarious experience or seeing like individuals perform a specific activity; and (4) physiological and affective states such as pain, fatigue, anxiety, hunger, or dizziness associated with a given activity. These mechanisms to drive efficacy expectations are the concepts upon which the intervention is built to encourage behavioral change. In this study, experience in nursing patients with dysphagia, working years in the field of dysphagia related diseases, and department (Neurology, Rehabilitation Geriatrics) were significant predictors of medical staff's Knowledge, Attitudes and Behavior of dysphagia assessment. The results are consistent with the self-efficacy theory. This suggested that if conditions permit, the medical staff in other departments with no experience of dysphagia patients' nursing should transfer to Neurology, Rehabilitation or Geriatrics department to be trained and accumulate relevant experience. Alternatively, the hospital organizes regular workshops for dysphagia assessment and nurses are required to participate in practice.

Establish systematic inter-professional collaboration in dysphagia management

A study showed that the main theme "limited professional services" describes how

patients received little support from healthcare professionals and had to rely on themselves to adapt to life with dysphagia[38]. The common goal of preventing aspiration and rehabilitating patients' ability to swallow safety is based on dysphagia assessment, using appropriate therapeutic interventions, sharing knowledge, and improving skills among professional groups that consist of nurses, physicians, occupational therapists, and speech-language pathologists(SLPs)[39].

The strengths and limitations of this study

First, participants in this study were from four provinces in China, showing the differences in knowledge, attitude and behavior of medical staff in different regions in dysphagia assessment. Second, this study explored the influencing factors of medical staff's knowledge, attitude and behavior in tdysphagia assessment, and provided guidance and suggestions for the future organization of relevant training. But, this study only investigated hospital and community nurses, and did not investigate healthcare professionals in other different Settings such as nursing home settings. Future studies should focus on the evaluation of dysphagia and provide training for the assessment of swallowing disorders to medical staff in nursing home settings, social health service centers, and palliative care facilities.

Conclusions

The attitude of Chinese medical staff to dysphagia assessment is positive, but the knowledge and behavior needs to be improved, and the attitude is separated from the knowledge and the behavior.This study findings also implied that nursing experience, training, and work for patients with swallowing disorders could have positive effects on the Knowledge, Attitudes and Behavior of medical staff regarding dysphagia assessment. The medical management department should take various forms to train the knowledge and skills related to the evaluation of swallowing disorders. Hospital administrators should provide relevant resources, such as videos of dysphagia assessment, training centers for the assessment of dysphagia, and swallowing specialist nurses. It is important that health policies fully recognize the role of training and support systems in caring for people with dysphagia..

Conflict of interest statement

The authors declare that there are no conflict of interests, we do not have any possible conflicts of interest.

Data access statement

The data was deposited at Dryad (DOI: https://doi.org/10.5061/dryad.djh9w0w70)..

Supporting information

S1 Table. The scoring methods and rules of the questionnaire. (DOC)

Table 1 The scoring methods and rules of the questionnaire

Domains	Items	n	scoring methods and rules
Knowledge	True or False	17	1 point is scored for correct answer. 0 point

	questions		is scored for incorrect answer.
	Single-choice questions	4	
	Multiple-choice questions	4	Count 0.2/ option when selecting the correct option. Count 0 when the wrong option was selected or the correct and wrong options were selected simultaneously<br>For example, a multiple-choice question had three correct choices that score 0.6 when participant selected three correct choices.
Attitudes	Likert-five-point-scale	8	Likert 5-level scoring method. Ranging from “strongly agreed” to “strongly disagreed”. 5 point for “strongly agreed”, 1 point for “strongly disagreed”
Behavior	Likert-five-point-scale	13	Likert 5-level scoring method. Ranging from “the most frequently” to “the least frequently. 5point for “the most frequently”, 1 point for “the least frequently

S2 Table. Dummy variable settings. (DOC)

Acknowledgements

The authors would like to thank all participated medical staff who took the time to complete survey and made this study possible.

Author contributions

Conceptualization, Statistical analysis, Writing-review & editing: Juanhui Chen
Investigation: Juanhui Chen, Wenqiu Ye, Xingyun Zheng, Wenna Wu, Yuebao Chen, Yinjuan Chen

References

[1] GBD 2019 Stroke Collaborators. Global, regional, and national burden of stroke and its risk factors, 1990-2019: a systematic analysis for the Global Burden of Disease Study 2019. Lancet Neurol. 2021;20(10):795-820. doi:10.1016/S1474-4422(21)00252-0.

[2] Vilardell N, Rofes L, Nascimento WV, Muriana D, Palomeras E, Clavé P. Cough reflex attenuation and swallowing dysfunction in sub-acute post-stroke patients: prevalence, risk factors, and clinical outcome. Neurogastroenterol Motil. 2017 Jan;29(1). doi: 10.1111/nmo.12910. Epub 2016 Jul 18. PMID: 27424849.

[3] Matsumura T, Mitani Y, Oki Y, Fujimoto Y, Ishikawa A. Risk factors for the onset of aspiration pneumonia among stroke patients in the recovery stage. Nihon Ronen Igakkai Zasshi. 2014;51(4):364-8.doi: 10.3143/geriatrics.51.364. PMID: 25327371.

[4] Finnegan BS, Meighan MM, Warren NC, Hatfield MK, Alexeeff S, Lipiz J, et al. Validation Study of Kaiser Permanente Bedside Dysphagia Screening Tool in Acute

Stroke Patients. Perm J. 2020 Dec;24:1. doi: 10.7812/TPP/19.230. PMID: 33482958; PMCID: PMC7849282.
[5] Baijens LW, Clavé P, Cras P, Ekberg O, Forster A, Kolb GF, et al. European society for swallowing disorders-European union geriatric medicine society white paper: oropharyngeal dysphagia as a geriatric syndrome. Clin Interv Aging. 2016 Oct 7;11:1403-1428. doi: 10.2147/CIA.S107750. PMID: 27785002; PMCID: PMC5063605.
[6] Rivelsrud MC, Hartelius L, Bergström L, Løvstad M, Speyer R. Prevalence of Oropharyngeal Dysphagia in Adults in Different Healthcare Settings: A Systematic Review and Meta-analyses. Dysphagia. 2023 Feb;38(1):76-121. doi: 10.1007/s00455-022-10465-x. Epub 2022 May 31. PMID: 35639156; PMCID: PMC9873728.
[7] Blanař V, Hödl M, Lohrmann C, Amir Y, Eglseer D. Dysphagia and factors associated with malnutrition risk: a 5-year multicentre study. J Adv Nurs.2019;75(12):3566–76.doi: 10.1111/jan.14188. Epub 2019 Sep 10. PMID: 31452231.
[8] Huppertz VAL, Halfens RJG, van Helvoort A, de Groot LCPGM, Baijens LWJ, Schols JMGA. Association between Oropharyngeal Dysphagia and Malnutrition in Dutch Nursing Home Residents: Results of the National Prevalence Measurement of Quality of Care. J Nutr Health Aging. 2018;22(10):1246-1252. doi: 10.1007/s12603-018-1103-8. PMID: 30498833; PMCID: PMC6302767.
[9] Beharry A, Michel P, Faouzi M, Kuntzer T, Schweizer V, Diserens K. Predictive Factors of Swallowing Disorders and Bronchopneumonia in Acute Ischemic Stroke. J Stroke Cerebrovasc Dis. 2019 Aug;28(8):2148-2154. doi: 10.1016/j.jstrokecerebrovasdis.2019.04.025. Epub 2019 May 22. PMID: 31129105.
[10] Speyer R, Cordier R, Farneti D, Nascimento W, Pilz W, Verin E, et al. White Paper by the European Society for Swallowing Disorders: Screening and Non-instrumental Assessment for Dysphagia in Adults. Dysphagia. 2022 Apr;37(2):333-349. doi: 10.1007/s00455-021-10283-7. Epub 2021 Mar 31. PMID: 33787994; PMCID: PMC8009935.
[11] Wilkinson JM, Codipilly DC, Wilfahrt RP. Dysphagia: Evaluation and Collaborative Management. Am Fam Physician. 2021 Jan 15;103(2):97-106. PMID: 33448766.
[12] Dumican M, Thijs Z, Harper K. Clinical practice patterns of speech-language pathologists for screening and identifying dysphagia. Int J Lang Commun Disord. 2023 Nov-Dec;58(6):2062-2076. doi: 10.1111/1460-6984.12921. Epub 2023 Jun 27. PMID: 37376825.
[13] Tan L, Gan G, Hum A, Lee A. A Stepwise, Mixed-Method Study Approach to Identify the Barriers to Dysphagia Care in Hospice Care Nurses. J Hosp Palliat Nurs. 2018 Feb;20(1):88-94. doi: 10.1097/NJH.0000000000000404. PMID: 30063619.
[14] Zhang Y, Liu Y, Li W, Lin Y. Investigation on the knowledge and intervention status of swallowing function training in elderly patients by nurses in Guangzhou and Shenzhen. Journal of Nurses Training, 2018,33(16):1499-1500,1531.DOI:10.16821/j.cnki.hsjx.2018.16.021.

[15] Kendall MG. kendall's advanced theory of statistics. Halsted Press; Edward Arnold, 2004.
[16] Dong X. Evidence-based management of stroke patients with dysphagia and its effect evaluation. Zhengzhou University,2018.
[17] Ma K, GUO Y, DONG X, Yang C, Zhang P, Wang M, et al. Investigation on the status of screening and evaluation of dysphagia in stroke patients by nurses in Henan Province. Chinese journal of nursing,2020,55(08):1196-1200. DOI:10.3761/j.issn.0254-1769.2020.08.016.
[18] Lv S, Zhou G, Mao Y, Zhang Y, Wang J. Research progress on the attitude, knowledge and behavior of nurses and caregivers for swallowing disorders after stroke in China. Chinese General practice nursing,2023,21(1):37-41. DOI:10.12104/j.issn.1674-4748.2023.01.009.
[19] Sun Q, Su Y, Wang Y.Investigation on the current situation of knowledge and practice in nursing of dysphagia after stroke in neurology nurses. Chinese journal of modern nursing,2020,26(23):3144-3150. DOI:10.3760/cma.j.cn115682-20200322-02063.
[20] Egan A, Andrews C, Lowit A. Dysphagia and mealtime difficulties in dementia: Speech and language therapists' practices and perspectives. Int J Lang Commun Disord. 2020 Sep;55(5):777-792. doi: 10.1111/1460-6984.12563. Epub 2020 Jul 24. PMID: 32706516.
[21] Tan L, Gan G, Hum A, Lee A. A Stepwise, Mixed-Method Study Approach to Identify the Barriers to Dysphagia Care in Hospice Care Nurses. J Hosp Palliat Nurs. 2018 Feb;20(1):88-94. doi: 10.1097/NJH.0000000000000404. PMID: 30063619.
[22] Moghadam KN, Chehrzad MM, Masouleh SR, Mardani A, Maleki M, Akhlaghi E, et al. Nursing workload in intensive care units and the influence of patient and nurse characteristics. Nurs Crit Care. 2021 Nov;26(6):425-431. doi: 10.1111/nicc.12548. Epub 2020 Sep 20. PMID: 32954619.
[23] Bernhardt J, Hayward KS, Kwakkel G, Ward NS, Wolf SL, Borschmann K, et al. Agreed definitions and a shared vision for new standards in stroke recovery research: The Stroke Recovery and Rehabilitation Roundtable taskforce. Int J Stroke. 2017 Jul;12(5):444-450. doi: 10.1177/1747493017711816. PMID: 28697708.
[24] Rajashekar D, Mouchès P, Fiehler J, Menon BK, Goyal M, Demchuk AM, et al. Structural integrity of white matter tracts as a predictor of acute ischemic stroke outcome. Int J Stroke. 2020 Dec;15(9):965-972. doi: 10.1177/1747493020915251. Epub 2020 Mar 31. PMID: 32233745.
[25] Abubakar SA, Jamoh BY. Dysphagia following acute stroke and its effect on short-term outcome. Niger Postgrad Med J.2017;24(3):182–6.doi: 10.4103/npmj.npmj_96_17. PMID: 29082909.
[26] Paik NJ, Kim WS. Recovery of Swallowing. 2021 Jan 15. In: Platz T, editor. Clinical Pathways in Stroke Rehabilitation: Evidence-based Clinical Practice Recommendations [Internet]. Cham (CH): Springer; 2021. PMID: 36315693..
[27] Banda KJ, Chu H, Kang XL, Liu D, Pien LC, Jen HJ, et al. Prevalence of dysphagia and risk of pneumonia and mortality in acute stroke patients: a meta-analysis. BMC Geriatr. 2022 May 13;22(1):420. doi:

10.1186/s12877-022-02960-5. PMID: 35562660; PMCID: PMC9103417..
[28] Sánchez-Sánchez E, Avellaneda-López Y, García-Marín E, Ramírez-Vargas G, Díaz-Jimenez J, Ordonez FJ. Knowledge and Practice of Health Professionals in the Management of Dysphagia. Int J Environ Res Public Health. 2021 Feb 22;18(4):2139. doi: 10.3390/ijerph18042139. PMID: 33671732; PMCID: PMC7926391..
[29] Farpour S, Farpour HR, Smithard D, Kardeh B, Ghazaei F, Zafarghasempour M. Dysphagia Management in Iran: Knowledge, Attitude and Practice of Healthcare Providers. Dysphagia. 2019 Feb;34(1):105-111. doi: 10.1007/s00455-018-9919-2. Epub 2018 Jun 21. PMID: 29931606.
[30] Spronk PE, Spronk LEJ, Egerod I, McGaughey J, McRae J, Rose L, et al. Dysphagia in Intensive Care Evaluation (DICE): An International Cross-Sectional Survey. Dysphagia. 2022 Dec;37(6):1451-1460. doi: 10.1007/s00455-021-10389-y. Epub 2022 Jan 29. PMID: 35092486.
[31] Liu C, Liu C, Wang D, Zhang X. Knowledge, Attitudes and Intentions to Prescribe Antibiotics: A Structural Equation Modeling Study of Primary Care Institutions in Hubei, China. Int J Environ Res Public Health. 2019 Jul 5;16(13):2385. doi: 10.3390/ijerph16132385. PMID: 31284381; PMCID: PMC6651188.
[32] Nigam GB, Vasant DH, Dhar A. Curriculum review : investigation and management of dysphagia. Frontline Gastroenterol. 2021 Aug 3;13(3):254-261. doi: 10.1136/flgastro-2021-101917. PMID: 35493628; PMCID: PMC8996094.
[33] Reddacliff C, Hemsley B, Smith R, Dalton S, Jones S, Fitzpatrick A, et al. Examining the Content and Outcomes of Training in Dysphagia and Mealtime Management: A Systematic Review Informing Co-Design of New Training. Am J Speech Lang Pathol. 2022 May 10;31(3):1535-1552. doi: 10.1044/2022_AJSLP-21-00231. Epub 2022 Apr 4. PMID: 35377733.
[34] West J, Stutzman SE, Atem F, Olson DM. Formatting an experiential learning education module to encourage dysphagia assessment in apheresis patients. J Clin Apher. 2018 Feb;33(1):72-77. doi: 10.1002/jca.21572. Epub 2017 Aug 2. PMID: 28766740.
[35] Huo C, Hameed J, Nawaz A, Adnan Raheel Shah S, Albahser G, Alqahtani W, Maqsoom A, Kashif Anwar M. Scientific risk performance analysis and development of disaster management framework: A case study of developing Asian countries. J King Saud Univ Sci. 2021 Mar;33(2):101348. doi: 10.1016/j.jksus.2021.101348. Epub 2021 Jan 28. PMID: 35495615; PMCID: PMC9042590.
[36] Bandura A. Self-Efficacy: The Exercise of Control. New York: WH Freeman; 1997.
[37] Resnick B. Geriatric rehabilitation: the influence of efficacy beliefs and motivation. Rehabil Nurs. 2002;27(4):152–159.doi: 10.1002/j.2048-7940.2002.tb02224.x. PMID: 12116528.
[38] Li C, Qiu C, Shi Y, Yang T, Shao X, Zheng D. Experiences and perceptions of stroke patients living with dysphagia: A qualitative meta-synthesis. J Clin Nurs. 2022 Apr;31(7-8):820-831. doi: 10.1111/jocn.15995. Epub 2021 Aug 8. PMID: 34369025.
[39] Nielsen AH, Kaldan G, Nielsen BH, Kristensen GJ, Shiv L, Egerod I. Intensive care professionals' perspectives on dysphagia management: A focus group study. Aust

Crit Care. 2023 Jul;36(4):528-535. doi: 10.1016/j.aucc.2022.04.004. Epub 2022 May 21. PMID: 35610091.

Click here to access/download
Supporting Information
S1 Table The scoring methods and rules of the questionnaire.doc

Click here to access/download
Supporting Information
S2 Table Dummy variable settings.doc

Predictors of medical staff's Knowledge, Attitudes and Behavior of dysphagia assessment: A Cross-Sectional study

Juanhui Chen[1*], Wenqiu Ye[2], Xingyun Zheng[3], Wenna Wu[4], Yuebao Chen[5], Yinjuan Chen[6]

1 Department of Nursing, Longgang Central Hospital of Shenzhen, Shenzhen, Guangdong province, China. 2 Department of Neurology, Longgang Central Hospital of Shenzhen, Shenzhen, Guangdong province, China. 3 Department of Neurosurgery, Longgang Central Hospital of Shenzhen, Shenzhen, Guangdong province, China. 4 Department of Rehabilitation, Longgang Central Hospital of Shenzhen, Shenzhen, Guangdong province, China. 5 Department of Neurology, Guangxi International Zhuang Medicine hospital, Nanning, Guangxi province, China. 6 Department of Nursing, Shenzhen FuYong People's Hospital, Shenzhen, Guangdong province, China.

Running head: Predictors of medical staff' s KAP of dysphagia assessment

***Correspondence**

E-mail:pugongyingchenjuan@163.com (JC)

Abstract

Purpose: This study aimed to explore the predictors of medical staff' s Knowledge, Attitudes and Behavior of dysphagia assessment, to provide reference suggestions for constructing the training program and improving the rate of dysphagia assessment.

Methods: This study was a cross-sectional study. A total of 353 nurses and doctors from four provinces (Guangdong, Hunan, Guangxi, and Shaanxi) who were selected by convenience sampling and invited to complete the questionnaire through WeChat, DingTalk, and Tencent instant messenger from May 23 to 31, 2022. A self-reported questionnaire was used to assess participants' Knowledge, Attitude and Behavior regarding dysphagia assessment. Participants' sociodemographic, training, and nursing experience were measured using the general information sheet and analyzed as potential predictors of medical staff's Knowledge, Attitudes and Behavior of dysphagia assessment. A multiple linear regression model was used to identify the predictors.

Results: The mean score for Knowledge, Attitudes and Behavior of dysphagia assessments was (92.7±17.5). Multiple linear regression results indicated that experience in nursing patients with dysphagia, related training for dysphagia, working years in the field of dysphagia related diseases, specialized training (geriatric, swallowing and rehabilitation) and department (Neurology, Rehabilitation, Geriatrics) were significant predictors, accounting for 35.1% of the variance.

Conclusions: The study findings implied that nursing experience, training, and work for patients with swallowing disorders could have positive effects on the

设置格式[蒲]: 字体: 非倾斜
删除[蒲]: and
删除[蒲]:
删除[蒲]:
删除[蒲]: P
删除[蒲]: redictors of medical staff' s Knowledge, Attitu
删除[蒲]: H
删除[蒲]: **Project:**
设置格式[蒲]: 字体: 加粗, 非倾斜
设置格式[蒲]: 字体: 非倾斜
删除[蒲]: Unfunded project of Medical and Health
设置格式[蒲]: 字体: 三号, 非倾斜
设置格式[蒲]: 字体: 非倾斜
删除[蒲]: his study aimed to develop training resources a
设置格式[蒲]: 字体: 三号, 非倾斜
设置格式[蒲]: 字体: 非倾斜
删除[蒲]: 430
删除[蒲]: Province
删除[蒲]: Province
删除[蒲]: Province
删除[蒲]: Province
删除[蒲]: were
删除[蒲]: assess
删除[蒲]: factors
删除[蒲]: ing
删除[蒲]: medical staff's Knowledge, Attitudes and
设置格式[蒲]: 字体: 三号, 非倾斜
设置格式[蒲]: 字体: 非倾斜
删除[蒲]: s
删除[蒲]: were
删除[蒲]: 6
删除[蒲]: 54
删除[蒲]:
删除[蒲]: 19

Knowledge, Attitudes and Behavior of medical staff regarding dysphagia assessment. Hospital administrators should provide relevant resources, such as videos of dysphagia assessment, training centers for the assessment of dysphagia, and swallowing specialist nurses. It is important that health policies fully recognize the role of training and support systems in caring for people with dysphagia.

Abbreviations: KAB=Knowledge, Attitudes and Behavior, MS-KAB-DA=Questionnaire of Medical Staff's Knowledge, Attitudes and Behavior of dysphagia assessment.

Key words: assessment, Attitude, Behavior, Cross-Sectional, dysphagia, Knowledge, Predictors, stroke

Introduction

Worldwide, there are approximately 80.1 million stroke survivors, with an estimated 13.7 million new cases of stroke every year. Stroke remained the second-leading cause of death and the third-leading cause of death and disability combined[1]. Dysphagia occurs in 30.0–78.0% of patients after stroke[2-4]. Dysphagia may be defined as the partial or complete inability to prepare and move a bolus of food, fluids, or saliva efficiently and safely from the mouth to the esophagus and stomach[5]. Oropharyngeal Dysphagia(OD) is prevalent in the elderly and people with complex medical conditions; for example, aspiration pneumonia is caused by silent aspiration in stroke patients, resulting in considerable medical psychosocial consequences, reduced quality of life, and affected prognosis of patients. Prevalence estimates for OD determined by a meta-analysis were 36.5% in hospital settings, 42.5% in rehabilitation settings, and 50.2% in nursing homes[6].

The type and combination of screening and assessment methods used to determine the prevalence of OD varies. More than half of the studies reported OD prevalence data using screening and clinical non-instrumental assessment methods or tools that were either designed by the authors for the purpose of the study or modified versions of published tools. Thus lacking information on diagnostic performance and psychometric properties[7-9]. However, instrumental assessments require specialized training and equipment because of feasibility (e.g., availability, ease of administration). Screening and clinical non-instrumental assessments are the natural first choice for estimating the prevalence of OD[10]. Speech-language pathologists and other specialists, in collaboration with family physicians, can provide structured assessments and make appropriate recommendations for safe swallowing[11]. One study's results suggested that continuing education hours were significantly associated with dysphagia screening protocol choice[12]. One study evaluated nurses' barriers to compliance with dysphagia measures. And it demonstrated that a knowledge deficit was an important barrier in dysphagia care for nurses, and this can be improved with short training[13].

Studies on the factors contributing to barriers to the assessment of dysphagia are lacking. Some Chinese researchers investigated the practice and management of

设置格式[蒲]: 字体: 三号, 非倾斜

设置格式[蒲]: 字体: 非倾斜

删除[蒲]: s

设置格式[蒲]: 字体: 三号, 非倾斜

设置格式[蒲]: 字体: 非倾斜

删除[蒲]: d

删除[蒲]: ysphagia, assessment, stroke, Knowledge, Attitude, Behavior, Cross-Sectional, Predictors

删除[蒲]: 7

删除[蒲]: s

删除[蒲]: the

设置格式[蒲]: 缩进: 首行缩进: 1 字符

删除[蒲]: ,

删除[蒲]: t

删除[蒲]: The study

medical staff's dysphagia assessment, it showed that More than 96.0% of the nurses did not know anything about the swallowing function training, and only 36.7% of the nurses used the scale to screen the patients for swallowing disorders[14]. And these studies did not explore the influencing factors. We aimed to explore factors contributing to barriers to the assessment of dysphagia, to provide reference suggestions for constructing the training program and improving the rate of dysphagia assessment by conducting this survey.

Methods

Design and Participants

This was a cross-sectional study. A total of 430 nurses and doctors were selected by convenience sampling from four provinces (Guangdong, Hunan, Guangxi, and Shaanxi). The inclusion criteria were as follows: (a) Chinese nurse practitioners, doctors, or therapists with a medical background, (b) working at the hospital for more than 3 months, and (c) not internship.

According to the sample content estimation method proposed by Kendall, the sample content of questionnaire survey should be 5-10 times the number of items[15]. There were 46 items in the questionnaire. Thus, 230-460 participants were calculated according to the ratio. Adding a 15.0% attrition rate, the total participants sample should between 265 and 529. A total of 353 nurses and doctors completed the survey in this study.

An anonymous survey was conducted by Longgang central hospital of Shenzhen on May 23-31, 2022. Participants were informed about the aims and content of the study and the importance of enrollment. A total of 430 nurses and doctors completed the questionnaire, corresponding to a response rate of 100.0%. The valid questionnaire was 82.1%, and invalid questionnaires were eliminated. The criteria for eliminating invalid questionnaires were (a) incomplete questionnaires, (b) a questionnaire with incorrect content, (c) logically contradictory questionnaires, and (d) time to fill in the questionnaire is shorter than 300 seconds and longer than 3600 seconds.

Ethical statements

This study was approved by Ethics Committee of Longgang Central Hospital of Shenzhen (No. 2022ECPJ101). The first part of the questionnaire mainly included informed consent, participants will read the informed consent at first, if they agree to participate in the study they will chose “I agree” to complete the following survey. The questionnaire survey was anonymous and personal information was not disclosed.

Research instruments

The following instrument covered two parts:

a) The general information sheet. Demographic characteristics were measured using the general information sheet, which includes 11 items: province, hospital level and type, department, position, title, working years in the field of dysphagia-related

设置格式[蒲]: 字体: 非倾斜, 上标
删除[蒲]: 3a
设置格式[蒲]: 字体: 非倾斜
设置格式[蒲]: 字体: 非倾斜, 上标
设置格式[蒲]: 字体: 非倾斜
删除[蒲]: investigating the predictors of medical staff’s
删除[蒲]:
删除[蒲]: in
删除[蒲]: Province
删除[蒲]: Province
删除[蒲]: Province
删除[蒲]: Province
删除[蒲]: were selected by convenience sampling
删除[蒲]:
设置格式[蒲]: 缩进: 首行缩进: 1 字符
删除[蒲]: Based on the suggestion of Hair, Black, Babin,
删除[蒲]: 4
删除[蒲]: , a ratio of 10 to 20 participants for each param
删除[蒲]: 22
删除[蒲]: parameters
删除[蒲]: 2
删除[蒲]: 4
删除[蒲]: is
删除[蒲]: 53
删除[蒲]: 06
删除[蒲]: 220–440 participants were followed by a
删除[蒲]: 09
删除[蒲]: a questionnaire that completed less than 300 s
删除[蒲]: .
删除[蒲]:
设置格式[蒲]: 字体: 三号, 非倾斜
设置格式[蒲]: 字体: 非倾斜
设置格式[蒲]: 缩进: 首行缩进: 0 字符
删除[蒲]: The following instrument were used to measure

diseases, education, experience of nursing patients with dysphagia, related training for dysphagia, specialized training (geriatric, swallowing and rehabilitation).

b) **Questionnaire on medical staff's Knowledge, Attitudes and Behavior of dysphagia assessment**. This questionnaire was revised based on the existing questionnaires designed by Dr. Xiaofang Dong[16] and Master Keke Ma[17] from The First Affiliated Hospital of Zhengzhou University. Before the study, we obtained the consent of the author of the original questionnaire to add the scoring method and paraphrase the items of knowledge dimension on the basis of the original questionnaire. The KAB was shortened for Knowledge, Attitudes and Behavior. The MS-KAB-DA was shortened for the Questionnaire of Medical staff's Knowledge, Attitudes, and Behavior of dysphagia assessment. The questionnaire was composed of three specific domains, namely Knowledge (25 items), Attitude (8 items), and Behavior (13 items). The knowledge domain consisted of 17 True or False questions (KR-20=0.760), four single-choice questions, and four multiple-choice questions. The scoring methods and rules of the questionnaire were shown in S1 Table. The total full score of Knowledge, Attitude and Behavior were 24.4, 40 and 65, respectively. Higher scores indicated higher cognitive, willingness and practice levels. Internal consistency estimates were shown to have acceptable reliability for the questionnaire before the survey (Cronbach ' s alpha for domain K, A, and B are 0.77, 0.97 and 0.91, respectively).

The instrument was administered in Chinese language and designed as a google form, the website was https://www.wjx.cn/vm/mc5kOo1.aspx.

Data collection and Analysis

All participants were invited to complete the google form through WeChat, DingTalk, and Tencent instant messenger. A professional questionnaire survey platform which provides functions equivalent to Amazon Mechanical Turk called "Wenjuan Xing" was used to investigate. The researcher sent the google form to colleagues and classmates to fill in and asked them to forward to their colleagues. Data were analyzed using SPSS for Windows, version 23. Statistical significance was set at $p < 0.05$. The findings were summarized using descriptive statistics, univariate analysis, and dummy multiple regression analysis. The data were normally distributed by skewness and kurtosis tests. Descriptive statistics (e.g., mean ± SD, frequency & %) were used to summarize the study variables. Univariate analysis (independent-samples t-test and one-way analysis of variance for categorical independent variables) was performed to explore the potential predictors of medical staff KAB for dysphagia assessment. A comparison analysis was conducted to analyze the differences in medical staff's KAB for dysphagia assessment among four Provinces.

Results

Characteristics of the Participants

删除[蒲]: specialized training in geriatric, swallowing, an

设置格式[蒲]: 字体: 非倾斜, 上标

删除[蒲]: 5

设置格式[蒲]: 字体: 非倾斜

设置格式[蒲]: 字体: 非倾斜, 上标

设置格式[蒲]: 字体: 非倾斜

设置格式[蒲]: 字体: 非倾斜, 上标

删除[蒲]: 6

设置格式[蒲]: 字体: 非倾斜

设置格式[蒲]: 字体: 非倾斜, 上标

设置格式[蒲]: 字体: 非倾斜

删除[蒲]: I

删除[蒲]: This questionnaire was revised based on the

删除[蒲]: ,

删除[蒲]: which

删除[蒲]: i

删除[蒲]: :

删除[蒲]: Kk

删除[蒲]: Aa

删除[蒲]: eight

删除[蒲]: b

删除[蒲]: items

删除[蒲]: items

删除[蒲]: are

设置格式[蒲]: 缩进: 首行缩进: 1 字符

删除[蒲]: questionnaire

删除[蒲]: True or false items and single-choice items sco

设置格式[蒲]: 缩进: 首行缩进: 0 字符

删除[蒲]: This study was approved by the ethics committ

删除[蒲]: from May 23 to May 31, 2022

删除[蒲]: through WeChat, DingTalk, and Tencent QQ

删除[蒲]: the questionnaire

删除[蒲]: , as assessed

删除[蒲]: ,

A total of 430 nurses and doctors were enrolled, of which 353 nurses and doctors were valid. Clinical nurses, clinicians, and managers accounted for 66.3%, 19.3%, and 9.6%, respectively. Of the included hospitals, 81.0% were tertiary hospitals. 12 community nurses were all from Guangdong Province. 10 community nurses were from level 3 hospital, only 2 from level 1 hospital. 7 community nurses (58.3%) had no working experience in the field of dysphagia related diseases and also had no related training for dysphagia. Other characteristics, such as department, medical staff work, and learning experience are summarized in Table 1.

Table 1. Demographic characteristics of the study participants (n=353)

Participants' characteristics		Guangdong n (%)	Hunan n (%)	Shaanxi n (%)	Guangxi n (%)
Hospital level	Level 1	13 (6.2)	1 (1.3)	0 (0.0)	0 (0.00)
	Level 2	49 (23.2)	3 (3.4)	0 (0.0)	1 (4.0)
	Level 3	149 (70.6)	73 (95.3)	40 (100.0)	24 (96.0)
Hospital type	The general hospital	194 (91.9)	77 (100.0)	40 (100.0)	25(100.0)
	Other hospital	17 (8.1)	0 (0.0)	0 (0.0)	0 (0.0)
Department	Department (Neurology, Rehabilitation, Geriatrics)	80 (37.9)	39 (50.6)	38 (95.0)	20 (80.0)
	Other department	131 (62.1)	38 (49.4)	2 (5.0)	5 (20.0)
Position	Clinical nurse	131 (62.1)	51 (66.2)	38 (95.0)	14 (56.0)
	Clinical doctor	35 (16.6)	23 (29.9)	0 (0.0)	10 (40.0)
	Management personnel	30 (14.2)	2 (2.6)	1 (2.5)	1 (4.0)
	Community nurses	12 (5.7)	0 (0.0)	0 (0.0)	0 (0.0)
	Others	3 (1.4)	1 (1.3)	1 (2.5)	0 (0.0)
Title	Primary title	94 (44.6)	36 (46.8)	23 (57.5)	12 (48.0)
	Medium-grade professional title	91 (43.1)	30 (38.9)	17 (42.5)	10 (40.0)
	Senior title of professional	26 (12.3)	11 (14.3)	0 (0.0)	3 (12.0)
Working years in the field of dysphagia related diseases	None	87 (41.2)	13 (16.9)	7 (17.5)	6 (24.0)
	<3 years	32 (15.2)	24 (31.1)	5 (12.5)	2 (8.0)
	3-5 years	28 (13.3)	12 (15.6)	8 (20.0)	6 (24.0)
	≥5 years	64 (30.3)	28 (36.4)	20 (50.0)	11 (44.0)
Education	Junior college and below	43 (20.4)	18 (23.4)	0 (0.0)	1 (4.0)
	Bachelor	145 (68.7)	53 (68.8)	39 (97.5)	16 (64.0)
	Master degree or above	23 (10.9)	6 (7.8)	1 (2.5)	8 (32.0)
Experience in	Yes	137 (64.9)	44 (57.1)	35 (87.5)	18 (72.0)

删除[蒲]: 29
删除[蒲]: 26
删除[蒲]: 3
删除[蒲]: 2
删除[蒲]: ,
删除[蒲]: Total n(%)
设置格式[蒲]: 字体: （默认）Times New Roman, 加粗
带格式表格[蒲]
删除[蒲]: C
删除[蒲]: n=211
删除[蒲]: n=77
删除[蒲]: n=40
删除[蒲]: n=353
删除[蒲]: 1
设置格式[蒲]: 字体: （默认）Times New Roman, 非倾斜
删除[蒲]: hospital
删除[蒲]: 16
删除[蒲]: 0
删除[蒲]: 0
删除[蒲]: 14
删除[蒲]: 3.97
删除[蒲]: 1
设置格式[蒲]: 字体: （默认）Times New Roman, 非倾斜
删除[蒲]: hospital
删除[蒲]: 2
删除[蒲]: 0
删除[蒲]: 0
删除[蒲]: 53
删除[蒲]: 15.01
删除[蒲]: 1
设置格式[蒲]: 字体: （默认）Times New Roman, 非倾斜
删除[蒲]: hospital
删除[蒲]: 2
删除[蒲]: 0

nursing patients with dysphagia	No	74 (35.1)	33 (42.9)	5 (12.5)	7 (28.0)
Related training for dysphagia	Yes	109 (51.7)	28 (36.4)	29 (72.5)	9 (36.0)
	No	102 (48.3)	49 (63.6)	11 (27.5)	16 (64.0)
Specialized training (geriatric, swallowing and rehabilitation)	Yes	31 (14.7)	6 (7.8)	14 (35.0)	4 (16.0)
	No	180 (85.3)	71 (92.2)	26 (65.0)	21 (84.0)

KAB scores

KAB scores and their standardized scores by percentage were listed in Table 2. The knowledge and behavior scores were medium, but the attitude scores were high.

Table 2. Participants' scores of Knowledge, Attitudes and Behavior in dysphagia assessment

Instruments and score range	Mean±SD	Percentige rating score	Minimum	Maximum	Range
Medical staff's Knowledge of dysphagia assessment (0-24.4)	15.3±2.7	62.9	3.6	22.2	18.6
Medical staff's Attitude of dysphagia assessment(8-40)	35.9±4.9	89.7	8.0	40.0	32.0
Medical staff's Behavior of dysphagia assessment(13-65)	41.4±14.4	63.7	13.0	65.0	52.0
Medical staff's KAB of dysphagia assessment(21-129.4)	92.7±17.5	71.6	37.0	125.0	88.0

Note. KAB=Knowledge, Attitudes and Behavior

KAB Differences in 4 province

As seen in Table 3, medical staff in Shaanxi province were more willing to assess dysphagia and assessed more frequently than other provinces.

Table 3. Differences of medical staff 's Knowledge, Attitudes and Behavior of dysphagia assessment in different province(one-way analysis of variance)

Instruments and subdomains	Guangdong	Hunan	Guangxi	Shaanxi	F	p valu
	Mean±SD					
Medical staff's Knowledge of dysphagia assessment(0-24.4)	15.4±2.5	14.9±2.4	16.6±2.5	15.2±3.7	2.519	0.05
Medical staff's Attitude of dysphagia assessment(8-40)	35.7±5.1	35.2±4.7	37.1±4.1	37.7±4.0	3.023	0.03(
Medical staff's Behavior of dysphagia assessment(13-65)	40.8±15.5	38.5±12.0	43.5±13.1	49.1±11.1	5.273	0.001
Medical staff's KAB of	91.8±18.7	88.6±14.2	97.2±14.6	102.0±14.7	6.080	<0.001

- 删除[蒲]: n
- 设置格式[蒲]: 字体: （默认）Times New Roman, 非倾斜
- 删除[蒲]: 07
- 删除[蒲]: 86
- 删除[蒲]: 0
- 删除[蒲]: 119
- 删除[蒲]: 33.71
- 删除[蒲]: r
- 设置格式[蒲]: 字体: （默认）Times New Roman, 加粗
- 删除[蒲]: y
- 设置格式[蒲]: 字体: （默认）Times New Roman, 非倾斜
- 删除[蒲]: 66
- 删除[蒲]: 36
- 删除[蒲]: 0
- 删除[蒲]: 175
- 删除[蒲]: 49.58
- 删除[蒲]: n
- 设置格式[蒲]: 字体: （默认）Times New Roman, 非倾斜
- 删除[蒲]: 4
- 删除[蒲]: 4
- 删除[蒲]: 0
- 删除[蒲]: 178
- 删除[蒲]: 50.42
- 设置格式[蒲]: 字体: （默认）Times New Roman, 加粗
- 删除[蒲]: y
- 设置格式[蒲]: 字体: （默认）Times New Roman, 非倾斜
- 删除[蒲]: 69
- 删除[蒲]: 79
- 删除[蒲]: 0
- 删除[蒲]: 55
- 删除[蒲]: 15.58
- 删除[蒲]: n
- 删除[蒲]: 1
- 删除[蒲]: 1

dysphagia assessment(21-129.4)

Note. Results of post hoc multiple comparisons between provinces

*Medical staff in Shaanxi province were more willing to assess dysphagia than Guangdong(p=0.037) and Hunan(p=0.023)

**Medical staff in Shaanxi province were more frequently evaluated for dysphagia than Guangdong(p=0.001) and Hunan(p<0.001)

***The scores of the questionnaire in Shaanxi province was higher than Guangdong(p=0.002) and Hunan(p<0.001)

KAB=Knowledge, Attitudes and Behavior

Univariate analysis

KAB scores were compared among the different demographic subgroups. Detailed findings were presented in Table 4. The KAB of medical staff was not significantly different between positions, titles, and education.

Table 4. Differences of medical staff 's Knowledge, Attitudes and Behavior of dysphagia assessment in Sociodemographic, training and working experience characteristics (n=353)

Characteristic		n(%)	KAB Mean±SD	95%CI	Univariate analy (t/F, p)
Hospital level	Level 1	14 (4.0)	81.3±16.8	(71.6,91.0)	F=4.680, p=0.0
	Level 2	53 (15.0)	89.3±19.1	(84.0,94.5)	
	Level 3	286 (81.0)	93.8±17.0	(91.9,95.8)	
Hospital type	The general hospital	336 (95.2)	93.4±17.2	(91.6,95.2)	t=3.585, p<0.00
	Other hospital	17 (4.8)	78.0±18.2	(68.7,87.4)	
Department	Department (Neurology, Rehabilitation, Geriatrics)	177 (50.1)	100.6±13.6	(98.6,102.6)	t=9.566, p<0.00
	Other department	176 (49.9)	84.7±17.5	(82.1,87.3)	
Position	Clinical nurse	234 (66.3)	93.6±17.0	(91.5,95.8)	F=1.126, p=0.3
	Clinical doctor	68 (19.3)	91.1±18.7	(86.6,95.7)	
	Management personnel	34 (9.6)	92.5±16.9	(86.6,98.4)	
	Community nurses	12 (3.4)	87.3±22.7	(72.9,101.7)	
	Others	5 (1.4)	81.3±13.3	(64.8,97.7)	
Title	Primary title	165 (46.7)	92.2±17.6	(89.5,94.9)	F=0.342, p=0.7
	Medium-grade professional title	148 (41.9)	92.7±17.6	(89.8,95.5)	
	Senior title of professional	40 (11.3)	94.7±17.2	(89.2,100.2)	
Working years in the field of	None	113 (32.0)	82.5±17.0	(79.3,85.6)	F=24.700, p<0.0
	<3 years	63 (17.9)	93.2±17.6	(88.7,97.6)	

删除[蒲]: r
删除[蒲]: level
删除[蒲]: a
删除[蒲]: is
设置格式[蒲]: 字体: （默认）Times New Roman, 加粗
删除[蒲]: (
删除[蒲]:)
删除[蒲]: 1
设置格式[蒲]: 字体: （默认）Times New Roman, 非倾斜
删除[蒲]: hospital
删除[蒲]: 3.97
删除[蒲]: 1
删除[蒲]: (
删除[蒲]: 78)
删除[蒲]: 3
删除[蒲]: 0
删除[蒲]: h
删除[蒲]: 1
设置格式[蒲]: 字体: （默认）Times New Roman, 非倾斜
删除[蒲]: hospital
删除[蒲]: 1
删除[蒲]: 26
删除[蒲]: (
删除[蒲]: 1)
删除[蒲]: 3.99
删除[蒲]: 2
删除[蒲]: 1
设置格式[蒲]: 字体: （默认）Times New Roman, 非倾斜
删除[蒲]: hospital
删除[蒲]: 2
删除[蒲]: 4
删除[蒲]: (
删除[蒲]: 2)
删除[蒲]: 86

dysphagia related diseases	3-5 years	54 (15.3)	98.1±15.8	(93.8,102.4)	
	≥5 years	123 (34.8)	99.4±14.2	(96.9,101.9)	
Education	Junior college and below	62 (17.5)	89.7±19.4	(84.8,94.6)	F=1.148, p=0.3[illegible]
	Bachelor	253 (71.7)	93.4±17.0	(91.3,95.5)	
	Master degree or above	38 (10.8)	92.2±17.8	(86.4,98.1)	
Experience in nursing patients with dysphagia	Yes	234 (66.3)	97.8±15.1	(95.8,99.7)	t=8.019, p<0.0[illegible]
	No	119 (33.7)	82.6±17.7	(79.4,85.8)	
Related training for dysphagia	Yes	175 (49.6)	99.9±14.6	(97.7,102.0)	t=8.397, p<0.0[illegible]
	No	178 (50.4)	85.6±17.3	(83.0,88.1)	
Specialized training (geriatric, swallowing and rehabilitation)	Yes	55 (15.6)	106.2±12.3	(102.9,109.5)	t=8.285, p<0.0[illegible]
	No	298 (84.4)	90.2±17.2	(88.2,92.1)	

Note.Abbreviation: CI=confidence interval, KAB=Knowledge, Attitudes and Behavior.

Predictors of medical staff's KAB of dysphagia assessment

In the final regression analysis, all the categorical variables were transformed into dummy variables showed in S2 Table. Dummy multiple regression analysis was conducted to identify relevant predictors for medical staff's KAB in dysphagia assessment. As seen in Table 5, working years in the field of dysphagia related diseases, specialized training (geriatric, swallowing and rehabilitation); experience in nursing patients with dysphagia; Department (Neurology, Rehabilitation, Geriatrics) and related training for dysphagia were significant predictors of medical staff's KAB of dysphagia assessment, accounting for 35.1% of variance.

Table 5. Factors related to Knowledge, Attitudes and Behavior by stepwise regression

Variables	Std. β	t	p value
**Hospital level (ref: level 2): level 1**	-0.026	-0.579	0.563
**Hospital level (ref: level 2): level 3**	0.045	1.025	0.306
**Hospital type (ref: other hospital): the general hospital**	0.065	1.470	0.142
**Working years in the field of dysphagia related diseases (ref: <3 years): none**	-0.130	-2.530	0.012
**Working years in the field of dysphagia related diseases (ref: <3 years): 3-5 years**	0.020	0.449	0.654
**Working years in the field of dysphagia related diseases (ref: <3 years): ≥5 years**	0.023	0.454	0.650
**Specialized training (geriatric, swallowing and rehabilitation) (ref:**	0.148	3.171	0.002

设置格式[蒲]: 字体: （默认）Times New Roman, 非倾斜
删除[蒲]: 0
删除[蒲]: 06
删除[蒲]: (
删除[蒲]: 79)
删除[蒲]: 75
删除[蒲]: 37
设置格式[蒲]: 字体: （默认）Times New Roman, 非倾斜
删除[蒲]: 4
删除[蒲]: 39
删除[蒲]: (
删除[蒲]: 16)
删除[蒲]: 86
删除[蒲]: 1
设置格式[蒲]: 字体: （默认）Times New Roman, 非倾斜
删除[蒲]: 6
删除[蒲]: 0
删除[蒲]: (
删除[蒲]: 2)
删除[蒲]: 77
删除[蒲]: 3
删除[蒲]: e
设置格式[蒲]: 字体: （默认）Times New Roman, 加粗
设置格式[蒲]: 字体: （默认）Times New Roman, 非倾斜
删除[蒲]: 67
删除[蒲]: 4
删除[蒲]: (
删除[蒲]: 6.97)
删除[蒲]: 4
删除[蒲]: 4
设置格式[蒲]: 字体: （默认）Times New Roman, 非倾斜
删除[蒲]: 77
删除[蒲]: 4
删除[蒲]: (

No): Yes			
Experience in nursing patients with dysphagia(ref: No): Yes	0.172	3.491	0.001
Department (Neurology, Rehabilitation, Geriatrics) (ref: No): Yes	0.239	4.631	<0.001
Related training for dysphagia(ref: No): Yes	0.196	4.026	<0.001

note. Std. β, standardized β coefficient

Adjusted R^2=0.351, F=39.130, p<0.001

Discussion

Status of KAB of dysphagia assessment

The knowledge of medical staff on dysphagia assessment directly affects the quality of care and the prognosis of patients. However, most nurses lack the knowledge of dysphagia assessment, which may be related to lack of relevant training and attention paid by medical institutions to the knowledge education of nurses[18]. To build an appropriate and effective training program, it is necessary to combine the influencing factors of nurses' knowledge, attitude and behavior in the assessment of swallowing disorders. There is no research on the influencing factors of knowledge, attitude and behavior of dysphagia assessment in China. This study was conducted in multiple regions (4 provinces), multiple populations (doctors, clinical and community nurses, technicians, etc.) and multiple departments (neurosurgery and general departments) aiming to find as different data and features as possible to serving the training program.

In the study, the KAB score were 92.7±17.5, and the percentile rating score was 71.6. The percentile rank was 54.0, which means that 54.0% of participants' KAB scores were above the average score. According to the percentile rating score in Table 2, Knowledge and Behavior scores were medium, and attitude scores were high. This showed that the attitude score of medical staff is higher than the behavior score because the standardized score of attitude is 89.7, which is much higher than the standardized score of behavior 63.7.

The results of this study are different from study of Sun Qian et al[19]. The percentige rating score of knowledge and attitude in this study is similar to study of Sun Qian, which is at the medium and high level respectively. However, the percentige rating score of behavior in this study is slightly lower than study of Sun Qian (63.7 VS 71.0). The reasons may be as follows: Firstly, different participants and locations were surveyed in two studies. Sun Qian surveyed nurses in 17 Grade III hospitals in Beijing, while this study surveyed medical staff in hospitals of different levels in four provinces. Secondly, different departments were surveyed in two studies. Sun Qian only investigated the department of neurosurgery, which is known to pay more attention to the evaluation practice of swallowing disorders than other departments.

设置格式[蒲]: 字体: （默认）Times New Roman, 加粗
设置格式[蒲]: 字体: （默认）Times New Roman, 非倾斜
删除[蒲]: (yes)
设置格式[蒲]: 字体: （默认）Times New Roman, 加粗
设置格式[蒲]: 字体: （默认）Times New Roman, 非倾斜
删除[蒲]: (yes)
删除[蒲]: r
设置格式[蒲]: 字体: （默认）Times New Roman, 加粗
设置格式[蒲]: 字体: （默认）Times New Roman, 非倾斜
设置格式[蒲]: 字体: 三号, 非倾斜
设置格式[蒲]: 字体: 非倾斜
删除[蒲]: the
删除[蒲]: the
删除[蒲]: the lack of
设置格式[蒲]: 字体: 非倾斜, 上标
删除[蒲]: a
设置格式[蒲]: 字体: 非倾斜
设置格式[蒲]: 缩进: 首行缩进: 1 字符
删除[蒲]: our
删除[蒲]: s
删除[蒲]: 65
删除[蒲]: 52
删除[蒲]: 0
删除[蒲]: 3.99
删除[蒲]: 3.99
删除[蒲]: . As can be seen from Table 2,
删除[蒲]: 3
删除[蒲]: 2
设置格式[蒲]: 字体: 非倾斜, 上标
删除[蒲]: a17
设置格式[蒲]: 字体: 非倾斜
设置格式[蒲]: 字体: 非倾斜, 上标
设置格式[蒲]: 字体: 非倾斜
删除[蒲]: level

The lack of knowledge in this study was reflected in the following aspects: First, 80.4% and 76.3% participants did not know how to perform the volume-viscosity swallowing test (VVST) and water swallow test, respectively. This is related to the fact that 84.4% of the participants did not have Specialized training (geriatric, swallowing and rehabilitation) . Second, nearly half of the participants did not know the concept of mouthful size, food requirements for patients with dysphagia, or how to feed patients with hemiplegia. The deficiencies also included using the wrong utensils, incorrect positioning of the patient during feeding, or giving patients fluids or food of the wrong consistency. There's a study demonstrated that Speech-language pathologists varied in their views on the extent of their role in managing mealtime difficulties. Additionally, their self-rated knowledge of mealtime difficulties was lower than their dysphagia knowledge[20]. These indicated that the clinical attention to managing mealtime difficulties with dysphagia is insufficient. A study demonstrated that knowledge deficit was an important barrier in dysphagia care for nurses, and this can be improved with a short training[21]. Further research to develop efficient and effective training for care staff supporting mealtime difficulties and dysphagia is essential.

The lack of behavior in the study was mainly reflected in the following aspects. First, in 79.1% of the participants' departments, when stroke patients were screened for dysphagia, no further instrumental examinations were performed. Second, in 13.9% of the participants' departments, swallowing function was not screened in stroke patients after admission. Nurse stress with patient characteristics and workload may affect whether a swallow screen is undertaken[22]. The time of screening or assessment of OD prevalence was recorded. OD prevalence estimates from the hospital setting were reported as the time post-stroke or time from admission. Time post-stroke ranged from the hyperacute phase[23], ≤24h post-stroke, to the acute phase[24]; 1-7 days, to the early subacute phase[25];7 days-3 months, to the recovery phase[26]. Therefore, we should screen for or assess dysphagia at the right time based on the patient's condition and identify reliable and standardized assessment methods for screening post-stroke dysphagia(PSD) in acute stroke patients to ensure reliable estimates [27].

One study reported that 35.8% of professionals did not know the definition of dysphagia as a swallowing disorder[28]. Lack of knowledge among healthcare professionals can lead to inappropriate practices and increase the complications of dysphagia, such as aspiration pneumonia and malnutrition. This is an important barrier in the management of patients with dysphagia. One study investigated the Knowledge, Attitude and Practice of healthcare providers in Iran, and the results showed that very few participants were familiar with a standard test for screening and assessment of dysphagia (11.9%). A total of 74.7% were willing to participate in a workshop on dysphagia; the main pitfalls in their country lie in practice [29]. Another study reported that there seems to be limited awareness among ICU practitioners that patients are at risk of dysphagia, particularly as ventilation persists, protocols, routine assessment, and instrumental assessments are generally not used[30]. Therefore, it is important to improve the practice of dysphagia assessment.

删除[蒲]: our
删除[蒲]: is
删除[蒲]: 1
删除[蒲]: 29
删除[蒲]: 2
删除[蒲]: also
设置格式[蒲]: 字体: 非倾斜, 上标
删除[蒲]: 17
设置格式[蒲]: 字体: 非倾斜
设置格式[蒲]: 字体: 非倾斜, 上标
设置格式[蒲]: 字体: 非倾斜
设置格式[蒲]: 字体: 非倾斜, 上标
删除[蒲]: 18
设置格式[蒲]: 字体: 非倾斜
设置格式[蒲]: 字体: 非倾斜, 上标
设置格式[蒲]: 字体: 非倾斜
删除[蒲]: The deficiencies included using the wrong uten
删除[蒲]: our
删除[蒲]: is
删除[蒲]: 2
删除[蒲]: 2
删除[蒲]:
删除[蒲]: 19
删除[蒲]: 0
删除[蒲]: :
删除[蒲]: 1
删除[蒲]: 2
删除[蒲]: 3
删除[蒲]: 4
删除[蒲]: 5
删除[蒲]: 6
删除[蒲]: 27

Comparison of medical staff's KAB in different Provinces

In the study, the KAB of participants in Shaanxi Province was higher than that of participants in other provinces. The reasons for this are as follows. First, 95.0% of the participants in Shaanxi Province were from neurological, rehabilitation, and elderly related departments that mainly treat patients with stroke. There are many situations in the workplace that require the assessment of swallowing disorders. Second, 87.5% of participants in Shaanxi Province had experience in nursing patients with dysphagia.

Developing training resources by predictors of KAB of dysphagia assessment

There is still plenty of room to improve medical staff's Knowledge, Attitudes and Behavior regarding dysphagia assessment. Multiple linear regression results indicated that experience in nursing patients with dysphagia, related training for dysphagia, working years in the field of dysphagia related diseases, specialized training (geriatric, swallowing and rehabilitation) and department (Neurology, Rehabilitation, Geriatrics) were significant predictors. Consequently, the following countermeasures were proposed. The following countermeasures were based on the theory of Knowledge-Attitude-Practice and Self-efficacy.

Strengthening relevant training to improve Knowledge of dysphagia assessment

Knowledge-attitude-practice (KAP) theory is widely used in behavioral psychology[31]. This theory emphasizes that change in an individual's behavior consists of three continuous processes: acquiring knowledge, establishing beliefs and producing behavior, and paying attention to their causality and progressive relationship. The diagnosis and management of dysphagia requires comprehensive knowledge of diverse etiologies, with a systematic approach for the assessment of symptoms, selection of investigations, and appropriate treatment to relieve symptoms[32]. Meanwhile, staff who provide mealtime assistance to people with dysphagia require adequate training to ensure that mealtimes are safe and enjoyable[33]. In this study, knowledge consisted of an assessment approach, dysphagia symptoms, mealtime assistance and so on. The mean score of medical staff was 15.3±2.7, which was at the middle level, and 49.6% participants had received related training for dysphagia(35.8% in other departments VS 63.3% in Neurology, Rehabilitation or Geriatrics department). Only 15.6% of the participants had received Specialized training (geriatric, swallowing and rehabilitation) (8.5% in other departments VS 22.6% in Neurology, Rehabilitation or Geriatrics department). Medical staff in Neurology, Rehabilitation or Geriatrics department seldom receive related training for dysphagia, which leads to less knowledge of assessment or screening for dysphagia. Therefore, it is more difficult for medical staff to establish positive beliefs regarding dysphagia assessment and screening. To standardize the management of dysphagia,

设置格式[蒲]: 字体: 三号, 非倾斜
设置格式[蒲]: 字体: 非倾斜
删除[蒲]: our
删除[蒲]: came
删除[蒲]: :
删除[蒲]: :
删除[蒲]: 28
删除[蒲]: 29
删除[蒲]: 0
删除[蒲]: ,
删除[蒲]: 4
删除[蒲]: 66
删除[蒲]: 58
删除[蒲]: 0
删除[蒲]: 28
删除[蒲]: neurological, rehabilitation, and elderly related departments
删除[蒲]: 58
删除[蒲]: specialized training in geriatric, swallowing, and rehabilitation
删除[蒲]: 2
删除[蒲]: 0
删除[蒲]: neurological, rehabilitation, and elderly related departments

hospitals should take the rate of dysphagia assessment as a quality control index and promote training in dysphagia assessment. Examples include the Volume-Viscosity Swallow Test(V-VST), water swallow test, eating assessment Tool-10(EAT-10), Mann Assessment of Swallowing Ability (MASA) and so on.

Establishing positive belief in assessment of dysphagia

According to KAP theory, beliefs and attitudes are the motive forces for individuals to produce related behaviors[31]. As the results of study, the attitude score of medical staff was 35.9±4.9, which was at the high level. This finding suggested that many people are willing to screen and evaluate patients with dysphagia. This was a good trend. Nurses gained a sense of value by assessing and caring for patients with dysphagia. Dysphagia screening is often the focus of hospitalized stroke patients; however, dysphagia can also occur in other hospitalized patients and outpatients. Dysphagia can be overlooked by nurses and clinicians, and it is important to educate nurses on the importance of dysphagia screening [34].

Accumulate dysphagia assessment experience through work and study

Self-efficacy refers to an individual's conviction of their capacity to perform a specific activity. Individuals gain self-efficacy over time as they acquire a range of talents, such as social, cognitive, physical, and linguistic abilities, via life experiences[35]. Efficacy expectations are dynamic and are both appraised and enhanced by four mechanisms[36-37]: (1) enactive mastery experience or successful performance of the activity of interest; (2) verbal persuasion or encouragement, given by a credible source that the individual is capable of performing the activity of interest; (3) vicarious experience or seeing like individuals perform a specific activity; and (4) physiological and affective states such as pain, fatigue, anxiety, hunger, or dizziness associated with a given activity. These mechanisms to drive efficacy expectations are the concepts upon which the intervention is built to encourage behavioral change. In this study, experience in nursing patients with dysphagia, working years in the field of dysphagia related diseases, and department (Neurology, Rehabilitation Geriatrics) were significant predictors of medical staff's Knowledge, Attitudes and Behavior of dysphagia assessment. The results are consistent with the self-efficacy theory. This suggested that if conditions permit, the medical staff in other departments with no experience of dysphagia patients' nursing should transfer to Neurology, Rehabilitation or Geriatrics department to be trained and accumulate relevant experience. Alternatively, the hospital organizes regular workshops for dysphagia assessment and nurses are required to participate in practice.

Establish systematic inter-professional collaboration in dysphagia management

A study showed that the main theme "limited professional services" describes how

删除[蒲]: and
删除[蒲]: .
删除[蒲]: 28
删除[蒲]: 89
删除[蒲]: 86
删除[蒲]: s
删除[蒲]: therefore
删除[蒲]: 1
删除[蒲]: 2
删除[蒲]: 3
删除[蒲]: 4
删除[蒲]: s
删除[蒲]: neurological, rehabilitation, and elderly related
删除[蒲]: s

patients received little support from healthcare professionals and had to rely on themselves to adapt to life with dysphagia[38]. The common goal of preventing aspiration and rehabilitating patients' ability to swallow safety is based on dysphagia assessment, using appropriate therapeutic interventions, sharing knowledge, and improving skills among professional groups that consist of nurses, physicians, occupational therapists, and speech-language pathologists(SLPs)[39].

The strengths and limitations of this study

First, participants in this study were from four provinces in China, showing the differences in knowledge, attitude and behavior of medical staff in different regions in dysphagia assessment. Second, this study explored the influencing factors of medical staff's knowledge, attitude and behavior in tdysphagia assessment, and provided guidance and suggestions for the future organization of relevant training. But, this study only investigated hospital and community nurses, and did not investigate healthcare professionals in other different Settings such as nursing home settings. Future studies should focus on the evaluation of dysphagia and provide training for the assessment of swallowing disorders to medical staff in nursing home settings, social health service centers, and palliative care facilities.

Conclusions

The attitude of Chinese medical staff to dysphagia assessment is positive, but the knowledge and behavior needs to be improved, and the attitude is separated from the knowledge and the behavior.This study findings also implied that nursing experience, training, and work for patients with swallowing disorders could have positive effects on the Knowledge, Attitudes and Behavior of medical staff regarding dysphagia assessment. The medical management department should take various forms to train the knowledge and skills related to the evaluation of swallowing disorders. Hospital administrators should provide relevant resources, such as videos of dysphagia assessment, training centers for the assessment of dysphagia, and swallowing specialist nurses. It is important that health policies fully recognize the role of training and support systems in caring for people with dysphagia.

Conflict of interest statement

The authors declare that there are no conflict of interests, we do not have any possible conflicts of interest.

Data access statement

The data was deposited at Dryad (DOI: https://doi.org/10.5061/dryad.djh9w0w70).

Supporting information

S1 Table. The scoring methods and rules of the questionnaire. (DOC)

Table 1 The scoring methods and rules of the questionnaire

Domains	Items	n	scoring methods and rules
Knowledge	True or False	17	1 point is scored for correct answer. 0 point

删除[蒲]: 5

删除[蒲]: 6

设置格式[蒲]: 字体: 三号, 加粗, 非倾斜

设置格式[蒲]: 字体: 非倾斜

删除[蒲]: the

删除[蒲]: of swallowing disorders

设置格式[蒲]: 字体: 小二, 非倾斜

删除[蒲]: **and Implications for future Research**

设置格式[蒲]: 字体: 非倾斜

删除[蒲]: T

删除[蒲]: the

删除[蒲]: y

删除[蒲]: reatment pathways, including early assessment and diagnosis of dysphagia, should be a priority for healthcare professionals working in different settings with populations at risk for dysphagia. Most studies have focused on medical staff in hospitals, and few have focused on medical staff from ...

设置格式[蒲]: 字体: 小二, 非倾斜

设置格式[蒲]: 字体: 非倾斜

设置格式[蒲]: 字体: 小二, 非倾斜

设置格式[蒲]: 字体: 非倾斜

删除[蒲]:

删除[蒲]: that support the findings of this study are available from the corresponding author, [Juanhui Chen], upon reasonable request

设置格式[蒲]: 字体: 小二, 加粗, 非倾斜

设置格式[蒲]: 字体: 非倾斜

设置格式[蒲]: 左

设置格式[蒲]: 居中

带格式表格[蒲]

	questions		is scored for incorrect answer.
	Single-choice questions	4	
	Multiple-choice questions	4	Count 0.2/ option when selecting the correct option. Count 0 when the wrong option was selected or the correct and wrong options were selected simultaneously For example, a multiple-choice question had three correct choices that score 0.6 when participant selected three correct choices.
Attitudes	Likert-five-point-scale	8	Likert 5-level scoring method. Ranging from "strongly agreed" to "strongly disagreed". 5 point for "strongly agreed", 1 point for "strongly disagreed"
Behavior	Likert-five-point-scale	13	Likert 5-level scoring method. Ranging from "the most frequently" to "the least frequently. 5point for "the most frequently", 1 point for "the least frequently

S2 Table. Dummy variable settings. (DOC)

Acknowledgements

The authors would like to thank all participated medical staff who took the time to complete survey and made this study possible.

Author contributions

Conceptualization, Statistical analysis, Writing-review & editing: Juanhui Chen
Investigation: Juanhui Chen, Wenqiu Ye, Xingyun Zheng, Wenna Wu, Yuebao Chen, Yinjuan Chen

References

[1] GBD 2019 Stroke Collaborators. Global, regional, and national burden of stroke and its risk factors, 1990-2019: a systematic analysis for the Global Burden of Disease Study 2019. Lancet Neurol. 2021;20(10):795-820. doi:10.1016/S1474-4422(21)00252-0.

[2] Vilardell N, Rofes L, Nascimento WV, Muriana D, Palomeras E, Clavé P. Cough reflex attenuation and swallowing dysfunction in sub-acute post-stroke patients: prevalence, risk factors, and clinical outcome. Neurogastroenterol Motil. 2017 Jan;29(1). doi: 10.1111/nmo.12910. Epub 2016 Jul 18. PMID: 27424849.

[3] Matsumura T, Mitani Y, Oki Y, Fujimoto Y, Ishikawa A. Risk factors for the onset of aspiration pneumonia among stroke patients in the recovery stage. Nihon Ronen Igakkai Zasshi. 2014;51(4):364-8.doi: 10.3143/geriatrics.51.364. PMID: 25327371.

[4] Finnegan BS, Meighan MM, Warren NC, Hatfield MK, Alexeeff S, Lipiz J, et al. Validation Study of Kaiser Permanente Bedside Dysphagia Screening Tool in Acute

设置格式[蒲]: 字体: 小二, 非倾斜

删除[蒲]: We

设置格式[蒲]: 字体: 非倾斜

删除[蒲]: would like to thank Shenzhen Longgang District Science and Technology Innovation Bureau for providing the Unfunded Medical and Health Technology Plan(No. LGKCYLWS2020128).

设置格式[蒲]: 字体: 小二, 非倾斜

设置格式[蒲]: 字体: 非倾斜

删除[蒲]: Johnson, C. O., Nguyen, M., Roth, G. A., et al. (2019). Global, regional, and national burden of stroke, 1990–2016: a systematic analysis for the Global Burden of Disease Study 2016. Lancet Neurology, 18(5), 439–458. https://doi.org/10.1016/ S1474-4422(19)30034-1

删除[蒲]: .

删除[蒲]: .

删除[蒲]: [J]

删除[蒲]: ,

删除[蒲]: ,

删除[蒲]: ,

删除[蒲]: ,

删除[蒲]:

删除[蒲]: ,

删除[蒲]: ,

删除[蒲]: ,

删除[蒲]: ,

Stroke Patients. Perm J. 2020 Dec;24:1. doi: 10.7812/TPP/19.230. PMID: 33482958; PMCID: PMC7849282.
[5] Baijens LW, Clavé P, Cras P, Ekberg O, Forster A, Kolb GF, et al. European society for swallowing disorders-European union geriatric medicine society white paper: oropharyngeal dysphagia as a geriatric syndrome. Clin Interv Aging. 2016 Oct 7;11:1403-1428. doi: 10.2147/CIA.S107750. PMID: 27785002; PMCID: PMC5063605.
[6] Rivelsrud MC, Hartelius L, Bergström L, Løvstad M, Speyer R. Prevalence of Oropharyngeal Dysphagia in Adults in Different Healthcare Settings: A Systematic Review and Meta-analyses. Dysphagia. 2023 Feb;38(1):76-121. doi: 10.1007/s00455-022-10465-x. Epub 2022 May 31. PMID: 35639156; PMCID: PMC9873728.
[7] Blanař V, Hödl M, Lohrmann C, Amir Y, Eglseer D. Dysphagia and factors associated with malnutrition risk: a 5-year multicentre study. J Adv Nurs.2019;75(12):3566–76.doi: 10.1111/jan.14188. Epub 2019 Sep 10. PMID: 31452231.
[8] Huppertz VAL, Halfens RJG, van Helvoort A, de Groot LCPGM, Baijens LWJ, Schols JMGA. Association between Oropharyngeal Dysphagia and Malnutrition in Dutch Nursing Home Residents: Results of the National Prevalence Measurement of Quality of Care. J Nutr Health Aging. 2018;22(10):1246-1252. doi: 10.1007/s12603-018-1103-8. PMID: 30498833; PMCID: PMC6302767.
[9] Beharry A, Michel P, Faouzi M, Kuntzer T, Schweizer V, Diserens K. Predictive Factors of Swallowing Disorders and Bronchopneumonia in Acute Ischemic Stroke. J Stroke Cerebrovasc Dis. 2019 Aug;28(8):2148-2154. doi: 10.1016/j.jstrokecerebrovasdis.2019.04.025. Epub 2019 May 22. PMID: 31129105.
[10] Speyer R, Cordier R, Farneti D, Nascimento W, Pilz W, Verin E, et al. White Paper by the European Society for Swallowing Disorders: Screening and Non-instrumental Assessment for Dysphagia in Adults. Dysphagia. 2022 Apr;37(2):333-349. doi: 10.1007/s00455-021-10283-7. Epub 2021 Mar 31. PMID: 33787994; PMCID: PMC8009935.
[11] Wilkinson JM, Codipilly DC, Wilfahrt RP. Dysphagia: Evaluation and Collaborative Management. Am Fam Physician. 2021 Jan 15;103(2):97-106. PMID: 33448766.
[12] Dumican M, Thijs Z, Harper K. Clinical practice patterns of speech-language pathologists for screening and identifying dysphagia. Int J Lang Commun Disord. 2023 Nov-Dec;58(6):2062-2076. doi: 10.1111/1460-6984.12921. Epub 2023 Jun 27. PMID: 37376825.
[13] Tan L, Gan G, Hum A, Lee A. A Stepwise, Mixed-Method Study Approach to Identify the Barriers to Dysphagia Care in Hospice Care Nurses. J Hosp Palliat Nurs. 2018 Feb;20(1):88-94. doi: 10.1097/NJH.0000000000000404. PMID: 30063619.
[14] Zhang Y, Liu Y, Li W, Lin Y. Investigation on the knowledge and intervention status of swallowing function training in elderly patients by nurses in Guangzhou and Shenzhen. Journal of Nurses Training, 2018,33(16):1499-1500,1531.DOI:10.16821/j.cnki.hsjx.2018.16.021.

删除[蒲]: .

删除[蒲]: -

删除[蒲]: [J] .

删除[蒲]: 7.

删除[蒲]: Smith Pamela A. Nutrition, hydration, and dysphagia in long-term care: Differing opinions on the effects of aspiration.[J] .J Am Med Dir Assoc, 2006, 7: 545-9.doi: 10.1016/j.jamda.2006.03.008. Epub 2006 May 30. PMID: 17095418

删除[蒲]: Tan Laurence, Gan Grace, Hum Allyn et al. A Stepwise, Mixed-Method Study Approach to Identify the Barriers to Dysphagia Care in Hospice Care Nurses.[J] .J Hosp Palliat Nurs, 2018, 20: 88-94.doi: 10.1097/NJH.0000000000000404. PMID: 30063619

删除[蒲]: 3a

[15] Kendall MG. kendall's advanced theory of statistics. Halsted Press; Edward Arnold, 2004.
[16] Dong X. Evidence-based management of stroke patients with dysphagia and its effect evaluation. Zhengzhou University,2018.
[17] Ma K, GUO Y, DONG X, Yang C, Zhang P, Wang M, et al. Investigation on the status of screening and evaluation of dysphagia in stroke patients by nurses in Henan Province. Chinese journal of nursing,2020,55(08):1196-1200. DOI:10.3761/j.issn.0254-1769.2020.08.016.
[18] Lv S, Zhou G, Mao Y, Zhang Y, Wang J. Research progress on the attitude, knowledge and behavior of nurses and caregivers for swallowing disorders after stroke in China. Chinese General practice nursing,2023,21(1):37-41. DOI:10.12104/j.issn.1674-4748.2023.01.009.
[19] Sun Q, Su Y, Wang Y.Investigation on the current situation of knowledge and practice in nursing of dysphagia after stroke in neurology nurses. Chinese journal of modern nursing,2020,26(23):3144-3150. DOI:10.3760/cma.j.cn115682-20200322-02063.
[20] Egan A, Andrews C, Lowit A. Dysphagia and mealtime difficulties in dementia: Speech and language therapists' practices and perspectives. Int J Lang Commun Disord. 2020 Sep;55(5):777-792. doi: 10.1111/1460-6984.12563. Epub 2020 Jul 24. PMID: 32706516.
[21] Tan L, Gan G, Hum A, Lee A. A Stepwise, Mixed-Method Study Approach to Identify the Barriers to Dysphagia Care in Hospice Care Nurses. J Hosp Palliat Nurs. 2018 Feb;20(1):88-94. doi: 10.1097/NJH.0000000000000404. PMID: 30063619.
[22] Moghadam KN, Chehrzad MM, Masouleh SR, Mardani A, Maleki M, Akhlaghi E, et al. Nursing workload in intensive care units and the influence of patient and nurse characteristics. Nurs Crit Care. 2021 Nov;26(6):425-431. doi: 10.1111/nicc.12548. Epub 2020 Sep 20. PMID: 32954619.
[23] Bernhardt J, Hayward KS, Kwakkel G, Ward NS, Wolf SL, Borschmann K, et al. Agreed definitions and a shared vision for new standards in stroke recovery research: The Stroke Recovery and Rehabilitation Roundtable taskforce. Int J Stroke. 2017 Jul;12(5):444-450. doi: 10.1177/1747493017711816. PMID: 28697708.
[24] Rajashekar D, Mouchès P, Fiehler J, Menon BK, Goyal M, Demchuk AM, et al. Structural integrity of white matter tracts as a predictor of acute ischemic stroke outcome. Int J Stroke. 2020 Dec;15(9):965-972. doi: 10.1177/1747493020915251. Epub 2020 Mar 31. PMID: 32233745.
[25] Abubakar SA, Jamoh BY. Dysphagia following acute stroke and its effect on short-term outcome. Niger Postgrad Med J.2017;24(3):182–6.doi: 10.4103/npmj.npmj_96_17. PMID: 29082909.
[26] Paik NJ, Kim WS. Recovery of Swallowing. 2021 Jan 15. In: Platz T, editor. Clinical Pathways in Stroke Rehabilitation: Evidence-based Clinical Practice Recommendations [Internet]. Cham (CH): Springer; 2021. PMID: 36315693..
[27] Banda KJ, Chu H, Kang XL, Liu D, Pien LC, Jen HJ, et al. Prevalence of dysphagia and risk of pneumonia and mortality in acute stroke patients: a meta-analysis. BMC Geriatr. 2022 May 13;22(1):420. doi:

删除[蒲]: 4

删除[蒲]: 5

删除[蒲]: .

删除[蒲]: 6

删除[蒲]: Ma Ke, GUO Yuanli, DONG Xiaofang, et al. Investigation on the status of screening and evaluation of dysphagia in stroke patients by nurses in Henan Province[J]. Chinese journal of nursing,2020,55(08):1196-1200. DOI:10.3761/j.issn.0254-1769.2020.08.016

删除[蒲]: a

删除[蒲]: a17

删除[蒲]: 17

删除[蒲]: 178

删除[蒲]: Tan L, Gan G, Hum A, et al. Honey I fed my patients: clinical improvement project on swallowing safety. Presented as a poster at the Singapore Intermediate Long Term Care Quality Conference; 2013

删除[蒲]: [18] Colodny N. Construction and validation of the Mealtime and Dysphagia Questionnaire: an instrument designed to assess nursing staff reasons for noncompliance with SLP dysphagia and feeding recommendations. Dysphagia. 2001;16(4):263-271.doi: 10.1007/s00455-001-0085-5. PMID: 11720402.

删除[蒲]: 19

删除[蒲]: 0

删除[蒲]: 1

删除[蒲]: 2

删除[蒲]: 3

删除[蒲]: 4

10.1186/s12877-022-02960-5. PMID: 35562660; PMCID: PMC9103417..
[28] Sánchez-Sánchez E, Avellaneda-López Y, García-Marín E, Ramírez-Vargas G, Díaz-Jimenez J, Ordonez FJ. Knowledge and Practice of Health Professionals in the Management of Dysphagia. Int J Environ Res Public Health. 2021 Feb 22;18(4):2139. doi: 10.3390/ijerph18042139. PMID: 33671732; PMCID: PMC7926391.. 删除[蒲]: 5
[29] Farpour S, Farpour HR, Smithard D, Kardeh B, Ghazaei F, Zafarghasempour M. Dysphagia Management in Iran: Knowledge, Attitude and Practice of Healthcare Providers. Dysphagia. 2019 Feb;34(1):105-111. doi: 10.1007/s00455-018-9919-2. Epub 2018 Jun 21. PMID: 29931606. 删除[蒲]: 6
[30] Spronk PE, Spronk LEJ, Egerod I, McGaughey J, McRae J, Rose L, et al. Dysphagia in Intensive Care Evaluation (DICE): An International Cross-Sectional Survey. Dysphagia. 2022 Dec;37(6):1451-1460. doi: 10.1007/s00455-021-10389-y. Epub 2022 Jan 29. PMID: 35092486. 删除[蒲]: 27
[31] Liu C, Liu C, Wang D, Zhang X. Knowledge, Attitudes and Intentions to Prescribe Antibiotics: A Structural Equation Modeling Study of Primary Care Institutions in Hubei, China. Int J Environ Res Public Health. 2019 Jul 5;16(13):2385. doi: 10.3390/ijerph16132385. PMID: 31284381; PMCID: PMC6651188. 删除[蒲]: 28
[32] Nigam GB, Vasant DH, Dhar A. Curriculum review : investigation and management of dysphagia. Frontline Gastroenterol. 2021 Aug 3;13(3):254-261. doi: 10.1136/flgastro-2021-101917. PMID: 35493628; PMCID: PMC8996094. 删除[蒲]: 29
[33] Reddacliff C, Hemsley B, Smith R, Dalton S, Jones S, Fitzpatrick A, et al. Examining the Content and Outcomes of Training in Dysphagia and Mealtime Management: A Systematic Review Informing Co-Design of New Training. Am J Speech Lang Pathol. 2022 May 10;31(3):1535-1552. doi: 10.1044/2022_AJSLP-21-00231. Epub 2022 Apr 4. PMID: 35377733. 删除[蒲]: 0
[34] West J, Stutzman SE, Atem F, Olson DM. Formatting an experiential learning education module to encourage dysphagia assessment in apheresis patients. J Clin Apher. 2018 Feb;33(1):72-77. doi: 10.1002/jca.21572. Epub 2017 Aug 2. PMID: 28766740. 删除[蒲]: 1
[35] Huo C, Hameed J, Nawaz A, Adnan Raheel Shah S, Albahser G, Alqahtani W, Maqsoom A, Kashif Anwar M. Scientific risk performance analysis and development of disaster management framework: A case study of developing Asian countries. J King Saud Univ Sci. 2021 Mar;33(2):101348. doi: 10.1016/j.jksus.2021.101348. Epub 2021 Jan 28. PMID: 35495615; PMCID: PMC9042590. 删除[蒲]: 2
[36] Bandura A. Self-Efficacy: The Exercise of Control. New York: WH Freeman; 1997. 删除[蒲]: 3
[37] Resnick B. Geriatric rehabilitation: the influence of efficacy beliefs and motivation. Rehabil Nurs. 2002;27(4):152–159.doi: 10.1002/j.2048-7940.2002.tb02224.x. PMID: 12116528. 删除[蒲]: 4
[38] Li C, Qiu C, Shi Y, Yang T, Shao X, Zheng D. Experiences and perceptions of stroke patients living with dysphagia: A qualitative meta-synthesis. J Clin Nurs. 2022 Apr;31(7-8):820-831. doi: 10.1111/jocn.15995. Epub 2021 Aug 8. PMID: 34369025. 删除[蒲]: 5
[39] Nielsen AH, Kaldan G, Nielsen BH, Kristensen GJ, Shiv L, Egerod I. Intensive care professionals' perspectives on dysphagia management: A focus group study. Aust 删除[蒲]: 6

Crit Care. 2023 Jul;36(4):528-535. doi: 10.1016/j.aucc.2022.04.004. Epub 2022 May 21. PMID: 35610091.

Response to reviewers

Dear editors and reviewers:

Thank you for your letter and for the reviewer's comments concerning our manuscript entitled "Predictors of medical staff's Knowledge, Attitudes and Behavior of dysphagia assessment: A Cross-Sectional study"(ID:PONE-D-23-41544). Those comments are all valuable and very helpful for revising and improving our paper, as well as the important guiding significance to our researches. We have studied comments carefully and have made correction which we hope meet with approval.

We submited four items as follow:

1. Response to Reviewers
2. Revised manuscript with track changes
3. Manuscript (revised)
4. updated cover letter

Below, please find a response to each of the reviewer's comments. We have revised manuscript with track changes. We hope that the revisions in the manuscript and our accompanying responses will meet editors' and reviewers' criteria.
We shall look forward to hearing from you at your earliest convenience.
Yours sincerely
Juanhui Chen

Acdemic editor

1. We note that the grant information you provided in the 'Funding Information' and 'Financial Disclosure' sections do not match.
When you resubmit, please ensure that you provide the correct grant numbers for the awards you received for your study in the 'Funding Information' section.

The author's answer: When we resubmit, we have provided the correct grant number(No. LGKCYLWS2020128) in 'Funding information' section.
Funding: Medical and Health Technology Plan of Longgang District, Shenzhen. Funding was rewarded to Juanhui Chen. The Grant award number was LGKCYLWS2020128. URL: http://www.lg.gov.cn/bmzz/kjj/xxgk/qt/tzgg/content/post_7970194.html The funders play no role in the study design, data collection and analysis, decision to publish, or preparation of the manuscript.

2. Thank you for stating the following in the Acknowledgments Section of your manuscript:
"We would like to thank Shenzhen Longgang District Science and Technology
Innovation Bureau for providing the Unfunded Medical and Health Technology
Plan(No. LGKCYLWS2020128)"

We note that you have provided funding information that is not currently declared in your Funding Statement. However, funding information should not appear in the Acknowledgments section or other areas of your manuscript. We will only publish funding information present in the Funding Statement section of the online submission form.
Please remove any funding-related text from the manuscript and let us know how you would like to update your Funding Statement. Currently, your Funding Statement reads as follows:
"The author(s) received no specific funding for this work"

Please include your amended statements within your cover letter; we will change the online submission form on your behalf.

The author's answer: We have removed any funding-related text from the manuscript, and amended financial disclosure statement whthin our cover letter as follows:
Financial disclosure statement:
Medical and Health Technology Plan of Longgang District, Shenzhen.
Funding was rewarded to Juanhui Chen. The Grant award number was LGKCYLWS2020128. URL: http://www.lg.gov.cn/bmzz/kjj/xxgk/qt/tzgg/content/post_7970194.html The funders play no role in the study design, data collection and analysis, decision to publish, or preparation of the manuscript.

3. When completing the data availability statement of the submission form, you indicated that you will make your data available on acceptance. We strongly recommend all authors decide on a data sharing plan before acceptance, as the process can be lengthy and hold up publication timelines. Please note that, though access restrictions are acceptable now, your entire data will need to be made freely accessible if your manuscript is accepted for publication. This policy applies to all data except where public deposition would breach compliance with the protocol approved by your research ethics board. If you are unable to adhere to our open data policy, please kindly revise your statement to explain your reasoning and we will seek the editor's input on an exemption. Please be assured that, once you have provided your new statement, the assessment of your exemption will not hold up the peer review process.

The author's answer: We have built a dataset to share data on Dryad. The DOI URL is https://doi.org/10.5061/dryad.djh9w0w70.
The temporary URL:
https://datadryad.org/stash/share/GQq3KacnFwuVnq8aE-fIQmF62FN5xn08L7rAYaFIyoQ

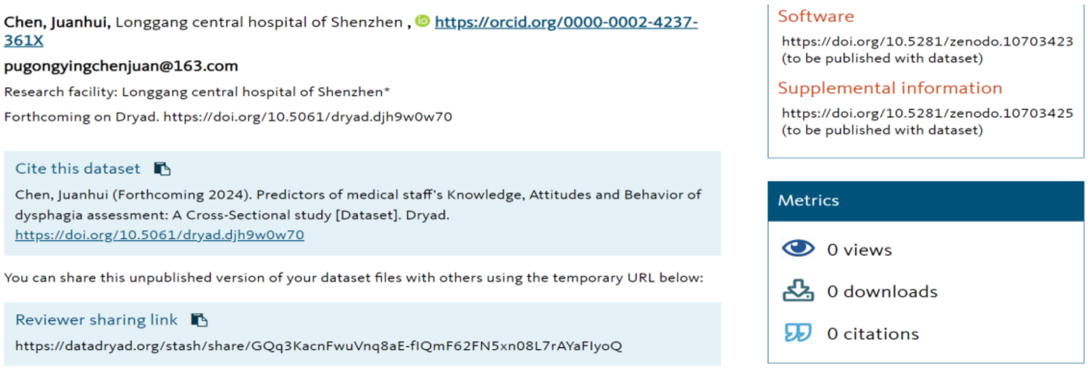
Chen, Juanhui, Longgang central hospital of Shenzhen , https://orcid.org/0000-0002-4237-361X
pugongyingchenjuan@163.com
Research facility: Longgang central hospital of Shenzhen*
Forthcoming on Dryad. https://doi.org/10.5061/dryad.djh9w0w70

Cite this dataset
Chen, Juanhui (Forthcoming 2024). Predictors of medical staff's Knowledge, Attitudes and Behavior of dysphagia assessment: A Cross-Sectional study [Dataset]. Dryad. https://doi.org/10.5061/dryad.djh9w0w70

You can share this unpublished version of your dataset files with others using the temporary URL below:

Reviewer sharing link
https://datadryad.org/stash/share/GQq3KacnFwuVnq8aE-fIQmF62FN5xn08L7rAYaFIyoQ

Software
https://doi.org/10.5281/zenodo.10703423
(to be published with dataset)
Supplemental information
https://doi.org/10.5281/zenodo.10703425
(to be published with dataset)

Metrics
0 views
0 downloads
0 citations

Data Availability Statement: Data is deposited at Dryad
(DOI: https://doi.org/10.5061/dryad.djh9w0w70).

Reviewer #1:

Abstract

1. The objective is unclear (too large). In addition, in the Purpose and Introduction, the authors mentioned "stroke". However, I do not know whether or not "stroke" was a factor associated with the inclusion criteria of participants.

The author's answer: "Stoke" was not a factor associated with the inclusion criteria of participants. We have revised the objective. The revised objective as follows [Page 1]:
This study aimed to explore the predictors of medical staff' s Knowledge, Attitudes and Behavior of dysphagia assessment, to provide reference suggestions for constructing the training program and improving the rate of dysphagia assessment.

2. The authors should not mention the number of medical staff approached (430 nurses and doctors) in the Methods. The final number of participants should be mentioned (n=353).

The author's answer: We have revised the number of medical staff in method as follows[Page 1]:
Methods: This study was a cross-sectional study. A total of 353 nurses and doctors from four provinces(Guangdong, Hunan, Guangxi, and Shaanxi) who were selected by convenience sampling......

Introduction

3. The authors wrote: "Dysphagia occurs in 37 – 78% of patients after stroke [2-4]."
However, these three references are seemingly not relevant to the sentence above.

The author's answer: We have re-searched the relevant literature and revised sentence in the introduction is "*Dysphagia occurs in 30 – 78% of patients after stroke [2-4]*". Changed References are as follows[Page 2, 14]:
[2] Vilardell N, Rofes L, Nascimento WV, Muriana D, Palomeras E, Clavé P. Cough reflex attenuation and swallowing dysfunction in sub-acute post-stroke patients: prevalence, risk factors, and clinical outcome. Neurogastroenterol Motil. 2017 Jan;29(1). doi: 10.1111/nmo.12910. Epub 2016 Jul 18. PMID: 27424849.
It is shown in reference 2 that "We included 225 PSP. Prevalence of impaired CRT was 5.8%, that of OD was 40.4% (20.4% with impaired safety of swallow), and of both impairments was, 1.8%".
[3] Matsumura T, Mitani Y, Oki Y, Fujimoto Y, Ishikawa A. Risk factors for the onset of aspiration pneumonia among stroke patients in the recovery stage. Nihon Ronen Igakkai Zasshi. 2014;51(4):364-8.doi: 10.3143/geriatrics.51.364. PMID: 25327371.
It is shown in reference 3 that "The study subjects included 76 stroke patients (mean age,

74.7±8.4 years) with dysphagia chosen from 175 stroke patients who were newly admitted to four rehabilitation hospitals". According to the above data, the incidence of dysphagia in stroke patients was 43.43%.

[4] Finnegan BS, Meighan M M, Warren NC, Hatfield MK, Alexeeff S, Lipiz J, et al. Validation Study of Kaiser Permanente Bedside Dysphagia Screening Tool in Acute Stroke Patients. Perm J. 2020 Dec;24:1. doi: 10.7812/TPP/19.230. PMID: 33482958; PMCID: PMC7849282.

It is shown in reference 4 that "Difficulty swallowing or dysphagia is common among acute stroke patients, occurring in approximately 30% to 78% of the population".

4. The Introduction is quite long. I think the authors should divide it into two paragraphs (one for the prevalence of dysphagia and one for studies involving their topic + the objectives).

The author's answer: The introduction has been divided into three paragraphs. Please see detail in the article[Page 2-3].

Methods

5. What does "Chinese RNs" mean?

The author's answer: Chinese RNs" mean Chinese nurse practitioners. We have revised in the article[Page 3].

6. "Based on the suggestion of Hair, Black, Babin, and Anderson[14], a ratio of 10 to 20 participants for each parameter was required for structural equation modeling."
Please check the reference 14.

The author's answer: We have re-searched the literature on sample size estimation, according to the sample content estimation method proposed by Kendall, the sample content of questionnaire survey should be 5-10 times the number of items.

The revised references is as follows[Page 15]:

[15] Kendall MG. kendall's advanced theory of statistics. Halsted Press; Edward Arnold, 2004.

7. "Thus, 220 – 440 participants were followed by a calculation of a 15% attrition rate, which resulted in a total sample of 430 participants in this study."
This sentence is unclear.

The author's answer: This sentence has been revised as follows[Page 3]:

According to the sample content estimation method proposed by Kendall, the sample content of questionnaire survey should be 5-10 times the number of items. There were 46 items in the questionnaire. Thus, 230-460 participants were calculated according to the ratio. Adding a 15.0% attrition rate, the total participants sample should between 265 and 529. A total of 353 nurses and doctors completed the survey in this study.

8. "An anonymous survey was conducted with permission from the research ethics committee of

the participating hospital.”
This sentence is unclear (the participating hospital).
In addition, please do not repeat the information involving the ethics in the Methods. The authors had an “Ethical statements” part already.
(“Data collection and Analysis
This study was approved by the ethics committee of the study hospital.”)

The author’s answer: We removed the information involving the ethics in the Data collection and Analysis. The sentence in the question above has been revised as follows[Page 3]:
An anonymous survey was conducted by Longgang central hospital of Shenzhen on May 23-31, 2022.

9. “The inclusion criteria were as follows: (a) Chinese RNs, doctors, or therapists with a medical background, (b) working at the hospital for more than 3 months, and (c) not internship.”
I do not know how the authors identified and approached 430 nurses and doctors. The authors used online platforms to collect data. However, the participants only came from four provinces (Guangdong Province, Hunan Province, Guangxi Province, and Shaanxi Province).

The author’s answer: Firstly, there are instructions at the beginning of the questionnaire that including The inclusion criteria. Secondly, We analyzed the data of departments, titles and positions and so on filled in by the research participants for judging whether they meet the the inclusion criteria.

10. “Before the study, I obtained the consent of the author of the original questionnaire to modify the questionnaire. This questionnaire was revised based on the existing questionnaires designed by Dr. Dong Xiaofang[15] and Master Ma Keke[16] from The First Affiliated Hospital of Zhengzhou University.”
First, “I” should be replaced by “we”.
Second, the authors mentioned two references for their questionnaire. However, the reference 15 cannot be identified. Is it a thesis?
The reference 16 cannot be found in Google/PubMed.
Can the authors attach the questionnaire as a supplementary file?

The author’s answer: “I” has been replaced by “we” in the article[Page 4]. The questionnaire has been submitted as a supplementary file. References 15 and 16 were came from Chinese researchers, and format of this Chinese references is as follows:
[16] 董小方. 基于循证构建脑卒中吞咽障碍患者管理方案及效果评价[D]. 河南:郑州大学,2018.

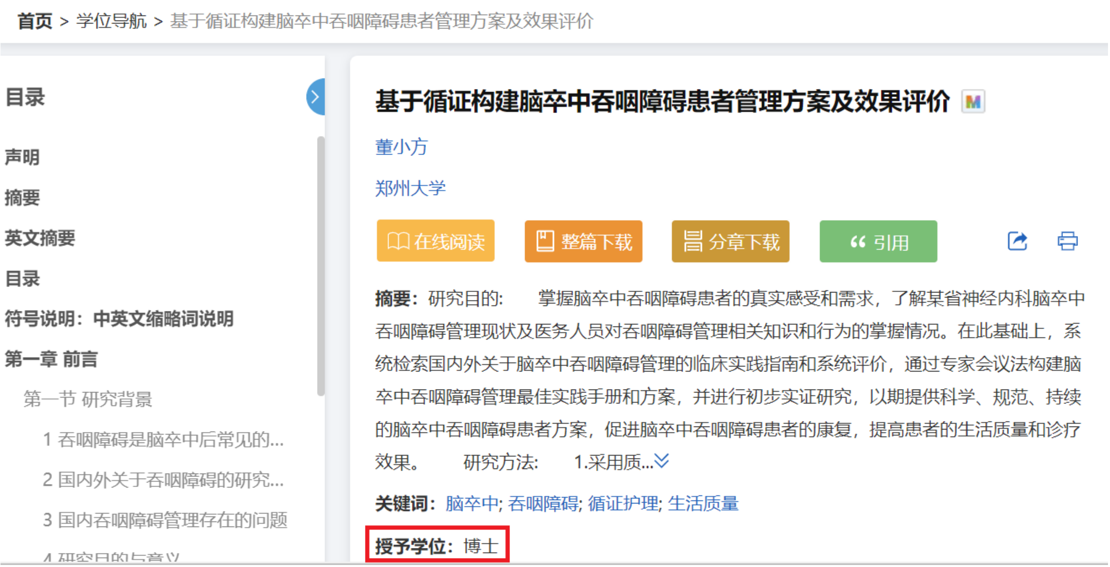

首页 > 学位导航 > 基于循证构建脑卒中吞咽障碍患者管理方案及效果评价

目录

声明
摘要
英文摘要
目录
符号说明：中英文缩略词说明
第一章 前言
第一节 研究背景
1 吞咽障碍是脑卒中后常见的...
2 国内外关于吞咽障碍的研究...
3 国内吞咽障碍管理存在的问题

基于循证构建脑卒中吞咽障碍患者管理方案及效果评价

董小方

郑州大学

在线阅读 整篇下载 分章下载 引用

摘要：研究目的: 掌握脑卒中吞咽障碍患者的真实感受和需求，了解某省神经内科脑卒中吞咽障碍管理现状及医务人员对吞咽障碍管理相关知识和行为的掌握情况。在此基础上，系统检索国内外关于脑卒中吞咽障碍管理的临床实践指南和系统评价，通过专家会议法构建脑卒中吞咽障碍管理最佳实践手册和方案，并进行初步实证研究，以期提供科学、规范、持续的脑卒中吞咽障碍患者方案，促进脑卒中吞咽障碍患者的康复，提高患者的生活质量和诊疗效果。 研究方法: 1.采用质...

关键词：脑卒中; 吞咽障碍; 循证护理; 生活质量

授予学位：博士

[17] 马珂珂,郭园丽,董小方,等. 河南省护士对脑卒中患者吞咽障碍筛查与评估的现状调查. 中华护理杂志,2020,55(8):1196-1200. DOI:10.3761/j.issn.0254-1769.2020.08.016.

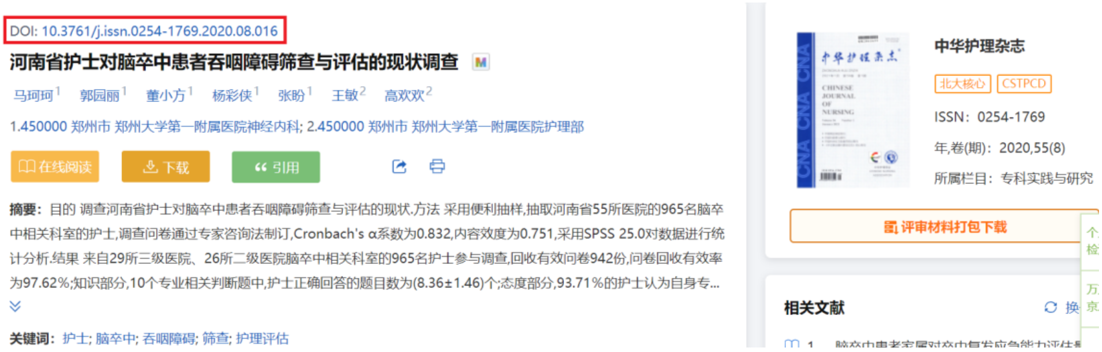

DOI: 10.3761/j.issn.0254-1769.2020.08.016

河南省护士对脑卒中患者吞咽障碍筛查与评估的现状调查

马珂珂[1] 郭园丽[1] 董小方[1] 杨彩侠[1] 张盼[1] 王敏[2] 高欢欢[2]

1.450000 郑州市 郑州大学第一附属医院神经内科; 2.450000 郑州市 郑州大学第一附属医院护理部

在线阅读 下载 引用

摘要：目的 调查河南省护士对脑卒中患者吞咽障碍筛查与评估的现状.方法 采用便利抽样,抽取河南省55所医院的965名脑卒中相关科室的护士,调查问卷通过专家咨询法制订,Cronbach's α系数为0.832,内容效度为0.751,采用SPSS 25.0对数据进行统计分析.结果 来自29所三级医院、26所二级医院脑卒中相关科室的965名护士参与调查,回收有效问卷942份,问卷回收有效率为97.62%;知识部分,10个专业相关判断题中,护士正确回答的题目数为(8.36±1.46)个;态度部分,93.71%的护士认为自身专...

关键词：护士; 脑卒中; 吞咽障碍; 筛查; 护理评估

中华护理杂志

北大核心 CSTPCD

ISSN：0254-1769

年,卷(期)：2020,55(8)

所属栏目：专科实践与研究

评审材料打包下载

相关文献

Reference 15 is a doctoral thesis and reference 16 is a Journal article. The translation of two references are revised as follows:

[16] Dong X. Evidence-based management of stroke patients with dysphagia and its effect evaluation. Zhengzhou University,2018.

[17] Ma K, GUO Y, DONG X, Yang C, Zhang P, Wang M, et al. Investigation on the status of screening and evaluation of dysphagia in stroke patients by nurses in Henan Province. Chinese journal of nursing,2020,55(08):1196-1200. DOI:10.3761/j.issn.0254-1769.2020.08.016.

11. “The MS-KAB-DA was shortened for the Questionnaire of Medical staff’s Knowledge, Attitudes, and Behavior of dysphagia assessment, which is composed of three specific domains: Knowledge, Attitude(eight items), and behavior (13 items). The knowledge domain is composed of 25 items, including 17 True or False items (KR-20=0.760), four single-choice items, and four multiple-choice items.”

So, the total questions were 46, right? In addition, what does “KR-20” mean?

“Based on the suggestion of Hair, Black, Babin, and Anderson[14], a ratio of 10 to 20 participants for each parameter was required for structural equation modeling.”

*The sample size should be 460 (46*10) to 920 (46*20), right?*

Why did the authors mention a sample size of 220 to 440?

The author's answer: "KR-20" is a reliability calculation formula proposed by Kuder-Richardson. The reliability KR-20 is appropriate for True and False items. The total items of this questionnaire were 46, and we have revised as follows[Page 3]:
According to the sample content estimation method proposed by Kendall, the sample content of questionnaire survey should be 5-10 times the number of items. There were 46 items in the questionnaire. Thus, 230-460 participants were calculated according to the ratio. Adding a 15.0% attrition rate, the total participants sample should is between 265 and 529.

12. "Before the study, I obtained the consent of the author of the original questionnaire to modify the questionnaire.
The questionnaire demonstrated good reliability and construct validity; the reported Cronbach's α coefficient was 0.832 –0.879, and the content validity was 0.751 [15-16]."
If the authors modified questions, mentioning the reliability and validity of the old questionnaire is meaningless.

The author's answer: We removed the information about the reliability and validity of the old questionnaire in the article.

13. "Internal consistency estimates were shown to have acceptable reliability for the questionnaire before the survey (Cronbach's alpha=0.865)."
This data was for the authors' questionnaire, right? If yes, I think Cronbach's alpha should be reported separately for each domain (K, A, and B), not all 46 questions.

The author's answer: Yes, this data was for the authors' questionnaire. We have revised the sentence above this question, the details as follows[Page 4]:
Internal consistency estimates were shown to have acceptable reliability for the questionnaire before the survey (Cronbach's alpha for domain K, A, and B are 0.77, 0.97 and 0.91, respectively)

14. "This study was approved by the ethics committee of the study hospital. Data were collected on May 23 –31, 2022. All participants were invited to complete the questionnaire through WeChat, DingTalk, and Tencent QQ from May 23 to May 31, 2022. A professional questionnaire survey platform which provides functions equivalent to Amazon Mechanical Turk called "Wenjuan Xing" was used to investigate. The researcher sent the questionnaire through WeChat, DingTalk, and Tencent QQ to colleagues and classmates to fill in and asked them to forward the questionnaire to their colleagues."

Do not repeat information involving the period of data collection and online platforms. In addition, was the questionnaire designed as a Google Form?

The author's answer: We removed repeat information involving the period of data collection and online platforms. Yes, the questionnaire was designed as a google form, the questionnaire website：https://www.wjx.cn/vm/mc5kOo1.aspx

15. "Univariate analysis (independent-samples t-test and one-way analysis of variance for categorical independent variables) was performed to explore the potential predictors of medical staff KAB for dysphagia assessment."
Did the authors check the normal distribution of data before using the t-test and ANOVA test?

The author's answer: Yes, we checked the normal distribution of data before using the t-test and ANOVA test. The data were normally distributed. It was shown in article that The data were normally distributed by skewness and kurtosis tests[Page 4].

Results

16. Please do not stick the numbers and the brackets. For example, 13(6.16). It should be "13 (6.16)".

The author's answer: We have revised the numbers and the brackets. Please see Tables in the article.

17. Please rename columns in the first row of Table 1.

The author's answer: We have renamed columns in the first row of Table 1.Please see Table 1 for details in the article.

18. Can the authors add some sentences to introduce community nurses?

The author's answer: We have added sentences to introduce community nurses as follows[Page 5]:
12 community nurses were all from Guangdong Province. 10 community nurses were from level 3 hospital, only 2 from level 1 hospital. 7 community nurses (58.3%) had no working experience in the field of dysphagia related diseases and also had no related training for dysphagia.

19. In Table 1, the variable "title" is relatively difficult to understand.

The author's answer: We have revised variable "title" in Table 1. Please see Table 1 for details in the article.

20. A third of participants were not working in the field of dysphagia-related diseases. Why did the authors invite them to participate in this study? It is obvious that knowledge involving dysphagia is unnecessary for them.
Why did the authors not focus on selecting healthcare professionals working in different settings with populations at risk for dysphagia?

The author's answer: The participants of this study included nurses in high-risk departments for swallowing disorders and general nurses. Although one third of the nurses did not work in the

field of swallowing disorders, and they have little knowledge about swallowing disorders. However, with the arrival of the aging society and the high incidence of swallowing disorders, many hospitals require nurses to know the screening of swallowing disorders. In addition, many general nurses in Shenzhen may go to the community for rotation or work. People at risk of swallowing disorders may be encountered in the community.

This study only investigated hospital and community nurses, and did not investigate healthcare professionals in other different Settings such as nursing homes, which is a shortcoming of this study. Limited resources and time make it difficult to organize health care staff in other medical facilities in different Settings to complete the survey. We should focus on the evaluation of dysphagia and provide training for the assessment of swallowing disorders to medical staff in nursing home settings, social health service centers, and palliative care facilities in the future.

21. In Table 2, please explain "percentige rating scores" (how to calculate). Mean/maximum score, right?

This value for the behavior score is inappropriate because the minimum score was 13, not 0.

The author's answer: "percentige rating scores" =Mean*100/Dimensional full score, for example 62.87=15.34*100/24.4. It is a method of standardizing scores by percentage, which facilitates the comparison of scores in various dimensions. This minimum score for the behavior was 13.

22. In Tables 2, 3, 4, and 6, I do not think adding the knowledge, attitude, and behavior scores into a total score is a good idea. These three domains are different.

The author's answer: This study adopted questionnaire survey (***Questionnaire on medical staff's Knowledge, Attitudes and Behavior of dysphagia assessment***), knowledge, attitude and behavior are the dimensions of this questionnaire, and the score of the questionnaire was used as the independent variable in the univariate analysis and multiple regression analysis in this study. These three dimensions are different. Due to the length of the paper, We didn't explained separately, and there are plans to analyze and explain them separately in the future.

23. "As seen in Table 3, medical staff in Shaanxi Province were more willing to assess dysphagia and were evaluated more frequently."

I do not understand this sentence.

In addition, sentences in the "Note" part are difficult to understand.

The author's answer: We have revised the note part in Table 3[Page 7]. One-way ANOVA showed differences in attitudes, behaviors and total questionnaire scores of medical staff in different provinces. Since there were four provinces, post hoc multiple comparisons were made between groups, and the results were written in the "note" part and marked with symbols.

24. Table 5 should be removed or moved to the Supplementary Files. The authors can mention reference groups in Table 6.

For example, related training for dysphagia (ref: No): Yes.

The author's answer: We have moved Table 5 to the supplementary files and revised Table 6 according to your suggestion. Please see details in the article[Page 8-9].

Discussion

25. "This suggests that people are willing to assess swallowing disorders, but lack Knowledge and Behavior. "
This sentence is unclear.

The author's answer: We have revised this sentence as follows[Page 9]:
This showed that the attitude score of medical staff is higher than the behavior score because the standardized score of attitude is 89.7, which is much higher than the standardized score of behavior 63.7.

26. Please add a paragraph involving the strengths and limitations of this study.

The author's answer: We have added a paragraph involving the strengths and limitations of this study as follows[Page 13]:
The strengths and limitations of this study
First, participants in this study were from four provinces in China, showing the differences in knowledge, attitude and behavior of medical staff in different regions in dysphagia assessment. Second, this study explored the influencing factors of medical staff's knowledge, attitude and behavior in tdysphagia assessment, and provided guidance and suggestions for the future organization of relevant training. But, this study only investigated hospital and community nurses, and did not investigate healthcare professionals in other different Settings such as nursing home settings. Future studies should focus on the evaluation of dysphagia and provide training for the assessment of swallowing disorders to medical staff in nursing home settings, social health service centers, and palliative care facilities.

27. Please check the references. I do not know what [J] means.

The author's answer: We have checked the references according to the PLOS ONE guidelines for References. Please see "References" for details in the article[Page 14-17].

28. Please check spelling and grammar mistakes.

The author's answer: We have checked spelling and grammar mistakes, Please see the details in the article.

Thank you for your constructive suggestions.

Reviewer #2:

**Research Paper Title:** Predictors of medical staff's Knowledge, Attitudes, and Behavior of dysphagia assessment: A Cross-Sectional study<br>**Manuscript ID:** PONE-D-23-41544		
**Reviewer 2 comments**<br>This is a very interesting study and was an enjoyable read. Please see specific comments that will add strength and clarity to the study information presented regarding this topic of predictors of medical staff's Knowledge, Attitudes, and Behavior of dysphagia assessment. The manuscript needs proofreading from an agency specialized in this task.		
**Page Number and Line**	**Item to Address**	**Author Response**
Page 1, Author Affiliation	The affiliation between the majority of authors is the Department of Nursing, & the Department of Neurology. It is better to revise & arrange numbers accordingly.	Thank you for your constructive suggestions. We have revised and arranged numbers accordingly [Page 1].

Page 1, Running head	The running head should be briefed:<br>Predictors of medical staff's KAP of dysphagia assessment	Thank you for your constructive suggestions. The running head revised as Predictors of medical staff ' s KAP of dysphagia assessment [Page 1]
Page 1, Abstract	See the author's instructions for PLOS ONE and use the same writing style. In methods & results, an italic style is used together with other styles.	Thank you for your constructive suggestions. We have revised the abstract according to the author's instructions for PLOS ONE and use the same writing style. Please see the details in the article [Page 1-2].
Page 1, Abstract: Methods	Try to avoid abbreviations in the abstract, so write the complete Tencent QQ in the abstract.	We have removed abbreviations in the abstract, "Tencent QQ" is replaced by "Tencent instant messenger" [Page 1]
Page 1, Abstract: Results	• In the results put the average & SD within brackets and connect them with (Mean ±SD).<br>• To be more organized & not confusing to the reader, I think it's better to write ONE digit only after the decimal point and this would be applied to all results within context & tables.<br>• Avoid using "&" in writing the manuscript, use and instead of it.<br>• In the last line of the results of the abstract be careful for s after the apostrove and don't keep space.	Thank you for your constructive suggestions. We have revised according to your suggestions, Please see the details in the article [Page 1-2].
Page 1, Abstract: Conclusions	Try to avoid first, second, and third singular or plural in objective or possessive cases while writing the manuscript. Revise the whole manuscript & change accordingly. In this case, you can write (The study findings instead of Our findings).	Thank you for your constructive suggestions. We have revised the whole manuscript & change according to your suggestions, Please see the details in the article.

Page 2, Keywords	It is better to write keywords in alphabetical.	Key words: assessment, Attitude, Behavior, Cross-Sectional, dysphagia, Knowledge, Predictors, stroke
Page 2, Introduction<br><br><br><br><br>Page 2, last 4 lines of Introduction	• Write a decimal with one digit for each percentage to have consistency for all numbers & this would be applied to all results within the whole context & tables of this study.<br>• Consistency should be also for writing the whole number by adding 0 digits after decimal 90.0%. This could be applied to all numbers within the study.<br>• Further elaboration should be mentioned before aiming that focus on the rationale for conducting this study in China, and previous studies that dealt with this issue in worldwide countries if no study in China deals with knowledge, attitude, and behavior of dysphagia assessment.	We have revised each percentage with one digit within the whole context and table of this study.<br><br>We have mentioned further elaboration as follows [Page 3]:<br>Studies on the factors contributing to barriers to the assessment of dysphagia are lacking. Some Chinese researchers investigated the practice and management of medical staff's dysphagia assessment, it showed that More than 96.0% of the nurses did not know anything about the swallowing function training, and only 36.7% of the nurses used the scale to screen the patients for swallowing disorders[14]. And these studies did not explore the influencing factors. We aimed to explore......<br>[14] Zhang Y, Liu Y, Li W, Lin Y. Investigation on the knowledge and intervention status of swallowing function training in elderly patients by nurses in Guangzhou and Shenzhen. Journal of Nurses Training, 2018,33(16):1499-1500,1531.DOI:10.16821/j.cnki.hsjx.2018.16.021.
Page 2-4, Methods	There are a lot of issues that have to be addressed in the methods section as the following:<br>• It is unclear if the researcher added a few questions to the original instrument & checked for external validity. How questionnaire was administered in English or Chinese language?<br>• What is about reliability & validity of the	We have addressed in the methods section as the following [Page 4]:<br>Before the study, we obtained the consent of the author of the original questionnaire to add the scoring method and paraphrase the items of knowledge dimension on the basis of the original questionnaire......Internal consistency estimates were shown to have acceptable reliability for the questionnaire before the survey (Cronbach’s alpha for domain K, A, and B are 0.77, 0.97 and 0.91,

different tools used in this study?

- The researcher mentioned knowledge, and attitudes (8 items), while at the end of page 3, the knowledge domain is composed of 25 items that will be confusing for the reader.
- Scoring is not clear & confused also. Explain it clearly.
- It is better to write the parts of the research instrument as “The following instrument covered two parts a) The general information sheet…….., and b) Assessment of knowledge, attitude, and behavior of dysphagia.
- Why convenience sampling approach was used in this study? To generalize findings it is better to utilize probability approach rather than non-probability. For this study the proper sampling is cluster.
- What does it mean by “a questionnaire that completed less than 300 s and more than 3600 s”? It is not clear.
- As mentioned before avoid using the pronoun as the third person in the manuscript & revise it again to change accordingly.
- There is no need for repetition of ethical approval when data is collected, & naming the four provinces. The first two sentences and the

respectively). The instrument was administered in Chinese language and designed as a google form, the website was https://www.wjx.cn/vm/mc5kOo1.aspx.

The researcher sent the questionnaire website through WeChat, DingTalk, and Tencent instant messenger to colleagues and classmates to fill in and asked them to forward the questionnaire to their colleagues.

We have revised Questionnaire introduction (domain, items and scoring), please see the details in the article. And we have added a Supporting information as follows [Page 4, 13-14]:

S1 Table. The scoring methods and rules of the questionnaire. (DOC)

Table 1 The scoring methods and rules of the questionnaire

Domains	Items	n	scoring methods and rules
Knowledge	True or False questions	17	1 point is scored for correct answer. 0 point is scored for incorrect answer.
	Single-choice questions	4	
	Multiple-choice questions	4	Count 0.2/ option when selecting the correct option. Count 0 when the wrong option was selected or the correct and wrong options were selected simultaneously<br>For example, a multiple-choice question had three correct choices that score 0.6 when participant selected three correct choices.
Attitudes	Likert-five-point-scale	8	Likert 5-level scoring method. Ranging from “strongly agreed” to “strongly disagreed”. 5 point for “strongly agreed”, 1 point for “strongly disagreed”
Behavior	Likert-five-point-scale	13	Likert 5-level scoring method. Ranging from “the most frequently” to “the least frequently. 5point for “the most frequently”, 1 point for “the least frequently

	last line in the data collection and analysis subheading are to be deleted. Start the paragraph from “All participants……..among FOUR provinces.<br>• Instead of “n” in the example of descriptive statistics, it is better to write frequency. Descriptive statistics (e.g., Mean ±SD, frequency & %).	We have rewritten the parts of the research instrument according your suggestions [Page 3-4].<br><br>It is difficult to utilize probability approach because of large sample size and area. So we used convenience sampling.<br><br>“a questionnaire that completed less than 300 s and more than 3600 s” means the time to fill in the questionnaire is shorter than 300 seconds and longer than 3600 seconds. We have revised the sentence [Page 3].<br>According to the test, it took 300 to 1800 seconds (5-30 minutes) to fill out a questionnaire. In consideration of the possibility of interruptions during the filling process, an additional 30 minutes was added. If it takes less than 5 minutes to fill in the questionnaire, it is likely that the person who filled in the questionnaire did not read the question carefully and filled in randomly<br>We have checked the paper throughout for avoiding using the pronoun as the third person in the manuscript.<br>We have removed the description of ethical approval and naming the four provinces in the data section. And the first two sentences and the last line in the data collection and analysis subheading had been deleted [Page 4].<br>We have revised as follows [Page 4]:<br>Descriptive statistics (e.g., Mean ±SD, frequency & %) were......

Page 4-9: Results	There are a lot of issues that have to be addressed in this section:<br>• Numbers within tables have to be changed into one digit after the decimal point like this 13(6.2) in the first row and be careful to have 100 % to the total of each column.<br>• For table 1 the total number is 328 (211+77+40=328). A total of 25 subjects are missed. What about the demographic characteristics of the missed province (Guangxi Province)? Rewrite the table again & revise the number & percentage for each province. Calculation of F, t. Mean has to consider the missed province. Check again your SPSS to ensure the missed one is included in all calculations.<br>• Correct the spelling mistakes of a few words within tables & whole manuscript. As I suggested this manuscript needs proofreading from one agency expert in this field.<br>• Use (Mean ±SD) for tables 2 & 3 to distinguish them from other tables of N (%).<br>• For each variable in the first column of all tables write the first letter as capital (Hospital level- Level 1 hospital etc…). Be	We have revised the Tables according your suggestions, Please see the details in the article.<br>We have checked my SPSS and rewritten the Table 1 and revised the number & percentage for each province (including Guangxi Province). Please see the Table 1 for details in the article [Page 5-6].<br><br>We have checked the manuscripts and forms for spelling errors.<br><br>We have revised (Mean ±SD) for tables 2 & 3 [Page 6-7].<br><br>We have revised table1-6 according to PLOS ONE Table Guidelines. Please see the details in the article.<br><br>We have revised heading according to PLOS ONE Heading Guidelines. Please see the details in the article.

	consistent in writing & use the same style. Don't use two styles within the same sentence & be careful with font size also. As seen in Table 3, medical staff in Shaanxi Province This would be applied for all tables from 1-6.<br>• Subheadings should not be in the same font and style. This should be applied to all sections.	
Page 9-12: Discussion	• The author can not say in our study (In this study).<br>• Notes mentioned before regarding percentages & consistency in writing styles should applied in this section.<br>• In discussion, the author can say findings are consistent or contradict previous studies. Try to utilize these in the discussion section to show similarities & differences within the study conducted & give a rationale for your results.<br>• I grasp from the discussion, that few studies were conducted in China that are similar to this study. The authors have to explain the reasons for conducting this study, particularly in four provinces. Further elaboration had to be discussed in the	We have replaced "in our study" with "in this study".<br>We have checked discussion section for percentages & consistency in writing styles.<br><br>We have added a description of the comparison with other studies and explained the reasons for similarities & differences in the first paragraph of the discussion.As follows [Page 9]:<br>The results of this study are different from study of Sun Qian et al[19]. The percentige rating score of knowledge and attitude in this study is similar to study of Sun Qian, which is at the medium and high level respectively. However, the percentige rating score of behavior in this study is slightly lower than study of Sun Qian (63.7 VS 71.0). The reasons may be as follows: Firstly, different participants and locations were surveyed in two studies. Sun Qian surveyed nurses in 17 Grade III hospitals in Beijing, while this study surveyed medical staff in hospitals of different levels in four provinces. Secondly, different departments were surveyed in two studies. Sun Qian only investigated the department of neurosurgery, which

	introduction section as mentioned before.<br>• The discussion section needs to be organized well & deal with the recent studies to compare and interpret study findings.	is known to pay more attention to the evaluation practice of swallowing disorders than other departments.<br>[19] Sun Q, Su Y, Wang Y.Investigation on the current situation of knowledge and practice in nursing of dysphagia after stroke in neurology nurses. Chinese journal of modern nursing,2020,26(23):3144-3150. DOI:10.3760/cma.j.cn115682-20200322-02063.<br>We have added a description of explaining the reasons for conducting this study as follows [Page 9]:<br>The knowledge of medical staff on dysphagia assessment directly affects the quality of care and the prognosis of patients. However, most nurses lack the knowledge of dysphagia assessment, which may be related to lack of relevant training and attention paid by medical institutions to the knowledge education of nurses[18]. To build an appropriate and effective training program, it is necessary to combine the influencing factors of nurses' knowledge, attitude and behavior in the assessment of swallowing disorders. There is no research on the influencing factors of knowledge, attitude and behavior of dysphagia assessment in China. This study was conducted in multiple regions (4 provinces), multiple populations (doctors, clinical and community nurses, technicians, etc.) and multiple departments (neurosurgery and general departments) aiming to find as different data and features as possible to serving the training program.<br>[18] Lv S, Zhou G, Mao Y, Zhang Y, Wang J. Research progress on the attitude, knowledge and behavior of nurses and caregivers for swallowing disorders after stroke in China. Chinese General practice nursing,2023,21(1):37-41. DOI:10.12104/j.issn.1674-4748.2023.01.009.

		Discussion section have been organized.
Page 12-13: Conclusion	• Be specific in your conclusion of this study. Nothing is mentioned about the conclusion in that section. Revise this section & add a conclusion.	We have added specific conclusion as follows [Page 13]:<br>Conclusion<br>The attitude of Chinese medical staff to the dysphagia assessment is positive, but the knowledge and behavior needs to be improved, and the attitude is separated from the knowledge and the behavior.This study findings also imply that nursing experience, training, and work for patients with swallowing disorders could have positive effects on the Knowledge, Attitudes and Behavior of medical staff regarding dysphagia assessment. The medical management department should take various forms to train the knowledge and skills related to the evaluation of swallowing disorders. Hospital administrators should provide relevant resources, such as videos of dysphagia assessment, training centers for the assessment of dysphagia, and swallowing specialist nurses. It is important that health policies fully recognize the role of training and support systems in caring for people with dysphagia.
Page 13-16: References	• The first reference- Johnson, C. O., Nguyen, M., Roth, G. A., et al. (2019). Global, regional, and national burden of stroke, 1990– 2016: a systematic analysis for the Global Burden of Disease Study 2016. Lancet Neurology, 18(5), 439–458. https://doi.org/10.1016/S1474-4422(19)30034-1 is different from the second one.<br>• Check again reference # 32 is different	Thank you for your constructive suggestions. We have revised references according your suggestions and PLOS ONE guidelines for References, and checked spelling errors in References section. Please see the details in the article.<br><br>We have re-searched the recent relevant literature and replaced some old literature. Some older literature were not replaced because there were important data or authoritative theories citations [Page 14-17].

	from the previous one<br>• Authors exceeding three or more authors should be written with et al at the end, but this is not applied for all references as in the first reference. Compare the first reference with the second & third one as an example to see the difference.<br>• Journal abbreviation should be as in index medicus & pay attention to be written with dots in between or without (Ann Med Res) and consistency in written abbreviation.<br>• Ten out of thirty-six references were old. Why? I hope to add more recent citations in the introduction & discussion sections instead of these old references.<br>• I request to revise all references and pay attention to PLOS ONE author guidelines.<br>• There are a lot of spelling errors in this section. Revise & correct them.	
