# Supplementary material for: Predictors of medical staff’s knowledge, attitudes and behavior of dysphagia assessment: A cross-sectional study
Source: PLoS One. 2024 Apr 5;19(4):e0301770. doi: 10.1371/journal.pone.0301770 (PMC10997058; doi:10.1371/journal.pone.0301770)
Supplement: Attachment — Submitted filename: Comment to the authors.docx [file pone.0301770.s007.docx]

Thank the editors for inviting me to peer-review this manuscript. The topic is relatively meaningful and exciting. However, there are a lot of issues that need to be corrected before publication. I have some comments and hope that they are helpful to the authors.

**Abstract**

1. The objective is unclear (too large). In addition, in the Purpose and Introduction, the authors mentioned “stroke”. However, I do not know whether or not “stroke” was a factor associated with the inclusion criteria of participants.

2. The authors should not mention the number of medical staff approached (430 nurses and doctors) in the Methods. The final number of participants should be mentioned (n=353).

**Introduction**

3. The authors wrote: “Dysphagia occurs in 37–78% of patients after stroke [2-4].”

However, these three references are seemingly not relevant to the sentence above.

4. The Introduction is quite long. I think the authors should divide it into two paragraphs (one for the prevalence of dysphagia and one for studies involving their topic + the objectives).

**Methods**

5. What does “Chinese RNs” mean?

6. “Based on the suggestion of Hair, Black, Babin, and Anderson[14], a ratio of 10 to 20 participants for each parameter was required for structural equation modeling.”

Please check the reference 14.

7. “Thus, 220–440 participants were followed by a calculation of a 15% attrition rate, which resulted in a total sample of 430 participants in this study.”

This sentence is unclear.

8. “An anonymous survey was conducted with permission from the research ethics committee of the participating hospital.”

This sentence is unclear (the participating hospital).

In addition, please do not repeat the information involving the ethics in the Methods. The authors had an “Ethical statements” part already.

(“Data collection and Analysis

This study was approved by the ethics committee of the study hospital.”)

9. “The inclusion criteria were as follows: (a) Chinese RNs, doctors, or therapists with a medical background, (b) working at the hospital for more than 3 months, and (c) not internship.”

I do not know how the authors identified and approached 430 nurses and doctors. The authors used online platforms to collect data. However, the participants only came from four provinces (Guangdong Province, Hunan Province, Guangxi Province, and Shaanxi Province).

10. “Before the study, I obtained the consent of the author of the original questionnaire to modify the questionnaire. This questionnaire was revised based on the existing questionnaires designed by Dr. Dong Xiaofang[15] and Master Ma Keke[16] from The First Affiliated Hospital of Zhengzhou University.”

First, “I” should be replaced by “we”.

Second, the authors mentioned two references for their questionnaire. However, the reference 15 cannot be identified. Is it a thesis?

The reference 16 cannot be found in Google/PubMed.

Can the authors attach the questionnaire as a supplementary file?

11. “The MS-KAB-DA was shortened for the Questionnaire of Medical staff’s Knowledge, Attitudes, and Behavior of dysphagia assessment, which is composed of three specific domains: Knowledge, Attitude(eight items), and behavior (13 items). The knowledge domain is composed of 25 items, including 17 True or False items (KR-20=0.760), four single-choice items, and four multiple-choice items.”

So, the total questions were 46, right? In addition, what does “KR-20” mean?

“Based on the suggestion of Hair, Black, Babin, and Anderson[14], a ratio of 10 to 20 participants for each parameter was required for structural equation modeling.”

The sample size should be 460 (46*10) to 920 (46*20), right?

Why did the authors mention a sample size of 220 to 440?

12. “Before the study, I obtained the consent of the author of the original questionnaire to modify the questionnaire.

The questionnaire demonstrated good reliability and construct validity; the reported Cronbach's α coefficient was 0.832–0.879, and the content validity was 0.751 [15-16].”

If the authors modified questions, mentioning the reliability and validity of the old questionnaire is meaningless.

13. “Internal consistency estimates were shown to have acceptable reliability for the questionnaire before the survey (Cronbach’s alpha=0.865).”

This data was for the authors’ questionnaire, right? If yes, I think Cronbach’s alpha should be reported separately for each domain (K, A, and B), not all 46 questions.

14. “This study was approved by the ethics committee of the study hospital. Data were collected on May 23–31, 2022. All participants were invited to complete the questionnaire through WeChat, DingTalk, and Tencent QQ from May 23 to May 31, 2022. A professional questionnaire survey platform which provides functions equivalent to Amazon Mechanical Turk called “Wenjuan Xing” was used to investigate. The researcher sent the questionnaire through WeChat, DingTalk, and Tencent QQ to colleagues and classmates to fill in and asked them to forward the questionnaire to their colleagues.”

Do not repeat information involving the period of data collection and online platforms. In addition, was the questionnaire designed as a Google Form?

15. “Univariate analysis (independent-samples t-test and one-way analysis of variance for categorical independent variables) was performed to explore the potential predictors of medical staff KAB for dysphagia assessment.”

Did the authors check the normal distribution of data before using the t-test and ANOVA test?

**Results**

16. Please do not stick the numbers and the brackets. For example, 13(6.16). It should be “13 (6.16)”.

17. Please rename columns in the first row of Table 1.

18. Can the authors add some sentences to introduce community nurses?

19. In Table 1, the variable “title” is relatively difficult to understand.

20. A third of participants were not working in the field of dysphagia-related diseases. Why did the authors invite them to participate in this study? It is obvious that knowledge involving dysphagia is unnecessary for them.

Why did the authors not focus on selecting healthcare professionals working in different settings with populations at risk for dysphagia?

21. In Table 2, please explain “percentige rating scores” (how to calculate). Mean/maximum score, right?

This value for the behavior score is inappropriate because the minimum score was 13, not 0.

22. In Tables 2, 3, 4, and 6, I do not think adding the knowledge, attitude, and behavior scores into a total score is a good idea. These three domains are different.

23. “As seen in Table 3, medical staff in Shaanxi Province were more willing to assess dysphagia and were evaluated more frequently.”

I do not understand this sentence.

In addition, sentences in the “Note” part are difficult to understand.

24. Table 5 should be removed or moved to the Supplementary Files. The authors can mention reference groups in Table 6.

For example, related training for dysphagia (ref: No): Yes.

**Discussion**

25. “This suggests that people are willing to assess swallowing disorders, but lack Knowledge and Behavior.”

This sentence is unclear.

26. Please add a paragraph involving the strengths and limitations of this study.

27. Please check the references. I do not know what [J] means.

28. Please check spelling and grammar mistakes.

Best wishes to the authors.
