# Supplementary material for: Predictors of medical staff’s knowledge, attitudes and behavior of dysphagia assessment: A cross-sectional study
Source: PLoS One. 2024 Apr 5;19(4):e0301770. doi: 10.1371/journal.pone.0301770 (PMC10997058; doi:10.1371/journal.pone.0301770)
Supplement: Attachment — Submitted filename: Response to Reviewers.doc [file pone.0301770.s010.doc]

Response to reviewers

Dear editors and reviewers:

Thank you for your letter and for the reviewer’s comments concerning our manuscript entitled “Predictors of medical staff’s Knowledge, Attitudes and Behavior of dysphagia assessment: A Cross-Sectional study”(ID:PONE-D-23-41544). Those comments are all valuable and very helpful for revising and improving our paper, as well as the important guiding significance to our researches. We have studied comments carefully and have made correction which we hope meet with approval.

We submited four items as follow:

1. Response to Reviewers
2. Revised manuscript with track changes
3. Manuscript (revised)
4. updated cover letter

Below, please find a response to each of the reviewer’s comments. We have revised manuscript with track changes. We hope that the revisions in the manuscript and our accompanying responses will meet editors’ and reviewers’ criteria.

We shall look forward to hearing from you at your earliest convenience.

Yours sincerely

Juanhui Chen

**Acdemic editor**

*1. We note that the grant information you provided in the ‘Funding Information’ and ‘Financial Disclosure’ sections do not match.*

*When you resubmit, please ensure that you provide the correct grant numbers for the awards you received for your study in the ‘Funding Information’ section.*

**The author’s answer:** When we resubmit, we have provided the correct grant number(No. LGKCYLWS2020128) in ‘Funding information’ section.

Funding: Medical and Health Technology Plan of Longgang District, Shenzhen. Funding was rewarded to Juanhui Chen. The Grant award number was LGKCYLWS2020128. URL: <http://www.lg.gov.cn/bmzz/kjj/xxgk/qt/tzgg/content/post_7970194.html> The funders play no role in the study design, data collection and analysis, decision to publish, or preparation of the manuscript.

*2. Thank you for stating the following in the Acknowledgments Section of your manuscript:*

*"We would like to thank Shenzhen Longgang District Science and Technology*

*Innovation Bureau for providing the Unfunded Medical and Health Technology*

*Plan(No. LGKCYLWS2020128)"*

*We note that you have provided funding information that is not currently declared in your Funding Statement. However, funding information should not appear in the Acknowledgments section or other areas of your manuscript. We will only publish funding information present in the Funding Statement section of the online submission form.*

*Please remove any funding-related text from the manuscript and let us know how you would like to update your Funding Statement. Currently, your Funding Statement reads as follows:*

*"The author(s) received no specific funding for this work"*

*Please include your amended statements within your cover letter; we will change the online submission form on your behalf.*

**The author’s answer:** We have removed any funding-related text from the manuscript, and amended financial disclosure statement whthin our cover letter as follows:

**Financial disclosure statement**:

Medical and Health Technology Plan of Longgang District, Shenzhen.

Funding was rewarded to Juanhui Chen. The Grant award number was LGKCYLWS2020128. URL: http://www.lg.gov.cn/bmzz/kjj/xxgk/qt/tzgg/content/post_7970194.html The funders play no role in the study design, data collection and analysis, decision to publish, or preparation of the manuscript.

*3. When completing the data availability statement of the submission form, you indicated that you will make your data available on acceptance. We strongly recommend all authors decide on a data sharing plan before acceptance, as the process can be lengthy and hold up publication timelines. Please note that, though access restrictions are acceptable now, your entire data will need to be made freely accessible if your manuscript is accepted for publication. This policy applies to all data except where public deposition would breach compliance with the protocol approved by your research ethics board. If you are unable to adhere to our open data policy, please kindly revise your statement to explain your reasoning and we will seek the editor's input on an exemption. Please be assured that, once you have provided your new statement, the assessment of your exemption will not hold up the peer review process.*

**The author’s answer:** We have built a dataset to share data on Dryad. The DOI URL is <https://doi.org/10.5061/dryad.djh9w0w70.>

The temporary URL:

<https://datadryad.org/stash/share/GQq3KacnFwuVnq8aE-fIQmF62FN5xn08L7rAYaFIyoQ>

**Data Availability Statement**: Data is deposited at Dryad

(DOI: https://doi.org/10.5061/dryad.djh9w0w70).

**Reviewer #1**:

*Abstract*

1. *The objective is unclear (too large). In addition, in the Purpose and Introduction, the authors mentioned “stroke”. However, I do not know whether or not “stroke” was a factor associated with the inclusion criteria of participants.*

**The author’s answer:** “Stoke” was not a factor associated with the inclusion criteria of participants. We have revised the objective. The revised objective as follows [Page 1]:

This study aimed to explore the predictors of medical staff’s Knowledge, Attitudes and Behavior of dysphagia assessment, to provide reference suggestions for constructing the training program and improving the rate of dysphagia assessment.

*2. The authors should not mention the number of medical staff approached (430 nurses and doctors) in the Methods. The final number of participants should be mentioned (n=353).*

**The author’s answer:** We have revised the number of medical staff in method as follows[Page 1]:

Methods: This study was a cross-sectional study. A total of 353 nurses and doctors from four provinces(Guangdong, Hunan, Guangxi, and Shaanxi) who were selected by convenience sampling......

*Introduction*

*3. The authors wrote: “Dysphagia occurs in 37–78% of patients after stroke [2-4].”*

*However, these three references are seemingly not relevant to the sentence above.*

**The author’s answer:** We have re-searched the relevant literature and revised sentence in the introduction is “*Dysphagia occurs in 30–78% of patients after stroke [2-4]*”. Changed References are as follows[Page 2, 14]:

[2] Vilardell N, Rofes L, Nascimento WV, Muriana D, Palomeras E, Clavé P. Cough reflex attenuation and swallowing dysfunction in sub-acute post-stroke patients: prevalence, risk factors, and clinical outcome. Neurogastroenterol Motil. 2017 Jan;29(1). doi: 10.1111/nmo.12910. Epub 2016 Jul 18. PMID: 27424849.

It is shown in reference 2 that “We included 225 PSP. Prevalence of impaired CRT was 5.8%, that of OD was 40.4% (20.4% with impaired safety of swallow), and of both impairments was, 1.8%”.

[3] Matsumura T, Mitani Y, Oki Y, Fujimoto Y, Ishikawa A. Risk factors for the onset of aspiration pneumonia among stroke patients in the recovery stage. Nihon Ronen Igakkai Zasshi. 2014;51(4):364-8.doi: 10.3143/geriatrics.51.364. PMID: 25327371.

It is shown in reference 3 that “The study subjects included 76 stroke patients (mean age, 74.7±8.4 years) with dysphagia chosen from 175 stroke patients who were newly admitted to four rehabilitation hospitals”. According to the above data, the incidence of dysphagia in stroke patients was 43.43%.

[4] Finnegan BS, Meighan M M, Warren NC, Hatfield MK, Alexeeff S, Lipiz J, et al. Validation Study of Kaiser Permanente Bedside Dysphagia Screening Tool in Acute Stroke Patients. Perm J. 2020 Dec;24:1. doi: 10.7812/TPP/19.230. PMID: 33482958; PMCID: PMC7849282.

It is shown in reference 4 that “Difficulty swallowing or dysphagia is common among acute

stroke patients, occurring in approximately 30% to 78% of the population”.

*4. The Introduction is quite long. I think the authors should divide it into two paragraphs (one for the prevalence of dysphagia and one for studies involving their topic + the objectives).*

**The author’s answer:** The introduction has been divided into three paragraphs. Please see detail in the article[Page 2-3].

*Methods*

*5. What does “Chinese RNs” mean?*

**The author’s answer:** Chinese RNs” mean Chinese nurse practitioners. We have revised in the article[Page 3].

*6. “Based on the suggestion of Hair, Black, Babin, and Anderson[14], a ratio of 10 to 20 participants for each parameter was required for structural equation modeling.”*

*Please check the reference 14.*

**The author’s answer:** We have re-searched the literature on sample size estimation, according to the sample content estimation method proposed by Kendall, the sample content of questionnaire survey should be 5-10 times the number of items.

The revised references is as follows[Page 15]:

[15] Kendall MG. kendall's advanced theory of statistics. Halsted Press; Edward Arnold, 2004.

*7. “Thus, 220–440 participants were followed by a calculation of a 15% attrition rate, which resulted in a total sample of 430 participants in this study.”*

*This sentence is unclear.*

**The author’s answer:** This sentence has been revised as follows[Page 3]:

According to the sample content estimation method proposed by Kendall, the sample content of questionnaire survey should be 5-10 times the number of items. There were 46 items in the questionnaire. Thus, 230-460 participants were calculated according to the ratio. Adding a 15.0% attrition rate, the total participants sample should between 265 and 529. A total of 353 nurses and doctors completed the survey in this study.

*8. “An anonymous survey was conducted with permission from the research ethics committee of the participating hospital.”*

*This sentence is unclear (the participating hospital).*

*In addition, please do not repeat the information involving the ethics in the Methods. The authors had an “Ethical statements” part already.*

*(“Data collection and Analysis*

*This study was approved by the ethics committee of the study hospital.”)*

**The author’s answer:** We removed the information involving the ethics in the Data collection and Analysis. The sentence in the question above has been revised as follows[Page 3]:

An anonymous survey was conducted by Longgang central hospital of Shenzhen on May 23-31, 2022.

*9. “The inclusion criteria were as follows: (a) Chinese RNs, doctors, or therapists with a medical background, (b) working at the hospital for more than 3 months, and (c) not internship.”*

*I do not know how the authors identified and approached 430 nurses and doctors. The authors used online platforms to collect data. However, the participants only came from four provinces (Guangdong Province, Hunan Province, Guangxi Province, and Shaanxi Province).*

**The author’s answer:** Firstly, there are instructions at the beginning of the questionnaire that including The inclusion criteria. Secondly, We analyzed the data of departments, titles and positions and so on filled in by the research participants for judging whether they meet the the inclusion criteria.

*10. “Before the study, I obtained the consent of the author of the original questionnaire to modify the questionnaire. This questionnaire was revised based on the existing questionnaires designed by Dr. Dong Xiaofang[15] and Master Ma Keke[16] from The First Affiliated Hospital of Zhengzhou University.”*

*First, “I” should be replaced by “we”.*

*Second, the authors mentioned two references for their questionnaire. However, the reference 15 cannot be identified. Is it a thesis?*

*The reference 16 cannot be found in Google/PubMed.*

*Can the authors attach the questionnaire as a supplementary file?*

**The author’s answer:** “I” has been replaced by “we”in the article[Page 4]. The questionnaire has been submitted as a supplementary file. References 15 and 16 were came from Chinese researchers, and format of this Chinese references is as follows:

[16] 董小方. 基于循证构建脑卒中吞咽障碍患者管理方案及效果评价[D]. 河南:郑州大学,2018.

[17] 马珂珂,郭园丽,董小方,等. 河南省护士对脑卒中患者吞咽障碍筛查与评估的现状调查. 中华护理杂志,2020,55(8):1196-1200. DOI:10.3761/j.issn.0254-1769.2020.08.016.

Reference 15 is a doctoral thesis and reference 16 is a Journal article. The translation of two references are revised as follows:

[16] Dong X. Evidence-based management of stroke patients with dysphagia and its effect evaluation. Zhengzhou University,2018.

[17] Ma K, GUO Y, DONG X, Yang C, Zhang P, Wang M, et al. Investigation on the status of screening and evaluation of dysphagia in stroke patients by nurses in Henan Province. Chinese journal of nursing,2020,55(08):1196-1200. DOI:10.3761/j.issn.0254-1769.2020.08.016.

*11. “The MS-KAB-DA was shortened for the Questionnaire of Medical staff’s Knowledge, Attitudes, and Behavior of dysphagia assessment, which is composed of three specific domains: Knowledge, Attitude(eight items), and behavior (13 items). The knowledge domain is composed of 25 items, including 17 True or False items (KR-20=0.760), four single-choice items, and four multiple-choice items.”*

*So, the total questions were 46, right? In addition, what does “KR-20” mean?*

*“Based on the suggestion of Hair, Black, Babin, and Anderson[14], a ratio of 10 to 20 participants for each parameter was required for structural equation modeling.”*

*The sample size should be 460 (46*10) to 920 (46*20), right?*

*Why did the authors mention a sample size of 220 to 440?*

**The author’s answer:***“*KR-20” is a reliability calculation formula proposed by Kuder-Richardson. The reliability KR-20 is appropriate for True and False items. The total items of this questionnaire were 46, and we have revised as follows[Page 3]:

According to the sample content estimation method proposed by Kendall, the sample content of questionnaire survey should be 5-10 times the number of items. There were 46 items in the questionnaire. Thus, 230-460 participants were calculated according to the ratio. Adding a 15.0% attrition rate, the total participants sample should is between 265 and 529.

*12. “Before the study, I obtained the consent of the author of the original questionnaire to modify the questionnaire.*

*The questionnaire demonstrated good reliability and construct validity; the reported Cronbach's α coefficient was 0.832–0.879, and the content validity was 0.751 [15-16].”*

*If the authors modified questions, mentioning the reliability and validity of the old questionnaire is meaningless.*

**The author’s answer:** We removed the information about the reliability and validity of the old questionnaire in the article.

*13. “Internal consistency estimates were shown to have acceptable reliability for the questionnaire before the survey (Cronbach’s alpha=0.865).”*

*This data was for the authors’ questionnaire, right? If yes, I think Cronbach’s alpha should be reported separately for each domain (K, A, and B), not all 46 questions.*

**The author’s answer:** Yes, this data was for the authors’ questionnaire. We have revised the sentence above this question, the details as follows[Page 4]:

Internal consistency estimates were shown to have acceptable reliability for the questionnaire before the survey (Cronbach’s alpha for domain K, A, and B are 0.77, 0.97 and 0.91, respectively)

*14. “This study was approved by the ethics committee of the study hospital. Data were collected on May 23–31, 2022. All participants were invited to complete the questionnaire through WeChat, DingTalk, and Tencent QQ from May 23 to May 31, 2022. A professional questionnaire survey platform which provides functions equivalent to Amazon Mechanical Turk called “Wenjuan Xing” was used to investigate. The researcher sent the questionnaire through WeChat, DingTalk, and Tencent QQ to colleagues and classmates to fill in and asked them to forward the questionnaire to their colleagues.”*

*Do not repeat information involving the period of data collection and online platforms. In addition, was the questionnaire designed as a Google Form?*

**The author’s answer:** We removed repeat information involving the period of data collection and online platforms. Yes, the questionnaire was designed as a google form, the questionnaire website：<https://www.wjx.cn/vm/mc5kOo1.aspx>

*15. “Univariate analysis (independent-samples t-test and one-way analysis of variance for categorical independent variables) was performed to explore the potential predictors of medical staff KAB for dysphagia assessment.”*

*Did the authors check the normal distribution of data before using the t-test and ANOVA test?*

**The author’s answer:** Yes, we checked the normal distribution of data before using the t-test and ANOVA test. The data were normally distributed. It was shown in article that The data were normally distributed by skewness and kurtosis tests[Page 4].

*Results*

*16. Please do not stick the numbers and the brackets. For example, 13(6.16). It should be “13 (6.16)”.*

**The author’s answer:** We have revised the numbers and the brackets. Please see Tables in the article.

17. *Please rename columns in the first row of Table 1.*

**The author’s answer:** We have renamed columns in the first row of Table 1.Please see Table 1 for details in the article.

*18. Can the authors add some sentences to introduce community nurses?*

**The author’s answer:** We have added sentences to introduce community nurses as follows[Page 5]:

12 community nurses were all from Guangdong Province. 10 community nurses were from level 3 hospital, only 2 from level 1 hospital. 7 community nurses (58.3%) had no working experience in the field of dysphagia related diseases and also had no related training for dysphagia.

*19. In Table 1, the variable “title” is relatively difficult to understand.*

**The author’s answer:** We have revised variable “title” in Table 1. Please see Table 1 for details in the article.

*20. A third of participants were not working in the field of dysphagia-related diseases. Why did the authors invite them to participate in this study? It is obvious that knowledge involving dysphagia is unnecessary for them.*

*Why did the authors not focus on selecting healthcare professionals working in different settings with populations at risk for dysphagia?*

**The author’s answer:** The participants of this study included nurses in high-risk departments for swallowing disorders and general nurses. Although one third of the nurses did not work in the field of swallowing disorders, and they have little knowledge about swallowing disorders. However, with the arrival of the aging society and the high incidence of swallowing disorders, many hospitals require nurses to know the screening of swallowing disorders. In addition, many general nurses in Shenzhen may go to the community for rotation or work. People at risk of swallowing disorders may be encountered in the community.

This study only investigated hospital and community nurses, and did not investigate healthcare professionals in other different Settings such as nursing homes, which is a shortcoming of this study. Limited resources and time make it difficult to organize health care staff in other medical facilities in different Settings to complete the survey. We should focus on the evaluation of dysphagia and provide training for the assessment of swallowing disorders to medical staff in nursing home settings, social health service centers, and palliative care facilities in the future.

*21. In Table 2, please explain “percentige rating scores” (how to calculate). Mean/maximum score, right?*

*This value for the behavior score is inappropriate because the minimum score was 13, not 0.*

**The author’s answer: “**percentige rating scores” =Mean*100/Dimensional full score, for example 62.87=15.34*100/24.4. It is a method of standardizing scores by percentage, which facilitates the comparison of scores in various dimensions. This minimum score for the behavior was 13.

*22. In Tables 2, 3, 4, and 6, I do not think adding the knowledge, attitude, and behavior scores into a total score is a good idea. These three domains are different.*

**The author’s answer:** This study adopted questionnaire survey (***Questionnaire on medical staff’s Knowledge, Attitudes and Behavior of dysphagia assessment***), knowledge, attitude and behavior are the dimensions of this questionnaire, and the score of the questionnaire was used as the independent variable in the univariate analysis and multiple regression analysis in this study. These three dimensions are different. Due to the length of the paper, We didn’t explained separately, and there are plans to analyze and explain them separately in the future.

*23. “As seen in Table 3, medical staff in Shaanxi Province were more willing to assess dysphagia and were evaluated more frequently.”*

*I do not understand this sentence.*

*In addition, sentences in the “Note” part are difficult to understand.*

**The author’s answer:** We have revised the note part in Table 3[Page 7]. One-way ANOVA showed differences in attitudes, behaviors and total questionnaire scores of medical staff in different provinces. Since there were four provinces, post hoc multiple comparisons were made between groups, and the results were written in the “note” part and marked with symbols.

*24. Table 5 should be removed or moved to the Supplementary Files. The authors can mention reference groups in Table 6.*

*For example, related training for dysphagia (ref: No): Yes.*

**The author’s answer:** We have moved Table 5 to the supplementary files and revised Table 6 according to your suggestion. Please see details in the article[Page 8-9].

Discussion

*25. “This suggests that people are willing to assess swallowing disorders, but lack Knowledge and Behavior.”*

*This sentence is unclear.*

**The author’s answer:** We have revised this sentence as follows[Page 9]:

This showed that the attitude score of medical staff is higher than the behavior score because the standardized score of attitude is 89.7, which is much higher than the standardized score of behavior 63.7.

*26. Please add a paragraph involving the strengths and limitations of this study.*

**The author’s answer:** We have added a paragraph involving the strengths and limitations of this study as follows[Page 13]:

The strengths and limitations of this study

First, participants in this study were from four provinces in China, showing the differences in knowledge, attitude and behavior of medical staff in different regions in dysphagia assessment. Second, this study explored the influencing factors of medical staff's knowledge, attitude and behavior in tdysphagia assessment, and provided guidance and suggestions for the future organization of relevant training. But, this study only investigated hospital and community nurses, and did not investigate healthcare professionals in other different Settings such as nursing home settings. Future studies should focus on the evaluation of dysphagia and provide training for the assessment of swallowing disorders to medical staff in nursing home settings, social health service centers, and palliative care facilities.

*27. Please check the references. I do not know what [J] means.*

**The author’s answer:** We have checked the references according to the PLOS ONE guidelines for References. Please see “References” for details in the article[Page 14-17].

*28. Please check spelling and grammar mistakes.*

**The author’s answer:** We have checked spelling and grammar mistakes, Please see the details in the article.

Thank you for your constructive suggestions.

**Reviewer #2**:

| **Research Paper Title:** Predictors of medical staff’s Knowledge, Attitudes, and Behavior of dysphagia  assessment: A Cross-Sectional study  **Manuscript ID:** PONE-D-23-41544 | | |
| --- | --- | --- |
| **Reviewer 2 comments**  This is a very interesting study and was an enjoyable read. Please see specific comments that will add strength and clarity to the study information presented regarding this topic of predictors of medical staff’s Knowledge, Attitudes, and Behavior of dysphagia assessment. The manuscript needs proofreading from an agency specialized in this task. | | |
| **Page Number and Line** | **Item to Address** | **Author Response** |
| Page 1, Author Affiliation | The affiliation between the majority of authors is the Department of Nursing, & the Department of Neurology. It is better to revise & arrange numbers accordingly. | Thank you for your constructive suggestions. We have revised and arranged numbers accordingly [Page 1]. |
| Page 1, Running head | The running head should be briefed:  Predictors of medical staff’s KAP of dysphagia assessment | Thank you for your constructive suggestions. The running head revised as Predictors of medical staff’s KAP of dysphagia assessment [Page 1] |
| Page 1, Abstract | See the author's instructions for PLOS ONE and use the same writing style. In methods & results, an italic style is used together with other styles. | Thank you for your constructive suggestions. We have revised the abstract according to the author’s instructions for PLOS ONE and use the same writing style. Please see the details in the article [Page 1-2]. |
| Page 1, Abstract: Methods | Try to avoid abbreviations in the abstract, so write the complete Tencent QQ in the abstract. | We have removed abbreviations in the abstract, “Tencent QQ” is replaced by “Tencent instant messenger” [Page 1] |
| Page 1, Abstract: Results | - In the results put the average & SD within brackets and connect them with (Mean ±SD). - To be more organized & not confusing to the reader, I think it’s better to write ONE digit only after the decimal point and this would be applied to all results within context & tables. - Avoid using “&” in writing the manuscript, use and instead of it. - In the last line of the results of the abstract be careful for s after the apostrove and don’t keep space. | Thank you for your constructive suggestions. We have revised according to your suggestions, Please see the details in the article [Page 1-2]. |
| Page 1, Abstract: Conclusions | Try to avoid first, second, and third singular or plural in objective or possessive cases while writing the manuscript. Revise the whole manuscript & change accordingly. In this case, you can write (The study findings instead of Our findings). | Thank you for your constructive suggestions. We have revised the whole manuscript & change according to your suggestions, Please see the details in the article. |
| Page 2, Keywords | It is better to write keywords in alphabetical. | Key words: assessment, Attitude, Behavior, Cross-Sectional, dysphagia, Knowledge, Predictors, stroke |
| Page 2, Introduction  Page 2, last 4 lines of Introduction | - Write a decimal with one digit for each percentage to have consistency for all numbers & this would be applied to all results within the whole context & tables of this study. - Consistency should be also for writing the whole number by adding 0 digits after decimal 90.0%. This could be applied to all numbers within the study. - Further elaboration should be mentioned before aiming that focus on the rationale for conducting this study in China, and previous studies that dealt with this issue in worldwide countries if no study in China deals with knowledge, attitude, and behavior of dysphagia assessment. | We have revised each percentage with one digit within the whole context and table of this study.  We have mentioned further elaboration as follows [Page 3]:  Studies on the factors contributing to barriers to the assessment of dysphagia are lacking. Some Chinese researchers investigated the practice and management of medical staff's dysphagia assessment, it showed that More than 96.0% of the nurses did not know anything about the swallowing function training, and only 36.7% of the nurses used the scale to screen the patients for swallowing disorders[14]. And these studies did not explore the influencing factors. We aimed to explore......  [14] Zhang Y, Liu Y, Li W, Lin Y. Investigation on the knowledge and intervention status of swallowing function training in elderly patients by nurses in Guangzhou and Shenzhen. Journal of Nurses Training, 2018,33(16):1499-1500,1531.DOI:10.16821/j.cnki.hsjx.2018.16.021. |
| Page 2-4, Methods | There are a lot of issues that have to be addressed in the methods section as the following:   - It is unclear if the researcher added a few questions to the original instrument & checked for external validity. How questionnaire was administered in English or Chinese language? - What is about reliability & validity of the different tools used in this study? - The researcher mentioned knowledge, and attitudes (8 items), while at the end of page 3, the knowledge domain is composed of 25 items that will be confusing for the reader. - Scoring is not clear & confused also. Explain it clearly. - It is better to write the parts of the research instrument as “The following instrument covered two parts a) The general information sheet…….., and b) Assessment of knowledge, attitude, and behavior of dysphagia. - Why convenience sampling approach was used in this study? To generalize findings it is better to utilize probability approach rather than non-probability. For this study the proper sampling is cluster. - What does it mean by “a questionnaire that completed less than 300 s and more than 3600 s”? It is not clear. - As mentioned before avoid using the pronoun as the third person in the manuscript & revise it again to change accordingly. - There is no need for repetition of ethical approval when data is collected, & naming the four provinces. The first two sentences and the last line in the data collection and analysis subheading are to be deleted. Start the paragraph from “All participants……..among FOUR provinces. - Instead of “n” in the example of descriptive statistics, it is better to write frequency. Descriptive statistics (e.g., Mean ±SD, frequency & %). | We have addressed in the methods section as the following [Page 4]:  Before the study, we obtained the consent of the author of the original questionnaire to add the scoring method and paraphrase the items of knowledge dimension on the basis of the original questionnaire......Internal consistency estimates were shown to have acceptable reliability for the questionnaire before the survey (Cronbach’s alpha for domain K, A, and B are 0.77, 0.97 and 0.91, respectively). The instrument was administered in Chinese language and designed as a google form, the website was https://www.wjx.cn/vm/mc5kOo1.aspx.  The researcher sent the questionnaire website through WeChat, DingTalk, and Tencent instant messenger to colleagues and classmates to fill in and asked them to forward the questionnaire to their colleagues.    We have revised Questionnaire introduction (domain, items and scoring), please see the details in the article. And we have added a Supporting information as follows [Page 4, 13-14]:    We have rewritten the parts of the research instrument according your suggestions [Page 3-4].  It is difficult to utilize probability approach because of large sample size and area. So we used convenience sampling.  “a questionnaire that completed less than 300 s and more than 3600 s” means the time to fill in the questionnaire is shorter than 300 seconds and longer than 3600 seconds. We have revised the sentence [Page 3].  According to the test, it took 300 to 1800 seconds (5-30 minutes) to fill out a questionnaire. In consideration of the possibility of interruptions during the filling process, an additional 30 minutes was added. If it takes less than 5 minutes to fill in the questionnaire, it is likely that the person who filled in the questionnaire did not read the question carefully and filled in randomly  We have checked the paper throughout for avoiding using the pronoun as the third person in the manuscript.  We have removed the description of ethical approval and naming the four provinces in the data section. And the first two sentences and the last line in the data collection and analysis subheading had been deleted [Page 4].  We have revised as follows [Page 4]:  Descriptive statistics ( e.g., Mean ±SD, frequency & %) were...... |
| Page 4-9: Results | There are a lot of issues that have to be addressed in this section:   - Numbers within tables have to be changed into one digit after the decimal point like this 13(6.2) in the first row and be careful to have 100 % to the total of each column. - For table 1 the total number is 328 (211+77+40=328). A total of 25 subjects are missed. What about the demographic characteristics of the missed province (Guangxi Province)? Rewrite the table again & revise the number & percentage for each province. Calculation of F, t. Mean has to consider the missed province. Check again your SPSS to ensure the missed one is included in all calculations. - Correct the spelling mistakes of a few words within tables & whole manuscript. As I suggested this manuscript needs proofreading from one agency expert in this field. - Use (Mean ±SD) for tables 2 & 3 to distinguish them from other tables of N (%). - For each variable in the first column of all tables write the first letter as capital (Hospital level- Level 1 hospital etc…). Be consistent in writing & use the same style. Don’t use two styles within the same sentence & be careful with font size also. As seen in Table 3, medical staff in Shaanxi Province This would be applied for all tables from 1-6. - Subheadings should not be in the same font and style. This should be applied to all sections. | We have revised the Tables according your suggestions, Please see the details in the article.  We have checked my SPSS and rewritten the Table 1 and revised the number & percentage for each province (including Guangxi Province). Please see the Table 1 for details in the article [Page 5-6].  We have checked the manuscripts and forms for spelling errors.  We have revised (Mean ±SD) for tables 2 & 3 [Page 6-7].  We have revised table1-6 according to PLOS ONE Table Guidelines. Please see the details in the article.  We have revised heading according to PLOS ONE Heading Guidelines. Please see the details in the article. |
| Page 9-12: Discussion | - The author can not say in our study (In this study). - Notes mentioned before regarding percentages & consistency in writing styles should applied in this section. - In discussion, the author can say findings are consistent or contradict previous studies. Try to utilize these in the discussion section to show similarities & differences within the study conducted & give a rationale for your results. - I grasp from the discussion, that few studies were conducted in China that are similar to this study. The authors have to explain the reasons for conducting this study, particularly in four provinces. Further elaboration had to be discussed in the introduction section as mentioned before. - The discussion section needs to be organized well & deal with the recent studies to compare and interpret study findings. | We have replaced “in our study” with “in this study”.  We have checked discussion section for percentages & consistency in writing styles.  We have added a description of the comparison with other studies and explained the reasons for similarities & differences in the first paragraph of the discussion.As follows [Page 9]:  The results of this study are different from study of Sun Qian et al^[19]^. The percentige rating score of knowledge and attitude in this study is similar to study of Sun Qian, which is at the medium and high level respectively. However, the percentige rating score of behavior in this study is slightly lower than study of Sun Qian (63.7 VS 71.0). The reasons may be as follows: Firstly, different participants and locations were surveyed in two studies. Sun Qian surveyed nurses in 17 Grade III hospitals in Beijing, while this study surveyed medical staff in hospitals of different levels in four provinces. Secondly, different departments were surveyed in two studies. Sun Qian only investigated the department of neurosurgery, which is known to pay more attention to the evaluation practice of swallowing disorders than other departments.  [19] Sun Q, Su Y, Wang Y.Investigation on the current situation of knowledge and practice in nursing of dysphagia after stroke in neurology nurses. Chinese journal of modern nursing,2020,26(23):3144-3150. DOI:10.3760/cma.j.cn115682-20200322-02063.  We have added a description of explaining the reasons for conducting this study as follows [Page 9]:  The knowledge of medical staff on dysphagia assessment directly affects the quality of care and the prognosis of patients. However, most nurses lack the knowledge of dysphagia assessment, which may be related to lack of relevant training and attention paid by medical institutions to the knowledge education of nurses^[18]^. To build an appropriate and effective training program, it is necessary to combine the influencing factors of nurses' knowledge, attitude and behavior in the assessment of swallowing disorders. There is no research on the influencing factors of knowledge, attitude and behavior of dysphagia assessment in China. This study was conducted in multiple regions (4 provinces), multiple populations (doctors, clinical and community nurses, technicians, etc.) and multiple departments (neurosurgery and general departments) aiming to find as different data and features as possible to serving the training program.  [18] Lv S, Zhou G, Mao Y, Zhang Y, Wang J. Research progress on the attitude, knowledge and behavior of nurses and caregivers for swallowing disorders after stroke in China. Chinese General practice nursing,2023,21(1):37-41. DOI:10.12104/j.issn.1674-4748.2023.01.009.  Discussion section have been organized. |
| Page 12-13: Conclusion | - Be specific in your conclusion of this study. Nothing is mentioned about the conclusion in that section. Revise this section & add a conclusion. | We have added specific conclusion as follows [Page 13]:  Conclusion  The attitude of Chinese medical staff to the dysphagia assessment is positive, but the knowledge and behavior needs to be improved, and the attitude is separated from the knowledge and the behavior.This study findings also imply that nursing experience, training, and work for patients with swallowing disorders could have positive effects on the Knowledge, Attitudes and Behavior of medical staff regarding dysphagia assessment. The medical management department should take various forms to train the knowledge and skills related to the evaluation of swallowing disorders. Hospital administrators should provide relevant resources, such as videos of dysphagia assessment, training centers for the assessment of dysphagia, and swallowing specialist nurses. It is important that health policies fully recognize the role of training and support systems in caring for people with dysphagia. |
| Page 13-16: References | - The first reference- Johnson, C. O., Nguyen, M., Roth, G. A., et al. (2019). Global, regional, and national burden of stroke, 1990– 2016: a systematic analysis for the Global Burden of Disease Study 2016. Lancet Neurology, 18(5), 439–458. https://doi.org/10.1016/S1474-4422(19)30034-1 is different from the second one. - Check again reference # 32 is different from the previous one - Authors exceeding three or more authors should be written with et al at the end, but this is not applied for all references as in the first reference. Compare the first reference with the second & third one as an example to see the difference. - Journal abbreviation should be as in index medicus & pay attention to be written with dots in between or without (Ann Med Res) and consistency in written abbreviation. - Ten out of thirty-six references were old. Why? I hope to add more recent citations in the introduction & discussion sections instead of these old references. - I request to revise all references and pay attention to PLOS ONE author guidelines. - There are a lot of spelling errors in this section. Revise & correct them. | Thank you for your constructive suggestions. We have revised references according your suggestions and PLOS ONE guidelines for References, and checked spelling errors in References section. Please see the details in the article.  We have re-searched the recent relevant literature and replaced some old literature. Some older literature were not replaced because there were important data or authoritative theories citations [Page 14-17]. |
