# Supplementary material for: Predictors of medical staff’s knowledge, attitudes and behavior of dysphagia assessment: A cross-sectional study
Source: PLoS One. 2024 Apr 5;19(4):e0301770. doi: 10.1371/journal.pone.0301770 (PMC10997058; doi:10.1371/journal.pone.0301770)
Supplement: Attachment — Submitted filename: Response to Reviewers.doc [file pone.0301770.s012.doc]

Response to reviewers

Dear editors and reviewers:

Thank you for your letter and for the reviewer’s comments concerning our manuscript entitled “Predictors of medical staff’s Knowledge, Attitudes and Behavior of dysphagia assessment: A Cross-Sectional study”(ID:PONE-D-23-41544). Those comments are all valuable and very helpful for revising and improving our paper, as well as the important guiding significance to our researches. We have studied comments carefully and have made correction which we hope meet with approval.

We submited four items as follow:

1. Response to Reviewers
2. Revised manuscript with track changes
3. Manuscript (revised)

Below, please find a response to each of the reviewer’s comments. We have revised manuscript with track changes. We hope that the revisions in the manuscript and our accompanying responses will meet editors’ and reviewers’ criteria.

We shall look forward to hearing from you at your earliest convenience.

Yours sincerely

Juanhui Chen

**Reviewer #1**:

*1. In the Abstract, the authors should mention whether the knowledge, attitude, and behavior of medical staff were good or poor, positive or negative.*

**The author’s answer:** Thank you for your constructive suggestions. We have revised the sentences in the abstract as follows[Page 1]:

Results: The mean scores for Knowledge, Attitudes and Behavior of dysphagia assessments was (15.3±2.7), (35.9±4.9) and (41.4±14.4) respectively. Knowledge and Behavior of medical staff were medium, and attitude was positive.

*2. In the Data analysis, the authors mentioned, “A comparison analysis was conducted to analyze the differences in medical staff’s KAB for dysphagia assessment among four Provinces.”*

*Tests used should be described in detail.*

**The author’s answer:** Thank you for your constructive suggestions. We have revised the sentence in the data analysis as follows[Page 4]:

One-way analysis of variance and post hoc multiple comparisons were conducted to analyze the differences in medical staff’s K, A and B for dysphagia assessment among four Provinces.

*3. For my comment, “Can the authors add some sentences to introduce community nurses?”, I do not recommend the authors count the number of community nurses in each hospital.*

*I mean their definitions (at the bottom of Table 1). Because in my country, we do not have “community” nurses (I rarely hear about them). I think we should have definitions for occupations that are not internationally popular (if necessary - authors' choice).*

**The author’s answer:** Thank you for your constructive suggestions. We have deleted the sentences of the number of community nurses in each hospital and added the definition of community nurses at the bottom of Table 1. As follows[Page 6]:

Note. *Community nurses refer to nursing professionals who are engaged in community nursing work in community health institutions and other relevant medical institutions. Community nursing focuses on families, communities and related groups.

*4. I still do not think adding the knowledge, attitude, and behavior scores into a total score is a good idea. These three domains are different.*

*For example, working experience is a predictor of medical staff’s KAB. It means that working experience is associated with all their knowledge, attitudes, and behavior. Is it true? However, in this study, “their knowledge and behavior scores were medium, but the attitude scores were high”. In addition, the authors did not demonstrate the validity of the questionnaire (EFA and CFA) to analyze the relationship among three domains (K, A, and B). This means combining these three domains into one is meaningless.*

**The author’s answer:** Thank you for your constructive suggestions. We have revised in the article, the knowledge, attitude and behavior scores were analyzed and described as individual scores, without counting into a total score. Please see the detail in the article.

**Reviewer #2**:

**The author’s answer:**Thank you for your constructive suggestions. We have revised according to your suggestions, Please see the detail in the article.

1. The sentence “It is important that health policies fully recognize the role of training and support systems in caring for people with dysphagia” in the Conclusions had been deleted.
2. We have re-searched the relevant literature and checked the data of the sentence in the introduction “*Dysphagia occurs in 30–78% of patients after stroke [2-4]*”.
[2] Vilardell N, Rofes L, Nascimento WV, Muriana D, Palomeras E, Clavé P. Cough reflex attenuation and swallowing dysfunction in sub-acute post-stroke patients: prevalence, risk factors, and clinical outcome. Neurogastroenterol Motil. 2017 Jan;29(1). doi: 10.1111/nmo.12910. Epub 2016 Jul 18. PMID: 27424849.

It is shown in reference 2 that “We included 225 PSP. Prevalence of impaired CRT was 5.8%, that of OD was 40.4% (20.4% with impaired safety of swallow), and of both impairments was, 1.8%”.

[3] Matsumura T, Mitani Y, Oki Y, Fujimoto Y, Ishikawa A. Risk factors for the onset of aspiration pneumonia among stroke patients in the recovery stage. Nihon Ronen Igakkai Zasshi. 2014;51(4):364-8.doi: 10.3143/geriatrics.51.364. PMID: 25327371.

It is shown in reference 3 that “The study subjects included 76 stroke patients (mean age, 74.7±8.4 years) with dysphagia chosen from 175 stroke patients who were newly admitted to four rehabilitation hospitals”. According to the above data, the incidence of dysphagia in stroke patients was 43.43%.

[4] Finnegan BS, Meighan M M, Warren NC, Hatfield MK, Alexeeff S, Lipiz J, et al. Validation Study of Kaiser Permanente Bedside Dysphagia Screening Tool in Acute Stroke Patients. Perm J. 2020 Dec;24:1. doi: 10.7812/TPP/19.230. PMID: 33482958; PMCID: PMC7849282.

It is shown in reference 4 that “Difficulty swallowing or dysphagia is common among acute

stroke patients, occurring in approximately 30% to 78% of the population”.
