# Supplementary material for: Predictors of medical staff’s knowledge, attitudes and behavior of dysphagia assessment: A cross-sectional study
Source: PLoS One. 2024 Apr 5;19(4):e0301770. doi: 10.1371/journal.pone.0301770 (PMC10997058; doi:10.1371/journal.pone.0301770)
Supplement: Attachment — Submitted filename: Reviewer comments- PLOS ONE.docx [file pone.0301770.s008.docx]

| **Research Paper Title:** Predictors of medical staff’s Knowledge, Attitudes, and Behavior of dysphagia  assessment: A Cross-Sectional study  **Manuscript ID:** PONE-D-23-41544 | | |
| --- | --- | --- |
| **Reviewer 1 comments**  This is a very interesting study and was an enjoyable read. Please see specific comments that will add strength and clarity to the study information presented regarding this topic of predictors of medical staff’s Knowledge, Attitudes, and Behavior of dysphagia assessment. The manuscript needs proofreading from an agency specialized in this task. | | |
| **Page Number and Line** | **Item to Address** | **Author Response** |
| Page 1, Author Affiliation | The affiliation between the majority of authors is the Department of Nursing, & the Department of Neurology. It is better to revise & arrange numbers accordingly. |  |
| Page 1, Running head | The running head should be briefed:  Predictors of medical staff’s KAP of dysphagia assessment |  |
| Page 1, Abstract | See the author's instructions for PLOS ONE and use the same writing style. In methods & results, an italic style is used together with other styles. |  |
| Page 1, Abstract: Methods | Try to avoid abbreviations in the abstract, so write the complete Tencent QQ in the abstract. |  |
| Page 1, Abstract: Results | - In the results put the average & SD within brackets and connect them with (Mean ±SD). - To be more organized & not confusing to the reader, I think it’s better to write ONE digit only after the decimal point and this would be applied to all results within context & tables. - Avoid using “&” in writing the manuscript, use and instead of it. - In the last line of the results of the abstract be careful for s after the apostrove and don’t keep space. |  |
| Page 1, Abstract: Conclusions | Try to avoid first, second, and third singular or plural in objective or possessive cases while writing the manuscript. Revise the whole manuscript & change accordingly. In this case, you can write (The study findings instead of Our findings). |  |
| Page 2, Keywords | It is better to write keywords in alphabetical. |  |
| Page 2, Introduction  Page 2, last 4 lines of Introduction | - Write a decimal with one digit for each percentage to have consistency for all numbers & this would be applied to all results within the whole context & tables of this study. - Consistency should be also for writing the whole number by adding 0 digits after decimal 90.0%. This could be applied to all numbers within the study. - Further elaboration should be mentioned before aiming that focus on the rationale for conducting this study in China, and previous studies that dealt with this issue in worldwide countries if no study in China deals with knowledge, attitude, and behavior of dysphagia assessment. |  |
| Page 2-4, Methods | There are a lot of issues that have to be addressed in the methods section as the following:   - It is unclear if the researcher added a few questions to the original instrument & checked for external validity. How questionnaire was administered in English or Chinese language? - What is about reliability & validity of the different tools used in this study? - The researcher mentioned knowledge, and attitudes (8 items), while at the end of page 3, the knowledge domain is composed of 25 items that will be confusing for the reader. - Scoring is not clear & confused also. Explain it clearly. - It is better to write the parts of the research instrument as “The following instrument covered two parts a) The general information sheet…….., and b) Assessment of knowledge, attitude, and behavior of dysphagia. - Why convenience sampling approach was used in this study? To generalize findings it is better to utilize probability approach rather than non-probability. For this study the proper sampling is cluster. - What does it mean by “a questionnaire that completed less than 300 s and more than 3600 s”? It is not clear. - As mentioned before avoid using the pronoun as the third person in the manuscript & revise it again to change accordingly. - There is no need for repetition of ethical approval when data is collected, & naming the four provinces. The first two sentences and the last line in the data collection and analysis subheading are to be deleted. Start the paragraph from “All participants……..among FOUR provinces. - Instead of “n” in the example of descriptive statistics, it is better to write frequency. Descriptive statistics (e.g., Mean ±SD, frequency & %). |  |
| Page 4-9: Results | There are a lot of issues that have to be addressed in this section:   - Numbers within tables have to be changed into one digit after the decimal point like this 13(6.2) in the first row and be careful to have 100 % to the total of each column. - For table 1 the total number is 328 (211+77+40=328). A total of 25 subjects are missed. What about the demographic characteristics of the missed province (Guangxi Province)? Rewrite the table again & revise the number & percentage for each province. Calculation of F, t. Mean has to consider the missed province. Check again your SPSS to ensure the missed one is included in all calculations. - Correct the spelling mistakes of a few words within tables & whole manuscript. As I suggested this manuscript needs proofreading from one agency expert in this field. - Use (Mean ±SD) for tables 2 & 3 to distinguish them from other tables of N (%). - For each variable in the first column of all tables write the first letter as capital (Hospital level- Level 1 hospital etc…). Be consistent in writing & use the same style. Don’t use two styles within the same sentence & be careful with font size also. As seen in Table 3, medical staff in Shaanxi Province This would be applied for all tables from 1-6. - Subheadings should not be in the same font and style. This should be applied to all sections. |  |
| Page 9-12: Discussion | - The author can not say in our study (In this study). - Notes mentioned before regarding percentages & consistency in writing styles should applied in this section. - In discussion, the author can say findings are consistent or contradict previous studies. Try to utilize these in the discussion section to show similarities & differences within the study conducted & give a rationale for your results. - I grasp from the discussion, that few studies were conducted in China that are similar to this study. The authors have to explain the reasons for conducting this study, particularly in four provinces. Further elaboration had to be discussed in the introduction section as mentioned before. - The discussion section needs to be organized well & deal with the recent studies to compare and interpret study findings. |  |
| Page 12-13: Conclusion | - Be specific in your conclusion of this study. Nothing is mentioned about the conclusion in that section. Revise this section & add a conclusion. |  |
| Page 13-16: References | - The first reference- Johnson, C. O., Nguyen, M., Roth, G. A., et al. (2019). Global, regional, and national burden of stroke, 1990– 2016: a systematic analysis for the Global Burden of Disease Study 2016. Lancet Neurology, 18(5), 439–458. https://doi.org/10.1016/S1474-4422(19)30034-1 is different from the second one. - Check again reference # 32 is different from the previous one - Authors exceeding three or more authors should be written with et al at the end, but this is not applied for all references as in the first reference. Compare the first reference with the second & third one as an example to see the difference. - Journal abbreviation should be as in index medicus & pay attention to be written with dots in between or without (Ann Med Res) and consistency in written abbreviation. - Ten out of thirty-six references were old. Why? I hope to add more recent citations in the introduction & discussion sections instead of these old references. - I request to revise all references and pay attention to PLOS ONE author guidelines. - There are a lot of spelling errors in this section. Revise & correct them. |  |
