# Supplementary material for: Predictors of medical staff’s knowledge, attitudes and behavior of dysphagia assessment: A cross-sectional study
Source: PLoS One. 2024 Apr 5;19(4):e0301770. doi: 10.1371/journal.pone.0301770 (PMC10997058; doi:10.1371/journal.pone.0301770)
Supplement: Attachment — Submitted filename: PONE-D-23-41544 Reviewer Comments.pdf [file pone.0301770.s009.pdf]

PLOS ONE

Predictors of medical staff's Knowledge, Attitudes and Behavior of dysphagia assessment: A Cross-Sectional study

--Manuscript Draft--

**Manuscript Number:**	PONE-D-23-41544
**Article Type:**	Research Article
**Full Title:**	Predictors of medical staff's Knowledge, Attitudes and Behavior of dysphagia assessment: A Cross-Sectional study
**Short Title:**	Predictors of medical staff's Knowledge, Attitudes and Behavior of dysphagia assessment
**Corresponding Author:**	Juanhui Chen<br>Longgang Central Hospital of Shenzhen<br>Shenzhen, Guangdong province CHINA
**Keywords:**	dysphagia, assessment, stroke, Knowledge, Attitude, Behavior, Cross-Sectional, Predictors
**Abstract:**	Purpose: This study aimed to develop training resources and standardize the assessment of dysphagia in patients with stroke.<br>Methods: This study was a cross-sectional study. A total of 430 nurses and doctors from four provinces(Guangdong Province, Hunan Province, Guangxi Province, and Shaanxi Province) who were selected by convenience sampling were invited to complete the questionnaire through WeChat, DingTalk, and Tencent QQ from May 23 to 31, 2022. A self-reported questionnaire was used to assess participants' Knowledge, Attitude and Behavior regarding dysphagia. Participants' sociodemographic, training, and nursing experience were measured using the general information sheet and assessed as potential predictors of medical staff's Knowledge, Attitudes and Behavior of dysphagia assessment. A multiple linear regression model was used to identify the factors predicting medical staff's Knowledge, Attitudes and Behavior regarding dysphagia assessment.<br>Results: The mean scores for Knowledge, Attitudes and Behavior of dysphagia assessments were 92.654(SD 17.519). Multiple linear regression results indicated that experience of dysphagia patients' nursing, related training for dysphagia, working years in the field of dysphagia related diseases, specialized training in geriatric, swallowing & rehabilitation and department related to neurology, rehabilitation & elderly were significant predictors, accounting for 35.1% of the variance in scores of medical staff's Knowledge, Attitudes and Behavior of dysphagia assessment.<br>Conclusions: Our findings imply that nursing experience, training, and work for patients with swallowing disorders could have positive effects on the Knowledge, Attitudes and Behavior of medical staff regarding dysphagia assessment. Hospital administrators should provide relevant resources, such as videos of dysphagia assessment, training centers for the assessment of dysphagia, and swallowing specialist nurses. It is important that health policies fully recognize the role of training and support systems in caring for people with dysphagia.
**Order of Authors:**	Juanhui Chen
	Wenqiu Ye
	Xingyun Zheng
	Wenna Wu
	Yuebao Chen
	Yinjuan Chen
**Additional Information:**	
**Question**	**Response**
**Financial Disclosure**	The author(s) received no specific funding for this work.

Powered by Editorial Manager® and ProduXion Manager® from Aries Systems Corporation

Enter a financial disclosure statement that describes the sources of funding for the work included in this submission. Review the submission guidelines for detailed requirements. View published research articles from *PLOS ONE* for specific examples.<br><br>This statement is required for submission and **will appear in the published article** if the submission is accepted. Please make sure it is accurate.<br><br>**Funded studies**<br>Enter a statement with the following details:<br>• Initials of the authors who received each award<br>• Grant numbers awarded to each author<br>• The full name of each funder<br>• URL of each funder website<br>• Did the sponsors or funders play any role in the study design, data collection and analysis, decision to publish, or preparation of the manuscript?<br><br>Did you receive funding for this work?	
**Competing Interests**<br><br>Use the instructions below to enter a competing interest statement for this submission. On behalf of all authors, disclose any competing interests that could be perceived to bias this work—acknowledging all financial support and any other relevant financial or non-financial competing interests.<br><br>This statement is required for submission and **will appear in the published article** if the submission is accepted. Please make sure it is accurate and that any funding sources listed in your Funding Information later in the submission form are also declared in your Financial Disclosure statement.<br><br>View published research articles from	The authors have declared that no competing interests exist.

PLOS ONE for specific examples.<br><br>**NO authors have competing interests**<br><br>Enter: *The authors have declared that no competing interests exist.*<br><br>**Authors with competing interests**<br><br>Enter competing interest details beginning with this statement:<br><br>*I have read the journal's policy and the authors of this manuscript have the following competing interests: [insert competing interests here]*<br><br>* typeset	
**Ethics Statement**<br><br>Enter an ethics statement for this submission. This statement is required if the study involved:<br><br>• Human participants<br>• Human specimens or tissue<br>• Vertebrate animals or cephalopods<br>• Vertebrate embryos or tissues<br>• Field research<br><br>Write "N/A" if the submission does not require an ethics statement.<br><br>General guidance is provided below. Consult the submission guidelines for detailed instructions. **Make sure that all information entered here is included in the Methods section of the manuscript.**	This study was approved by the Ethics Committee of Longgang Central Hospital of Shenzhen(No. 2022ECPJ101)

**Format for specific study types**<br><br>**Human Subject Research (involving human participants and/or tissue)**<br>• Give the name of the institutional review board or ethics committee that approved the study<br>• Include the approval number and/or a statement indicating approval of this research<br>• Indicate the form of consent obtained (written/oral) or the reason that consent was not obtained (e.g. the data were analyzed anonymously)<br><br>**Animal Research (involving vertebrate animals, embryos or tissues)**<br>• Provide the name of the Institutional Animal Care and Use Committee (IACUC) or other relevant ethics board that reviewed the study protocol, and indicate whether they approved this research or granted a formal waiver of ethical approval<br>• Include an approval number if one was obtained<br>• If the study involved *non-human primates*, add *additional details* about animal welfare and steps taken to ameliorate suffering<br>• If anesthesia, euthanasia, or any kind of animal sacrifice is part of the study, include briefly which substances and/or methods were applied<br><br>**Field Research**<br><br>Include the following details if this study involves the collection of plant, animal, or other materials from a natural setting:<br>• Field permit number<br>• Name of the institution or relevant body that granted permission	
**Data Availability**<br><br>Authors are required to make all data underlying the findings described fully available, without restriction, and from the time of publication. PLOS allows rare exceptions to address legal and ethical concerns. See the PLOS Data Policy and FAQ for detailed information.	Yes - all data are fully available without restriction

A Data Availability Statement describing where the data can be found is required at submission. Your answers to this question constitute the Data Availability Statement and **will be published in the article**, if accepted.<br><br>**Important:** Stating 'data available on request from the author' is not sufficient. If your data are only available upon request, select 'No' for the first question and explain your exceptional situation in the text box.<br><br>Do the authors confirm that all data underlying the findings described in their manuscript are fully available without restriction?	
**Describe where the data may be found in full sentences. If you are copying our sample text, replace any instances of XXX with the appropriate details.**<br><br>• If the data are **held or will be held in a public repository**, include URLs, accession numbers or DOIs. If this information will only be available after acceptance, indicate this by ticking the box below. For example: *All XXX files are available from the XXX database (accession number(s) XXX, XXX.).*<br>• If the data are all contained **within the manuscript and/or Supporting Information files**, enter the following: *All relevant data are within the manuscript and its Supporting Information files.*<br>• If neither of these applies but you are able to provide **details of access elsewhere**, with or without limitations, please do so. For example:<br><br>*Data cannot be shared publicly because of [XXX]. Data are available from the XXX Institutional Data Access / Ethics Committee (contact via XXX) for researchers who meet the criteria for access to confidential data.*<br><br>*The data underlying the results presented in the study are available from (include the name of the third party*	All relevant data are within the manuscript and its Supporting Information files.

and contact information or URL).<br>• This text is appropriate if the data are owned by a third party and authors do not have permission to share the data.<br><br>* typeset	
Additional data availability information:	Tick here if the URLs/accession numbers/DOIs will be available only after acceptance of the manuscript for publication so that we can ensure their inclusion before publication.

Manuscript Click here to access/download;Manuscript;2.main manuscript2023-12-26.doc

Predictors of medical staff's Knowledge, Attitudes and Behavior of dysphagia assessment: A Cross-Sectional study

Juanhui Chen[1*], Wenqiu Ye[2], Xingyun Zheng[3], Wenna Wu[4], Yuebao Chen[5], Yinjuan Chen[6]

1 Department of Nursing, 2 Department of Neurology, 3 Department of Neurosurgery, and 4 Department of Rehabilitation, Longgang Central Hospital of Shenzhen, Shenzhen, Guangdong province, China.

5 Department of Neurology, Guangxi International Zhuang Medicine hospital, Nanning, Guangxi province, China

6 Department of Nursing, Shenzhen FuYong People's Hospital, Shenzhen, Guangdong province, China

Running head: Predictors of medical staff' s Knowledge, Attitudes and Behavior of dysphagia assessment

***Correspondence**

E-mail:pugongyingchenjuan@163.com(JHC)

Project: Unfunded project of Medical and Health Technology Plan of Longgang District, Shenzhen(No. LGKCYLWS2020128)

Abstract

Purpose: This study aimed to develop training resources and standardize the assessment of dysphagia in patients with stroke.

Methods: This study was a cross-sectional study. A total of 430 nurses and doctors from four provinces(Guangdong Province, Hunan Province, Guangxi Province, and Shaanxi Province) who were selected by convenience sampling were invited to complete the questionnaire through WeChat, DingTalk, and Tencent QQ from May 23 to 31, 2022. A self-reported questionnaire was used to assess participants' Knowledge, Attitude and Behavior regarding dysphagia. Participants' sociodemographic, training, and nursing experience were measured using *the general information sheet* and assessed as potential predictors of medical staff' s Knowledge, Attitudes and Behavior of dysphagia assessment. A multiple linear regression model was used to identify the factors predicting medical staff's Knowledge, Attitudes and Behavior regarding dysphagia assessment.

Results: The mean scores for Knowledge, Attitudes and Behavior of dysphagia assessments were 92.654(SD 17.519). Multiple linear regression results indicated that *experience of dysphagia patients' nursing, related training for dysphagia, working years in the field of dysphagia related diseases, specialized training in geriatric, swallowing & rehabilitation* and *department related to neurology, rehabilitation & elderly* were significant predictors, accounting for 35.1% of the variance in scores of medical staff' s Knowledge, Attitudes and Behavior of dysphagia assessment.

Conclusions: Our findings imply that nursing experience, training, and work for patients with swallowing disorders could have positive effects on the Knowledge, Attitudes and Behavior of medical staff regarding dysphagia assessment. Hospital administrators should provide relevant resources, such as videos of dysphagia assessment, training centers for the assessment of dysphagia, and swallowing

specialist nurses. It is important that health policies fully recognize the role of training and support systems in caring for people with dysphagia.

Abbreviations: KAB=Knowledge, Attitudes and Behavior, MS-KAB-DA=Questionnaire of Medical staff's Knowledge, Attitudes and Behavior of dysphagia assessment.

Key words: dysphagia, assessment, stroke, Knowledge, Attitude, Behavior, Cross-Sectional, Predictors

Introduction

Worldwide, there are approximately 80.1 million stroke survivors, with an estimated 13.7 million new cases of stroke every year. Stroke is the second largest global cause of disability[1]. Dysphagia occurs in 37–78% of patients after stroke[2-4]. Dysphagia may be defined as the partial or complete inability to prepare and move a bolus of food, fluids, or saliva efficiently and safely from the mouth to the esophagus and stomach[5]. Oropharyngeal Dysphagia(OD) is prevalent in the elderly and people with complex medical conditions; for example, aspiration pneumonia is caused by silent aspiration in stroke patients, resulting in considerable medical psychosocial consequences, reduced quality of life, and affects the prognosis of patients. Prevalence estimates for OD determined by a meta-analysis were 36.5% in hospital settings, 42.5% in rehabilitation settings, and 50.2% in nursing homes[6]. The type and combination of screening and assessment methods used to determine the prevalence of OD varies. More than half of the studies reported OD prevalence data using screening and clinical non-instrumental assessment methods or tools that were either designed by the authors for the purpose of the study or modified versions of published tools, thus lacking information on diagnostic performance and psychometric properties[7-9]. However, instrumental assessments require specialized training and equipment because of feasibility (e.g., availability, ease of administration). Screening and clinical non-instrumental assessments are the natural first choice for estimating the prevalence of OD[10]. Speech-language pathologists(SLPs) in medical settings are responsible for evaluating patient feeding and swallowing. Once an evaluation is completed, nursing staff typically provide hands-on care and supervision of meals[11]. One study's results suggest that both SLP and nursing training programs should include more evidence from multidisciplinary sources, specifically the medical literature, to improve the knowledge base of clinicians providing care to residents with swallowing disorders[12]. One study evaluated nurses' barriers to compliance with dysphagia measures. The study demonstrated that a knowledge deficit was an important barrier in dysphagia care for nurses, and this can be improved with short training[13]. Studies on the factors contributing to barriers to the assessment of dysphagia are lacking. We aimed to explore factors contributing to barriers to the assessment of dysphagia, develop training resources, and improve health outcomes by investigating the predictors of medical staff's Knowledge, Attitudes and Behavior of dysphagia assessment.

Methods

Design and Participants

This was a cross-sectional study. A total of 430 nurses and doctors in four provinces (Guangdong Province, Hunan Province, Guangxi Province, and Shaanxi Province) were selected by convenience sampling. The inclusion criteria were as follows: (a) Chinese RNs, doctors, or therapists with a medical background, (b) working at the hospital for more than 3 months, and (c) not internship.

Based on the suggestion of Hair, Black, Babin, and Anderson[14], a ratio of 10 to 20 participants for each parameter was required for structural equation modeling. Thus, 220–440 participants were followed by a calculation of a 15% attrition rate, which resulted in a total sample of 430 participants in this study.

An anonymous survey was conducted with permission from the research ethics committee of the participating hospital. Participants were informed about the aims and content of the study and the importance of enrollment. A total of 430 nurses and doctors completed the questionnaire, corresponding to a response rate of 100%. The valid questionnaire was 82.09%, and invalid questionnaires were eliminated. The criteria for eliminating invalid questionnaires were (a) incomplete questionnaires, (b) a questionnaire with incorrect content, (c) logically contradictory questionnaires, and (d) a questionnaire that completed less than 300 s and more than 3600 s.

Ethical statements

This study was approved by Ethics Committee of Longgang Central Hospital of Shenzhen(No. 2022ECPJ101). The first part of the questionnaire mainly included informed consent, participants will read the informed consent at first, if they agree to participate in the study they will chose "I agree" to complete the following survey. The questionnaire survey was anonymous and personal information was not disclosed.

Research instruments

The following instrument were used to measure the outcomes of this study.

The general information sheet. Demographic characteristics were measured using the general information sheet, which includes 11 items: province, hospital level and type, department, position, title, working years in the field of dysphagia-related diseases, education, experience of nursing patients with dysphagia, related training for dysphagia, specialized training in geriatric, swallowing, and rehabilitation.

Questionnaire on medical staff's Knowledge, Attitudes and Behavior of dysphagia assessment. Before the study, I obtained the consent of the author of the original questionnaire to modify the questionnaire. This questionnaire was revised based on the existing questionnaires designed by Dr. Dong Xiaofang[15] and Master Ma Keke[16] from The First Affiliated Hospital of Zhengzhou University. The KAB was shortened for Knowledge, Attitudes and Behavior. The MS-KAB-DA was shortened for the Questionnaire of Medical staff's Knowledge, Attitudes, and Behavior of dysphagia assessment, which is composed of three specific domains: Knowledge, Attitude(eight items), and behavior (13 items). The knowledge domain is composed of 25 items,

including 17 True or False items(KR-20=0.760), four single-choice items, and four multiple-choice items. True or false items and single-choice items scored 1 for each correct choice and 0 for the wrong choice. Multiple choice items scored 0.2 for each correct choice and 0 for wrong choice. If a multiple-choice item had three correct choices that could score 0.6 maximally, but score 0 when the wrong choice was selected or the correct and wrong choices were selected simultaneously. Higher scores indicate higher cognitive levels. The total full knowledge score was 24.4. Respondents were asked about their Attitude and Behavior to score using a 5-point scale, ranging from “strongly agreed” to “strongly disagreed” and “the most frequently” to “the least frequently. Higher scores indicated higher willingness and practice levels. The total full scores of Attitude and Behavior were 40 and 65, respectively. The questionnaire demonstrated good reliability and construct validity; the reported Cronbach's α coefficient was 0.832–0.879, and the content validity was 0.751[15-16]. Internal consistency estimates were shown to have acceptable reliability for the questionnaire before the survey (Cronbach’s alpha=0.865).

Data collection and Analysis

This study was approved by the ethics committee of the study hospital. Data were collected on May 23－31, 2022. All participants were invited to complete the questionnaire through WeChat, DingTalk, and Tencent QQ from May 23 to May 31, 2022. A professional questionnaire survey platform which provides functions equivalent to Amazon Mechanical Turk called “Wenjuan Xing” was used to investigate. The researcher sent the questionnaire through WeChat, DingTalk, and Tencent QQ to colleagues and classmates to fill in and asked them to forward the questionnaire to their colleagues. Data were analyzed using SPSS for Windows, version 23. Statistical significance was set at $p < 0.05$. The findings were summarized using descriptive statistics, univariate analysis, and dummy multiple regression analysis. The data were normally distributed, as assessed by skewness and kurtosis tests. Descriptive statistics (e.g., mean, SD or n, %) were used to summarize the study variables. Univariate analysis (independent-samples t-test and one-way analysis of variance for categorical independent variables) was performed to explore the potential predictors of medical staff KAB for dysphagia assessment. A comparison analysis was conducted to analyze the differences in medical staff’s KAB for dysphagia assessment among Guangdong Province, Hunan Province, Guangxi Province, and Shaanxi Province.

Results

Characteristics of the Participants

A total of 430 nurses and doctors were enrolled, of which 353 nurses and doctors were valid. Clinical nurses, clinicians, and managers accounted for 66.29%, 19.26%, and 9.63%, respectively. Of the included hospitals, 81.02% were tertiary hospitals. Other characteristics, such as department, medical staff work, and learning experience, are summarized in Table 1.

Table 1. Demographic characteristics of the study participants(n=353)

Characteristics		Guangdong n=211	Hunan n=77	Shaanxi n=40	Total n(%) n=353
hospital level	level 1 hospital	13(6.16)	1(1.30)	0(0.00)	14(3.97)
	level 2 hospital	49(23.22)	3(3.40)	0(0.00)	53(15.01)
	level 3 hospital	149(70.62)	73(95.30)	40(100.00)	286(81.02)
hospital type	the general hospital	194(91.94)	77(100.00)	40(100.00)	336(95.18)
	other hospital	17(8.06)	0(0.00)	0(0.00)	17(4.82)
department	neurological, rehabilitation, elderly related department	80(37.91)	39(50.65)	38(95.00)	177(50.14)
	other department	131(62.09)	38(49.35)	2(5.00)	176(49.86)
position	clinical nurse	131(62.09)	51(66.23)	38(95.00)	234(66.29)
	clinical doctor	35(16.59)	23(29.87)	0(0.00)	68(19.26)
	Management personnel	30(14.22)	2(2.60)	1(2.50)	34(9.63)
	Community nurses	12(5.69)	0(0.00)	0(0.00)	12(3.40)
	others	3(1.41)	1(1.30)	1(2.50)	5(1.42)
title	primary title	94(44.55)	36(46.75)	23(57.50)	165(46.74)
	medium-grade professional title	91(43.13)	30(38.96)	17(42.50)	148(41.93)
	senior title of professional	26(12.32)	11(14.29)	0(0.00)	40(11.33)
working years in the field of dysphagia related diseases	none	87(41.23)	13(16.88)	7(17.50)	113(32.01)
	<3 years	32(15.17)	24(31.17)	5(12.50)	63(17.85)
	3-5 years	28(13.27)	12(15.58)	8(20.00)	54(15.30)
	≥5 years	64(30.33)	28(36.37)	20(50.00)	123(34.84)
education	Junior college and below	43(20.38)	18(23.38)	0(0.00)	62(17.56)
	Bachelor	145(68.72)	53(68.83)	39(97.50)	253(71.67)
	Master degree or above	23(10.90)	6(7.84)	1(2.50)	38(10.77)
nursing experience of patients with dysphagia	yes	137(64.93)	44(57.14)	35(87.50)	234(66.29)
	no	74(35.07)	33(42.86)	5(12.50)	119(33.71)
related training for dysphagia	yes	109(51.66)	28(36.36)	29(72.50)	175(49.58)
	no	102(48.34)	49(63.64)	11(27.50)	178(50.42)
specialized training in geriatric, swallowing and rehabilitation	yes	31(14.69)	6(7.79)	14(35.00)	55(15.58)
	no	180(85.31)	71(92.21)	26(65.00)	298(84.42)

KAB scores and scoring rate

Means and their scoring rates of the measurement outcome are listed in Table 2. The knowledge and behavior scores were medium, but the attitude scores were high.

Table 2. Participants' measured Mean scores of *Knowledge, Attitudes and Behavior* in dysphagia assessment

Instruments and score range	Mean(SD)	percentige rating score	Minimum	Maximum	Range
medical staff's Knowledge of dysphagia assessment (0-24.4)	15.34(2.66)	62.87	3.6	22.20	18.60
medical staff's Attitude of dysphagia assessment(8-40)	35.89(4.86)	89.73	8.00	40.00	32.00
medical staff's Behavior of dysphagia assessment(13-65)	41.42(14.43)	63.72	13.00	65.00	52.00
medical staff's KAB of dysphagia assessment(21-129.4)	92.65(17.52)	71.60	37.00	125.00	88.00

Note. KAB=Knowledge, Attitudes and Behavior

KAB Differences in 4 province

As seen in Table 3, medical staff in Shaanxi Province were more willing to assess dysphagia and were evaluated more frequently.

Table 3. Differences of medical staff 's *Knowledge, Attitudes and Behavior* of dysphagia assessment in different province(one-way analysis of variance)

Instruments and subdomains	Guangdong	Hunan	Guangxi	Shaanxi	*F*	*p* values
	Mean(SD)					
medical staff's Knowledge of dysphagia assessment(0-24.4)	15.37(2.51)	14.91(2.39)	16.58(2.47)	15.24(3.69)	2.519	0.058
medical staff's Attitude of dysphagia assessment(8-40)	35.65(5.07)	35.23(4.73)	37.12(4.06)	37.68(3.96)	3.023	0.030*
medical staff's Behavior of dysphagia assessment(13-65)	40.80(15.46)	38.49(12.02)	43.48(13.06)	49.05(11.07)	5.273	0.001**
medical staff's KAB of dysphagia assessment(21-129.4)	91.82(18.74)	88.64(14.23)	97.18(14.64)	101.97(14.67)	6.080	<0.001***

Note.*Medical staff in Shaanxi province were more willing to assess dysphagia than Guangdong(p=0.037) and Hunan(p=0.023)

**Medical staff in Shaanxi province were more frequently evaluated for dysphagia than Guangdong(p=0.001) and Hunan(p<0.001)

***The scores of the questionnaire in Shaanxi province was higher than Guangdong(p=0.002) and Hunan(p<0.001)

KAB=Knowledge, Attitudes and Behavior

Univariate analysis

KAB levels were compared among the different demographic subgroups. Detailed findings are presented in Table 4. The KAB of medical staff is not significantly different between positions, titles, and education.

Table 4. Differences of medical staff 's *Knowledge, Attitudes and Behavior* of dysphagia assessment in Sociodemographic, training and working experience characteristics(n=353)

Characteristic		n(%)	KAB mean(SD)	95%*CI*	Univariate analysis (*t/F, p*)
hospital level	level 1 hospital	14(3.97)	81.31(16.78)	(71.63,91.00)	*F*=4.680, *p*=0.010
	level 2 hospital	53(15.01)	89.26(19.11)	(83.99,94.52)	
	level 3 hospital	286(81.02)	93.84(17.02)	(91.86,95.82)	
hospital type	the general hospital	336(95.18)	93.39(17.18)	(91.55,95.24)	*t*=3.585, *p*<0.001
	other hospital	17(4.82)	78.04(18.24)	(68.66,87.41)	
department	neurological, rehabilitation, elderly related department	177(50.14)	100.59(13.55)	(98.58,102.60)	*t*=9.566, *p*<0.001
	other department	176(49.86)	84.67(17.46)	(82.07,87.27)	
position	clinical nurse	234(66.29)	93.64(17.01)	(91.45,95.83)	*F*=1.126, *p*=0.344
	clinical doctor	68(19.26)	91.12(18.72)	(86.59,95.65)	
	Management personnel	34(9.63)	92.50(16.88)	(86.61,98.39)	
	Community nurses	12(3.40)	87.28(22.72)	(72.85,101.72)	
	others	5(1.42)	81.28(13.25)	(64.83,97.73)	
title	primary title	165(46.74)	92.15(17.59)	(89.45,94.85)	*F*=0.342, *p*=0.710
	medium-grade professional title	148(41.93)	92.66(17.59)	(89.80,95.52)	
	senior title of professional	40(11.33)	94.71(17.22)	(89.20,100.22)	
working years in the field of dysphagia related diseases	none	113(32.01)	82.46(16.95)	(79.30,85.62)	*F*=24.700, *p*<0.001
	<3 years	63(17.85)	93.17(17.62)	(88.73,97.60)	
	3-5 years	54(15.30)	98.06(15.79)	(93.75,102.37)	
	⩾5 years	123(34.84)	99.39(14.16)	(96.86,101.91)	
education	Junior college and below	62(17.56)	89.70(19.42)	(84.77,94.63)	*F*=1.148, *p*=0.319
	Bachelor	253(71.67)	93.44(16.97)	(91.34,95.54)	
	Master degree or above	38(10.77)	92.24(17.79)	(86.39,98.09)	
experience of dysphagia patients' nursing	yes	234(66.29)	97.78(15.09)	(95.83,99.72)	*t*=8.019, *p*<0.001
	no	119(33.71)	82.58(17.65)	(79.38,85.78)	

related training for dysphagia	yes	175(49.58)	99.86(14.55)	(97.69,102.03)	*t*=8.397, *p*<0.001
	no	178(50.42)	85.57(17.33)	(83.01,88.13)	
specialized training in geriatric, swallowing and rehabilitation	yes	55(15.58)	106.20(12.31)	(102.87,109.52)	*t*=8.285, *p*<0.001
	no	298(84.42)	90.16(17.20)	(88.19,92.12)	

Note.Abbreviation: CI=confidence interval, KAB=Knowledge, Attitudes and Behavior.

Predictors of medical staff's KAB of dysphagia assessment

In the final regression analysis, all the categorical variables were transformed into dummy variables. Table 5 presents the dummy variables' settings. Dummy multiple regression analysis was conducted to identify relevant predictors for medical staff KAB in dysphagia assessment. As seen in Table 6, working years in the field of dysphagia related diseases, specialized training in geriatric, swallowing, and rehabilitation; nursing experience of patients with dysphagia; neurological, rehabilitation, elderly related department; and related training for dysphagia were significant predictors of medical staff's KAB of dysphagia assessment, accounting for 35.1% of variance.

Table 5. Dummy variable settings

the original variable		D1	D2	D3	D4	state
hospital level	level 1 hospital	1	0			
	level 2 hospital	0	0			reference category
	level 3 hospital	0	1			
hospital type	the general hospital	1				
	other hospital	0				reference category
department	neurological, rehabilitation, elderly related department	1				
	other department	0				reference category
position	clinical nurse	1	0	0	0	
	clinical doctor	0	0	0	0	reference category
	Management personnel	0	0	1	0	
	Community nurses	0	0	0	1	
	others	0	1	0	0	
title	primary title	1	0			
	medium-grade professional title	0	0			reference category
	senior title of professional	0	1			
working years in the field of dysphagia related diseases	none	1	0	0		
	<3 years	0	0	0		reference category
	3-5 years	0	1	0		
	⩾5 years	0	0	1		
education	Junior college and below	1	0			
	Bachelor	0	0			reference category
	Master degree or above	0	1			
nursing experience of patients with dysphagia	yes	1				
	no	0				reference category

related training for dysphagia	yes	1	
	no	0	reference category
specialized training in geriatric, swallowing and rehabilitation	yes	1	
	no	0	reference category

Note.D: dummy variable

Table 6. Factors related to *Knowledge, Attitudes and Behavior* by stepwise regression

Variables	Std. *β*	*t*	*p* value
hospital level(1 level)	-0.026	-0.579	0.563
hospital level(3 level)	0.045	1.025	0.306
hospital type(the general hospital)	0.065	1.470	0.142
working years in the field of dysphagia related diseases(none)	-0.130	-2.530	0.012
working years in the field of dysphagia related diseases(3-5 years)	0.020	0.449	0.654
working years in the field of dysphagia related diseases(⩾5 years)	0.023	0.454	0.650
specialized training in geriatric, swallowing and rehabilitation(yes)	0.148	3.171	0.002
nursing experience of patients with dysphagia(yes)	0.172	3.491	0.001
neurological, rehabilitation, elderly related department(yes)	0.239	4.631	<0.001
related training for dysphagia(yes)	0.196	4.026	<0.001

note. Std. β, standardized β coefficient

Adjusted R^2=0.351, F=39.130, p<0.001

Discussion

Status of KAB of dysphagia assessment

In our study, the KAB scores were 92.65±17.52, and the percentile rating score was 71.60. The percentile rank was 53.99, which means that 53.99% of participants' KAB scores were above the average score. According to the percentile rating score in Table 2, Knowledge and Behavior scores were medium, and attitude scores were high. This suggests that people are willing to assess swallowing disorders, but lack Knowledge and Behavior.

The lack of knowledge in our study is reflected in the following aspects: First, 80.41% and 76.29% participants did not know how to perform the volume-viscosity swallowing test (VVST) and water swallow test, respectively. This is related to the fact that 84.42% of the participants did not have specialized training in geriatric, swallowing, and rehabilitation. Second, nearly half of the participants did not know the concept of mouthful size, food requirements for patients with dysphagia, or how to feed patients with hemiplegia. An audit[17] conducted in our inpatient hospice showed that 57% of nurses followed prescribed dysphagia management. The

deficiencies included using the wrong utensils, incorrect positioning of the patient during feeding, or giving patients fluids or food of the wrong consistency. This rate is similar to an informal observation of 50% compliance published by the creator of the Mealtime and Dysphagia Questionnaire (MDQ)[18].

The lack of behavior in our study is mainly reflected in the following aspects. First, in 79.12% of the participants' departments, when stroke patients were screened for dysphagia, no further instrumental examinations were performed. Second, in 13.92% of the participants' departments, swallowing function was not screened in stroke patients after admission. Nurse stress with patient characteristics and workload may affect whether a swallow screen is undertaken [19]. The time of screening or assessment of OD prevalence was recorded. OD prevalence estimates from the hospital setting were reported as the time post-stroke or time from admission. Time post-stroke ranged from the hyperacute phase[20]: ≤24h post-stroke, to the acute phase[21]; 1-7 days, to the early subacute phase[22];7 days-3 months, to the recovery phase[23]. Therefore, we should screen for or assess dysphagia at the right time based on the patient's condition and identify reliable and standardized assessment methods for screening post-stroke dysphagia(PSD) in acute stroke patients to ensure reliable estimates [24].

One study reported that 35.8% of professionals did not know the definition of dysphagia as a swallowing disorder[25]. Lack of knowledge among healthcare professionals can lead to inappropriate practices and increase the complications of dysphagia, such as aspiration pneumonia and malnutrition. This is an important barrier in the management of patients with dysphagia. One study investigated the Knowledge, Attitude and Practice of healthcare providers in Iran, and the results showed that very few participants were familiar with a standard test for screening and assessment of dysphagia (11.9%). A total of 74.7% were willing to participate in a workshop on dysphagia; the main pitfalls in their country lie in practice [26]. Another study reported that there seems to be limited awareness among ICU practitioners that patients are at risk of dysphagia, particularly as ventilation persists, protocols, routine assessment, and instrumental assessments are generally not used[27].

Comparison of medical staff’s KAB in different Provinces

In our study, the KAB of participants in Shaanxi Province was higher than that of participants in other provinces. The reasons for this are as follows. First, 95% of the participants in Shaanxi Province came from neurological, rehabilitation, and elderly related departments that mainly treat patients with stroke. There are many situations in the workplace that require the assessment of swallowing disorders. Second, 87.5% of participants in Shaanxi Province had experience in nursing patients with dysphagia.

Developing training resources by predictors of KAB of dysphagia assessment

There is still plenty of room to improve medical staff’s Knowledge, Attitudes and Behavior regarding dysphagia assessment. Multiple linear regression results indicated

that *experience of dysphagia patients' nursing, related training for dysphagia, working years in the field of dysphagia related diseases, specialized training in geriatric, swallowing & rehabilitation* and, *neurological, rehabilitation, elderly related department* were significant predictors. Consequently, the following countermeasures were proposed: The following countermeasures were based on the theory of Knowledge-Attitude-Practice and Self-efficacy:

Strengthening relevant training to improve Knowledge of dysphagia assessment

Knowledge-attitude-practice (KAP) theory is widely used in behavioral psychology[28]. This theory emphasizes that change in an individual's behavior consists of three continuous processes: acquiring knowledge, establishing beliefs and producing behavior, and paying attention to their causality and progressive relationship. The diagnosis and management of dysphagia requires comprehensive knowledge of diverse etiologies, with a systematic approach for the assessment of symptoms, selection of investigations, and appropriate treatment to relieve symptoms[29]. Meanwhile, staff who provide mealtime assistance to people with dysphagia require adequate training to ensure that mealtimes are safe and enjoyable[30]. In this study, knowledge consisted of an assessment approach, dysphagia symptoms, mealtime assistance, and so on. The mean score of medical staff was 15.34±2.66, which was at the middle level, and 49.58% participants had received related training for dysphagia(35.80% in other departments VS 63.28% in neurological, rehabilitation, and elderly related departments). Only 15.58% of the participants had received specialized training in geriatric, swallowing, and rehabilitation(8.52% in other departments VS 22.60% in neurological, rehabilitation, and elderly related departments). Medical staff in neurological, rehabilitation, and elderly related departments seldom receive related training for dysphagia, which leads to less knowledge of assessment or screening for dysphagia. Therefore, it is more difficult for medical staff to establish positive beliefs regarding dysphagia assessment and screening. To standardize the management of dysphagia, hospitals should take the rate of dysphagia assessment as a quality control index and promote training in dysphagia assessment. Examples include the Volume-Viscosity Swallow Test(V-VST), water swallow test, eating assessment Tool-10(EAT-10), and Mann Assessment of Swallowing Ability (MASA)..

Establishing positive belief in assessment of dysphagia

According to KAP theory, beliefs and attitudes are the motive forces for individuals to produce related behaviors[28]. As the results of study, the attitude score of medical staff was 35.89±4.86, which was at the high level. This finding suggests that many people are willing to screen and evaluate patients with dysphagia. This was a good trend. Nurses gained a sense of value by assessing and caring for patients with dysphagia. Dysphagia screening is often the focus of hospitalized stroke patients; however, dysphagia can also occur in other hospitalized patients and outpatients. Dysphagia can

be overlooked by nurses and clinicians, and it is therefore important to educate nurses on the importance of dysphagia screening [31].

Accumulate dysphagia assessment experience through work and study

Self-efficacy refers to an individual's conviction of their capacity to perform a specific activity. Individuals gain self-efficacy over time as they acquire a range of talents, such as social, cognitive, physical, and linguistic abilities, via life experiences[32]. Efficacy expectations are dynamic and are both appraised and enhanced by four mechanisms[33-34]: (1) enactive mastery experience or successful performance of the activity of interest; (2) verbal persuasion or encouragement, given by a credible source that the individual is capable of performing the activity of interest; (3) vicarious experience or seeing like individuals perform a specific activity; and (4) physiological and affective states such as pain, fatigue, anxiety, hunger, or dizziness associated with a given activity. These mechanisms to drive efficacy expectations are the concepts upon which the intervention is built to encourage behavioral change. In this study, *experience of dysphagia patients' nursing, working years in the field of dysphagia related diseases,* and *neurological, rehabilitation, elderly related department* were significant predictors of medical staff's Knowledge, Attitudes and Behavior of dysphagia assessment. The results are consistent with the self-efficacy theory. This suggests that if conditions permit, the medical staff in other departments with no experience of dysphagia patients' nursing should transfer to neurological, rehabilitation, and elderly related departments to be trained and accumulate relevant experience. Alternatively, the hospital organizes regular workshops for dysphagia assessment and nurses are required to participate in practice.

Establish systematic inter-professional collaboration in dysphagia management

A study showed that the main theme "limited professional services" describes how patients received little support from healthcare professionals and had to rely on themselves to adapt to life with dysphagia[35]. The common goal of preventing aspiration and rehabilitating patients' ability to swallow safety is based on dysphagia assessment, using appropriate therapeutic interventions, sharing knowledge, and improving skills among professional groups that consist of nurses, physicians, occupational therapists, and speech-language pathologists(SLPs)[36].

Conclusions and Implications for future Research

Treatment pathways, including early assessment and diagnosis of dysphagia, should be a priority for healthcare professionals working in different settings with populations at risk for dysphagia. Most studies have focused on medical staff in hospitals, and few have focused on medical staff from nursing home settings, social health service centers, and palliative care facilities. Future studies should focus on the

evaluation of dysphagia and provide training for the assessment of swallowing disorders to medical staff in nursing home settings, social health service centers, and palliative care facilities.

Conflict of interest statement

The authors declare that there are no conflict of interests, we do not have any possible conflicts of interest.

Data access statement

The data that support the findings of this study are available from the corresponding author, [Juanhui Chen], upon reasonable request.

Acknowledgements

We would like to thank Shenzhen Longgang District Science and Technology Innovation Bureau for providing the Unfunded Medical and Health Technology Plan(No. LGKCYLWS2020128).

Author contributions

Conceptualization, Statistical analysis, Writing-review & editing: Juanhui Chen
Investigation: Juanhui Chen, Wenqiu Ye, Xingyun Zheng, Wenna Wu, Yuebao Chen, Yinjuan Chen

References

[1] Johnson, C. O., Nguyen, M., Roth, G. A., et al. (2019). Global, regional, and national burden of stroke, 1990– 2016: a systematic analysis for the Global Burden of Disease Study 2016. Lancet Neurology, 18(5), 439–458. https://doi.org/10.1016/S1474-4422(19)30034-1

[2] Rugiu MG. Role of videofluoroscopy in evaluation of neurologic dysphagia[J]. Acta Otorhinolaryngol Ital, 2007,27(6):306-16. PMID: 18320837; PMCID: PMC2640050.

[3] Matsumura T, Mitani Y, Oki Y, et al. Risk factors for the onset of aspiration pneumonia among stroke patients in the recovery stage[J]. Nihon Ronen Igakkai Zasshi, 2014,51(4):364-8.doi: 10.3143/geriatrics.51.364. PMID: 25327371.

[4] Edmiaston J, Connor LT, Loehr L, et al. Validation of a dysphagia screening tool in acute stroke patients[J]. Am j Crit Care, 2010,19(4):357-64.doi: 10.4037/ajcc2009961. Epub 2009 Oct 29. PMID: 19875722; PMCID: PMC2896456.

[5] Baijens LW, Clave P, Cras P, et al. European society for swallowing disorders - European union geriatric medicine society white paper: oropharyngeal dysphagia as a geriatric syndrome. Clin Interv Aging. 2016 Oct 7;11:1403-1428. doi: 10.2147/CIA.S107750. PMID: 27785002; PMCID: PMC5063605.

[6] Rivelsrud Maribeth Caya, Hartelius Lena, Bergström Liza et al. Prevalence of Oropharyngeal Dysphagia in Adults in Different Healthcare Settings: A Systematic Review and Meta-analyses.[J] .Dysphagia. 2023 Feb;38(1):76-121. doi: 10.1007/s00455-022-10465-x. Epub 2022 May 31. PMID: 35639156; PMCID:

PMC9873728.
[7] Blanař V, Hödl M, Lohrmann C, et al. Dysphagia and factors associated with malnutrition risk: a 5-year multicentre study. J Adv Nurs.2019;75(12):3566–76.doi: 10.1111/jan.14188. Epub 2019 Sep 10. PMID: 31452231.
[8] Huppertz VAL, Halfens RJG, van Helvoort A, et al. Association between oropharyngeal dysphagia and malnutrition in Dutch nursing home residents: results of the national prevalence measurement of quality of care. J NutrHealth Aging. 2018;22(10):1246–52.doi: 10.1007/s12603-018-1103-8. PMID: 30498833; PMCID: PMC6302767.
[9] Beharry A, Michel P, Faouzi M, et al. Predictive factors of swallowing disorders and bronchopneumonia in acute ischemic stroke. J Stroke Cerebrovasc Dis. 2019;28(8):2148–54.doi: 10.1016/j.jstrokecerebrovasdis.2019.04.025. Epub 2019 May 22. PMID: 31129105.
[10] Speyer R, Cordier R, Farneti D, et al. White paper by the European Society for swallowing disorders: screening and non-instrumental assessment for dysphagia in adults. Dysphagia. 2021;37:333-349.doi: 10.1007/s00455-021-10283-7. Epub 2021 Mar 31. PMID: 33787994; PMCID: PMC8009935.
[11] Davis Lori, Copeland Karen. Effectiveness of computer-based dysphagia training for direct patient care staff.[J] .Dysphagia, 2005, 20: 141-8.doi: 10.1007/s00455-005-0007-z. PMID: 16172824.
[12] Smith Pamela A. Nutrition, hydration, and dysphagia in long-term care: Differing opinions on the effects of aspiration.[J] .J Am Med Dir Assoc, 2006, 7: 545-9.doi: 10.1016/j.jamda.2006.03.008. Epub 2006 May 30. PMID: 17095418.
[13] Tan Laurence, Gan Grace, Hum Allyn et al. A Stepwise, Mixed-Method Study Approach to Identify the Barriers to Dysphagia Care in Hospice Care Nurses.[J] .J Hosp Palliat Nurs, 2018, 20: 88-94.doi: 10.1097/NJH.0000000000000404. PMID: 30063619.
[14] Luo, H., & He, Z. H. . Reliability and validity of psychological capital questionnaire in nurses. Chinese Journal of Behavioral Medical and Brain Science, 2010,19(9):853-854. DOI:10.3760/cma.j.issn.1674-6554.2010.09.028..
[15] Dong X F. Evidence-based management of stroke patients with dysphagia and its effect evaluation [D]. Zhengzhou University,2018.
[16] Ma Ke, GUO Yuanli, DONG Xiaofang, et al. Investigation on the status of screening and evaluation of dysphagia in stroke patients by nurses in Henan Province[J]. Chinese journal of nursing,2020,55(08):1196-1200. DOI:10.3761/j.issn.0254-1769.2020.08.016.
[17] Tan L, Gan G, Hum A, et al. Honey I fed my patients: clinical improvement project on swallowing safety. Presented as a poster at the Singapore Intermediate Long Term Care Quality Conference; 2013.
[18] Colodny N. Construction and validation of the Mealtime and Dysphagia Questionnaire: an instrument designed to assess nursing staff reasons for noncompliance with SLP dysphagia and feeding recommendations. Dysphagia. 2001;16(4):263-271.doi: 10.1007/s00455-001-0085-5. PMID: 11720402.
[19] Moghadam Kolsoom Nasirizad,Chehrzad Minoo Mitra,Masouleh Shademan

Reza et al. Nursing workload in intensive care units and the influence of patient and nurse characteristics.[J] .Nurs Crit Care, 2021, 26: 425-431.doi: 10.1111/nicc.12548. Epub 2020 Sep 20. PMID: 32954619.
[20] Bernhardt J, Hayward KS, Kwakkel G, et al. Agreed definitions and a shared vision for new standards in stroke recovery research: the stroke recovery and rehabilitation roundtable taskforce. Int J Stroke. 2017;12(5):444–50.doi: 10.1177/1747493017711816. PMID: 28697708.
[21] Paciaroni M, Mazzotta G, Corea F, et al. Dysphagia following Stroke. Eur Neurol.2004;51(3):162–7.doi: 10.1159/000077663. Epub 2004 Apr 1. PMID: 15073441.
[22] Abubakar SA, Jamoh BY. Dysphagia following acute stroke and its efect on short-term outcome. Niger Postgrad Med J.2017;24(3):182–6.doi: 10.4103/npmj.npmj_96_17. PMID: 29082909.
[23] Baroni AF, Fabio SR, Dantas RO. Risk factors for swallowing dysfunction in stroke patients. Arq Gastroenterol.2012;49(2):118–24.doi: 10.1590/s0004-28032012000200005. PMID: 22766998.
[24] Banda Kondwani Joseph, Chu Hsin, Kang Xiao Linda et al. Prevalence of dysphagia and risk of pneumonia and mortality in acute stroke patients: a meta-analysis.[J] .BMC Geriatr, 2022, 22: 420.doi: 10.1186/s12877-022-02960-5. PMID: 35562660; PMCID: PMC9103417.
[25] Sánchez-Sánchez, E., Avellaneda-López, Y., García-Marín, E., et al. Knowledge and practice of health professionals in the management of dysphagia. International Journal of Environmental Research and Public Health, 2021 Feb 22;18(4):2139. doi: 10.3390/ijerph18042139. PMID: 33671732; PMCID: PMC7926391.
[26] Farpour Sima, Farpour Hamid Reza, Smithard David et al. Dysphagia Management in Iran: Knowledge, Attitude and Practice of Healthcare Providers.[J] .Dysphagia, 2019, 34: 105-111.doi: 10.1007/s00455-018-9919-2. Epub 2018 Jun 21. PMID: 29931606.
[27] Spronk Peter E, Spronk Laura E J, Egerod Ingrid et al. Dysphagia in Intensive Care Evaluation (DICE): An International Cross-Sectional Survey.[J] .Dysphagia, 2022 Dec;37(6):1451-1460. doi: 10.1007/s00455-021-10389-y. Epub 2022 Jan 29. PMID: 35092486.
[28] World Health Organization & Stop T.B.Partnership, 2008. Advocacy, communication and social mobilization for TB control: a guide to developing knowledge, attitude and practice surveys. World Health Organization. https://apps.who.int/iris/handle/10 665/43790.
[29] Nigam Gaurav B, Vasant Dipesh Harshvadan, Dhar Anjan. Curriculum review : investigation and management of dysphagia.[J] .Frontline Gastroenterol, 2022, 13: 254-261.doi: 10.1136/flgastro-2021-101917. PMID: 35493628; PMCID: PMC8996094.
[30] Reddacliff Courtney, Hemsley Bronwyn, Smith Rebecca, et al. Examining the Content and Outcomes of Training in Dysphagia and Mealtime Management: A Systematic Review Informing Co-Design of New Training.[J] .Am J Speech Lang Pathol, 2022, 31: 1535-1552.doi: 10.1044/2022_AJSLP-21-00231. Epub 2022 Apr 4.

PMID: 35377733.
[31] West James, Stutzman Sonja E, Atem Folefac, et al. Formatting an experiential learning education module to encourage dysphagia assessment in apheresis patients.[J] .J Clin Apher, 2018, 33: 72-77.doi: 10.1002/jca.21572. Epub 2017 Aug 2. PMID: 28766740.
[32] Huo C, Hameed J, Nawaz A, Adnan Raheel Shah S, et al. Scientific risk performance analysis and development of disaster management framework: a case study of developing Asian countries. J King Saud Univ Sci. 2021 Mar;33(2):101348. doi: 10.1016/j.jksus.2021.101348. Epub 2021 Jan 28. PMID: 35495615; PMCID: PMC9042590.
[33] Bandura A. Self-Efficacy: The Exercise of Control. New York: WH Freeman; 1997.
[34] Resnick B. Geriatric rehabilitation: the influence of efficacy beliefs and motivation. Rehabil Nurs. 2002;27(4):152–159.doi: 10.1002/j.2048-7940.2002.tb02224.x. PMID: 12116528.
[35] Li Chen, Qiu Chunmei,Shi Ying et al. Experiences and perceptions of stroke patients living with dysphagia: A qualitative meta-synthesis[J]. J Clin Nurs, 2022, 31: 820-831.doi: 10.1111/jocn.15995. Epub 2021 Aug 8. PMID: 34369025.
[36] Nielsen Anne Højager, Kaldan Gudrun, Nielsen Birthe Husted et al. Intensive care professionals' perspectives on dysphagia management: A focus group study.[J] .Aust Crit Care, 2023 Jul;36(4):528-535. doi: 10.1016/j.aucc.2022.04.004. Epub 2022 May 21. PMID: 35610091.
